# Supplementary material for: Direct Electrooxidative Selenylation/Cyclization of Alkynes: Access to Functionalized Benzo[b]furans
Source: Molecules. 2022 Sep 25;27(19):6314. doi: 10.3390/molecules27196314 (PMC9572441; doi:10.3390/molecules27196314)
Supplement: Supplementary file 1 [file molecules-27-06314-s001.zip › molecules-1904585-supplementary.pdf]

Supporting Information

Direct Electrooxidative Selenylation/Cyclization of Alkynes: Access to Functionalized Benzo[*b*]furans

Balati Hasimujiang,+ Shengsheng Lin,+ Chengwei Zheng, Yong Zeng, Zhixiong Ruan*

Guangzhou Municipal and Guangdong Provincial Key Laboratory of Molecular Target & Clinical Pharmacology and the State & NMPA Key Laboratory of Respiratory Disease, School of Pharmaceutical Sciences & the Fifth Affiliated Hospital, Guangzhou Medical University, Guangzhou, 511436, P.R.China.

Email: zruan@gzhmu.edu.cn

+ These authors contributed equally to this work.

Table of Contents

The setup of electrochemical reaction S-2
Optimization Studies S-3
Gram-scale Synthesis S-4
Control Experiments S-5
Cyclic Voltammetry Experiment S-8
Crystallographic Details S-9
NMR Spectra S-15

The setup of electrochemical reaction

(a)

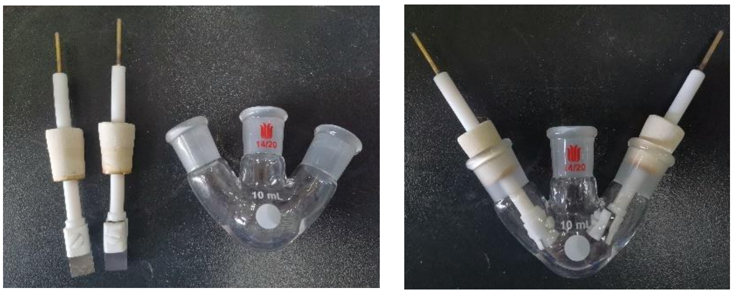

(b)

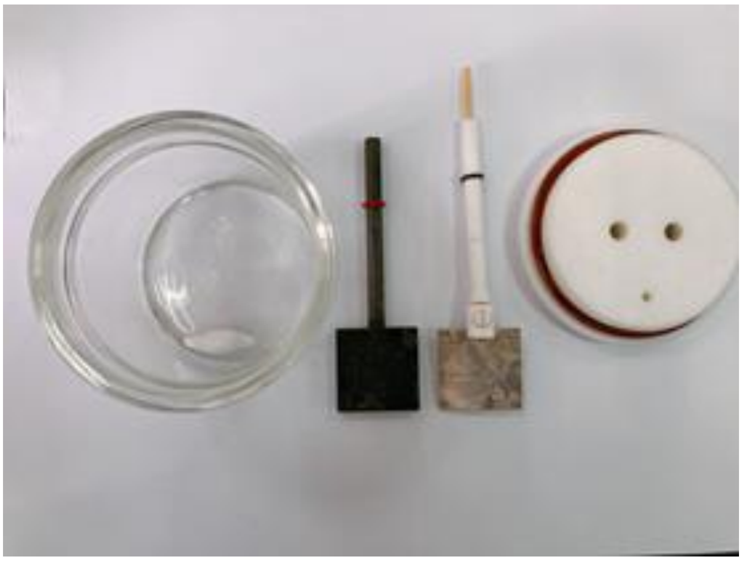

Figure S1. The setup of electrochemical reaction. (a) Equipment of standard reaction; (b) Equipment of gram-scale reaction.

Optimization Studies

Table S1. Optimization of the Reaction Conditions[a]

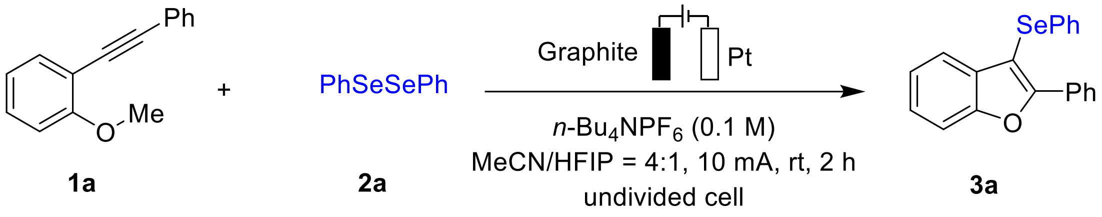

Entry	Deviation from standard conditions	Yield(%)[b]
**1**	**None**	**96**
2	MeOH as solvent (6 h)	76
3	MeCN as solvent (6 h)	92
4	Anhydrous MeCN	90
5	Anhydrous MeCN, N_2	89
6	MeCN/HFIP = 4:1, N_2	93
7	MeCN/H_2O = 4:1	25
8	*n*-Bu_4NClO_4 instead of *n*-Bu_4NPF_6	85
9	*n*-Bu_4NBF_4 instead of *n*-Bu_4NPF_6	85
10	$LiClO_4$ instead of *n*-Bu_4NPF_6	75
11	C(+)-C(-)	85
12	Pt plate as anode	93
13	5 mA instead of 10 mA (6h)	87
14	**2a** (0.18 mmol) instead of **2a** (0.36 mmol)	81
15	no electrolyte	0
16	no electric current	0

[a] Reaction conditions: undivided cell, graphite anode (1.5 cm × 1.0 cm × 0.2 cm), Pt cathode (1.0 cm × 1.0 cm × 0.01 cm), **1a** (0.3 mmol), **2a** (0.36 mmol), *n*-Bu_4NPF_6 (0.5 mmol), MeCN/HFIP (4:1, 5.0 mL), constant current = 10.0 mA, 2 h (2.5 F mol^{-1}), under air, at room temperature. [b] Yields of isolated products.

Gram-scale Synthesis

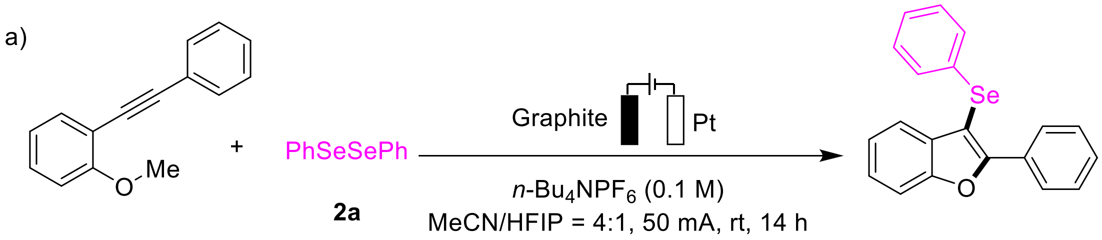

1a, 15 mmol

3a, 4.45 g, 85%

In an undivided cell (250 mL) equipped with a stir bar, a mixture of substrates **1a** (15.0 mmol, 3.12 g), **2a** (18.0 mmol, 5.62 g), *n*-Bu_4NPF_6 (25.0 mmol, 9.69 g) and MeCN/HFIP = 4:1 (150 mL) were added. The cell was equipped with a graphite plate (3 cm x 3 cm x 0.6 cm) as the anode and platinum plate (3 cm x 3 cm x 0.01 cm) as the cathode and connected to a DC regulated power supply. The reaction mixture was stirred and electrolyzed at a constant current of 50 mA at 23 °C bath for 14 h. When the reaction was finished, the mixture was concentrated under reduced pressure. Purification by column chromatography on silica gel (eluent: petroleum ether) yielded solo product **3a** (4.45 g, 85%) as yellow solid.

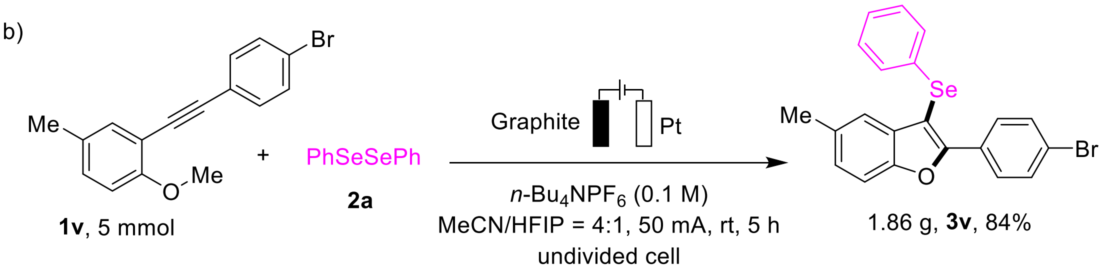

1v, 5 mmol

1.86 g, **3v**, 84%

In an undivided cell (250 mL) equipped with a stir bar, a mixture of substrates **1v** (5.0 mmol, 1.50 g), **2a** (6.0 mmol, 1.87 g), *n*-Bu_4NPF_6 (8.3 mmol, 3.23 g) and MeCN/HFIP = 4:1 (150 mL) were added. The cell was equipped with a graphite plate (3 cm x 3 cm x 0.6 cm) as the anode and platinum plate (3 cm x 3 cm x 0.01 cm) as the cathode and connected to a DC regulated power supply. The reaction mixture was stirred and electrolyzed at a constant current of 50 mA at 23 °C bath for 5 h. When the reaction was finished, the mixture was concentrated under reduced pressure. Purification by column chromatography on silica gel (eluent: petroleum ether) yielded solo product **3v** (1.86 g, 84%) as white solid.

Control Experiments

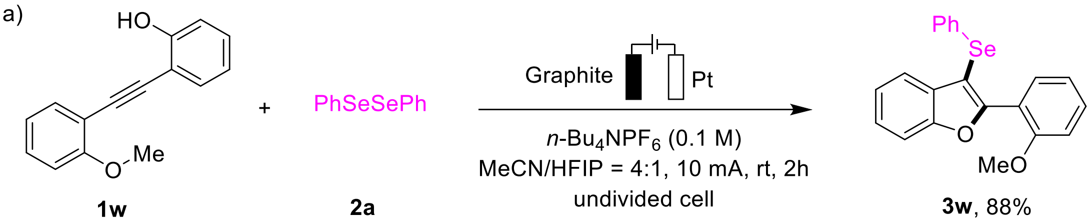

In an undivided cell (20 mL) equipped with a stirring bar, a mixture of **1w** (0.3 mmol), **2a** (0.36 mmol), *n*-Bu_4NPF_6 (0.5 mmol) and MeCN/HFIP = 4:1 (5 mL) were added. The cell was equipped with graphite plate (1.5 cm × 1 cm × 0. 2 cm) as the anode and platinum plate (1 cm × 1 cm × 0.01 cm) as the cathode and connected to a DC regulated power supply. The reaction mixture was stirred and electrolyzed at a constant current of 10 mA at room temperature for 2 h. When the reaction was finished, the mixture was concentrated under reduced pressure. Purification by column chromatography on silica gel (eluent: petroleum ether) yielded product **3w** (100 mg, 88%) as yellow oil.

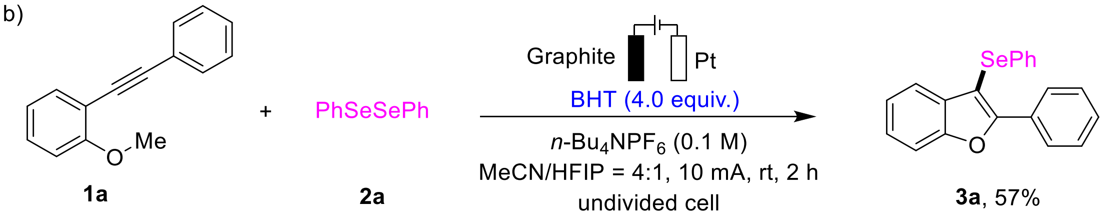

In an undivided cell (20 mL) equipped with a stirring bar, a mixture of **1a** (0.3 mmol), **BHT** (1.2 mmol), **2a** (0.36 mmol), *n*-Bu_4NPF_6 (0.5 mmol) and MeCN/HFIP = 4:1 (5 mL) were added. The cell was equipped with graphite plate (1.5 cm × 1 cm × 0. 2 cm) as the anode and platinum plate (1 cm × 1 cm × 0.01 cm) as the cathode and connected to a DC regulated power supply. The reaction mixture was stirred and electrolyzed at a constant current of 10 mA at room temperature for 2 h. When the reaction was finished, the mixture was concentrated under reduced pressure. Purification by column chromatography on silica gel (eluent: petroleum ether) yielded product **3a** (60 mg, 57%) as yellow solid.

c)

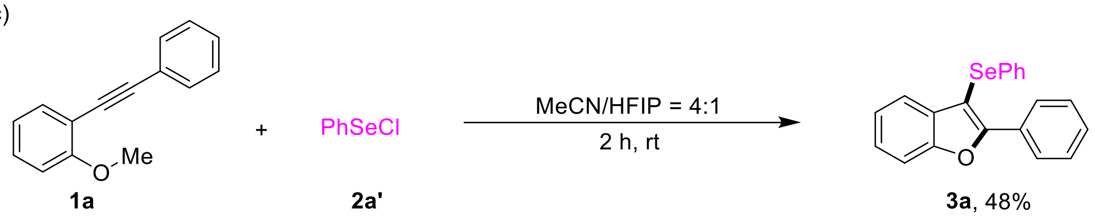

To a solution of compound **1a** (0.3 mmol, 62 mg) in MeCN/HFIP= 4:1 (5.0 mL) at room temperature, was added **2a'** (0.36 mmol, 69 mg). The solution was stirred for 2 hours at room temperature without electric current. The crude compound was purified by silica gel chromatography (eluent: petroleum ether) to give a yellow solid **3a** (50 mg, 48%).

d)

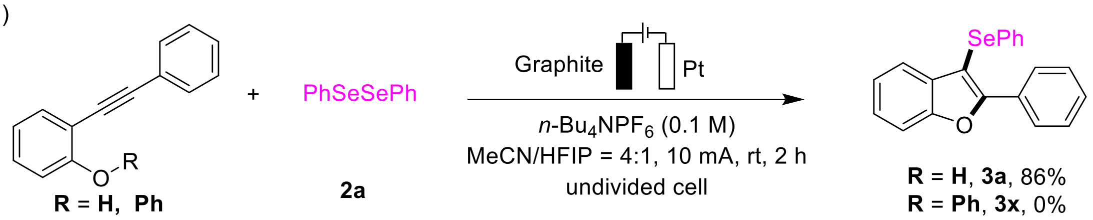

In an undivided cell (10 mL) equipped with a stirring bar, a mixture of 2-(phenylethynyl)phenol **1** (0.3 mmol), **2a** (0.36 mmol), *n*-Bu_4NPF_6 (0.5 mmol) and MeCN/HFIP = 4:1 (5 mL) were added. The cell was equipped with graphite plate (1.5 cm × 1 cm × 0. 2 cm) as the anode and platinum plate (1 cm × 1 cm × 0.01 cm) as the cathode and connected to a DC regulated power supply. The reaction mixture was stirred and electrolyzed at a constant current of 10 mA at room temperature for 2 h. When the reaction was finished, the mixture was concentrated under reduced pressure. Purification by column chromatography on silica gel (eluent: petroleum ether) yielded product **3a** (90 mg, 86%) as yellow solid. In addition, when the substituent was phenyl, the product of **3x** was not produced under standard conditions.

e)

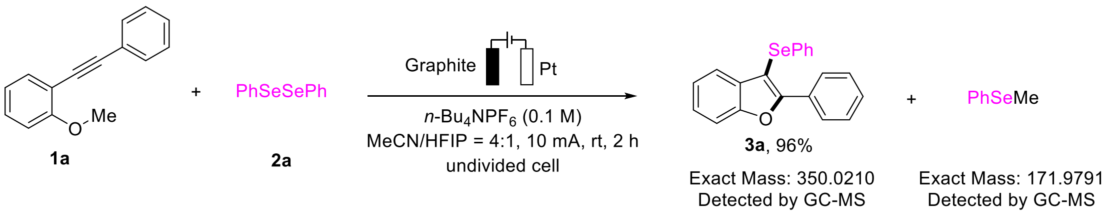

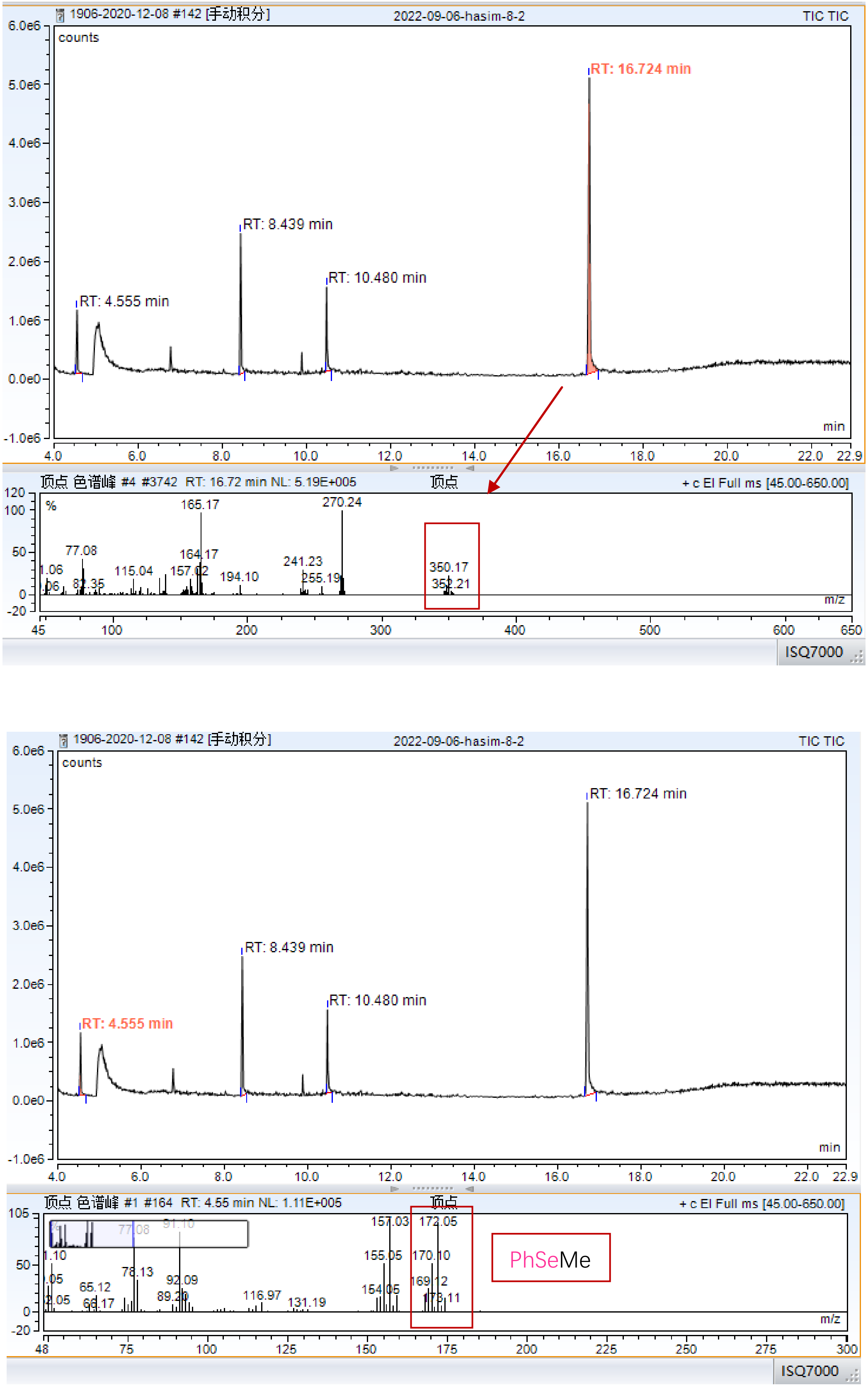

Figure S2. GC-MS detection of PhSeMe

Cyclic Voltammetry Experiment

Cyclic voltammetry experiments were carried out on an IGS 1230 electrochemical work station (Ingsens instruments, Guangzhou). 0.1 M electrolyte was dissolved in acetonitrile. Working electrode: glassy carbon, counter electrode: Pt, reference electrode: Ag/AgCl (3 M KCl). Scan rate: 100 mV/s.

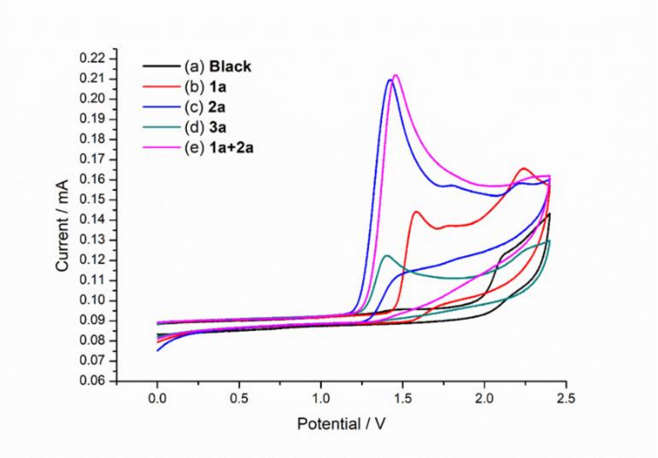

Figure S3. Cyclic voltammetry studies. Conditions: a 0.1 M *n*-Bu_4NPF_6 solution in MeCN/HFIP = 4:1 at room temperature; a glassy carbon working electrode, Ag/AgCl (3 M KCl) reference electrode, and a graphite counter electrode, respectively. Scan rate: 100mV/s. (a) Black line: background; (b) Red line: **1a** (1mM); (c) Blue line: **2a** (1mM); (d) Green line: **3a** (1mM); (e) Pink line: **1a** (1 mM) + **2a** (1 mM).

Crystallographic Details

Table S2. Crystal data and structure refinement for **3l**.

Identification code	**2201985**
Empirical formula	$C_{28}H_{18}OSe$
Formula weight	449.38
Temperature/K	296.15
Crystal system	monoclinic
Space group	$P2_1/c$
a/Å	9.9986(17)
b/Å	16.723(3)
c/Å	13.239(2)
α/°	90
β/°	111.216(3)
γ/°	90
Volume/Å^3	2063.6(6)
Z	4
ρ_{calc}g/cm^3	1.446
μ/mm^{-1}	1.837
F(000)	912.0
Crystal size/mm^3	0.14 × 0.12 × 0.1
Radiation	MoKα (λ = 0.71073)
2Θ range for data collection/°	5.886 to 50.23
Index ranges	$-11 \leq h \leq 10$, $-19 \leq k \leq 16$, $-13 \leq l \leq 15$
Reflections collected	23052
Independent reflections	3630 [R_{int} = 0.0957, R_{sigma} = 0.0884]
Data/restraints/parameters	3630/0/271
Goodness-of-fit on F^2	1.015
Final R indexes [I>=2σ (I)]	R_1 = 0.0484, wR_2 = 0.0868
Final R indexes [all data]	R_1 = 0.1240, wR_2 = 0.1120
Largest diff. peak/hole / e Å^{-3}	0.28/-0.43

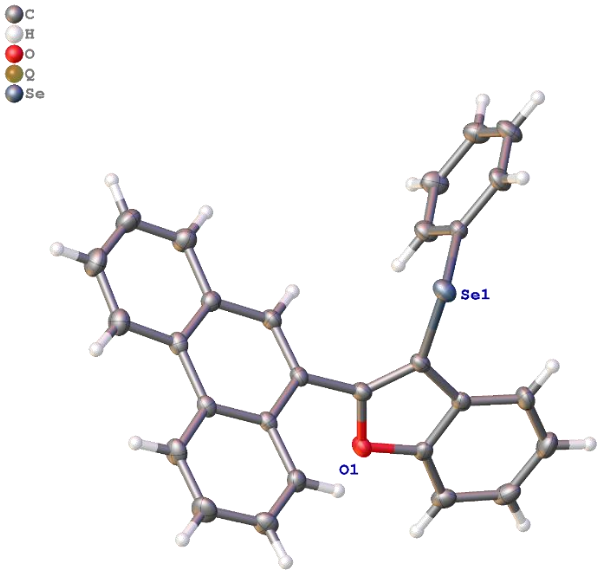

Figure S4. X-ray structure of **3l**.

Fractional Atomic Coordinates (×104) and Equivalent Isotropic Displacement Parameters (Å2×103) for 3l. Ueq is defined as 1/3 of the trace of the orthogonalised UIJ tensor.

Atom	*x*	*y*	*z*	U(eq)
Se1	3969.9(5)	6083.0(3)	5592.0(4)	55.6(2)
O1	8247(3)	5544.4(16)	6846(2)	48.8(7)
C1	7350(4)	8217(2)	5826(3)	46.1(11)
C2	6912(5)	8934(3)	6169(4)	65.3(13)
C3	7140(6)	9651(3)	5788(4)	73.9(15)
C4	7829(6)	9687(3)	5053(4)	73.9(15)
C5	8292(5)	9008(3)	4720(4)	64.7(13)
C6	8061(4)	8245(3)	5089(3)	46.0(11)
C7	8492(4)	7500(3)	4732(3)	43.2(10)
C8	8131(4)	6763(2)	5093(3)	41.3(10)
C9	8467(4)	6048(3)	4679(3)	49.8(11)
C10	9177(5)	6055(3)	3977(3)	60.0(13)
C11	9594(5)	6775(3)	3660(4)	69.7(14)
C12	9240(5)	7476(3)	4027(3)	60.5(13)
C13	7408(4)	6773(2)	5860(3)	41.9(10)
C14	7056(4)	7465(3)	6200(3)	48.6(11)
C15	7071(5)	6022(2)	6296(3)	45.2(11)
C16	5842(4)	5688(2)	6308(3)	44.8(11)
C17	6247(5)	4948(2)	6904(3)	44.4(11)
C18	5507(5)	4340(3)	7186(4)	60.6(13)
C19	6305(7)	3715(3)	7796(4)	74.4(15)

Atom	*x*	*y*	*z*	U(eq)
C20	7782(7)	3687(3)	8104(4)	70.2(15)
C21	8525(5)	4277(3)	7816(4)	57.6(13)
C22	7708(5)	4893(2)	7212(3)	44.8(11)
C23	3484(4)	6349(2)	6821(3)	43.8(10)
C24	2057(5)	6361(3)	6688(4)	60.1(13)
C25	1616(5)	6597(3)	7514(4)	69.1(14)
C26	2605(6)	6826(3)	8487(4)	64.6(13)
C27	4033(5)	6815(3)	8627(4)	65.3(14)
C28	4474(5)	6574(3)	7810(4)	57.1(12)

Anisotropic Displacement Parameters (Å2×103) for 3l. The Anisotropic displacement factor exponent takes the form: -2π2[h2a*2U11+2hka*b*U12+…].

Atom	U_{11}	U_{22}	U_{33}	U_{23}	U_{13}	U_{12}
Se1	43.9(3)	73.2(4)	50.7(3)	0.7(2)	18.3(2)	-1.4(3)
O1	43.2(19)	55.2(18)	53.8(18)	5.2(15)	24.3(16)	-3.5(16)
C1	46(3)	45(3)	46(3)	-2(2)	15(2)	-2(2)
C2	77(4)	62(3)	65(3)	-3(3)	35(3)	0(3)
C3	88(4)	50(3)	87(4)	-7(3)	35(4)	3(3)
C4	92(4)	54(3)	76(4)	10(3)	30(3)	-9(3)
C5	71(4)	63(3)	61(3)	8(3)	24(3)	-9(3)
C6	44(3)	57(3)	35(3)	6(2)	11(2)	-7(2)
C7	39(3)	57(3)	33(2)	1(2)	13(2)	-6(2)
C8	34(2)	54(3)	36(2)	0(2)	13(2)	-5(2)
C9	49(3)	52(3)	49(3)	-6(2)	19(2)	-11(2)
C10	65(3)	75(3)	48(3)	-9(3)	29(3)	0(3)
C11	81(4)	85(4)	60(3)	-5(3)	46(3)	-6(3)
C12	71(4)	72(3)	48(3)	2(3)	32(3)	-15(3)
C13	35(3)	47(3)	45(3)	4(2)	15(2)	-3(2)
C14	47(3)	54(3)	52(3)	1(2)	27(2)	-3(2)
C15	43(3)	50(3)	48(3)	2(2)	23(2)	3(2)
C16	38(3)	54(3)	47(3)	-5(2)	21(2)	-2(2)
C17	51(3)	46(3)	47(3)	-7(2)	30(2)	-3(2)
C18	58(3)	60(3)	77(3)	-1(3)	40(3)	-12(3)
C19	98(5)	45(3)	93(4)	1(3)	51(4)	-9(3)
C20	102(5)	50(3)	73(4)	8(3)	49(4)	18(3)
C21	66(3)	58(3)	57(3)	-2(3)	31(3)	12(3)
C22	55(3)	40(3)	49(3)	-6(2)	31(3)	-2(2)
C23	37(3)	43(2)	54(3)	-1(2)	20(2)	-2(2)
C24	37(3)	75(3)	64(3)	-11(3)	14(2)	-8(2)
C25	45(3)	94(4)	76(4)	-6(3)	31(3)	1(3)
C26	61(4)	77(3)	61(4)	-2(3)	29(3)	12(3)
C27	54(4)	84(4)	49(3)	-15(3)	8(3)	3(3)

Atom	U_{11}	U_{22}	U_{33}	U_{23}	U_{13}	U_{12}
C28	39(3)	70(3)	58(3)	-3(3)	13(3)	2(2)

Bond Lengths for 3l.

Atom	Atom	Length/Å	Atom	Atom	Length/Å
Se1	C16	1.885(4)	C11	C12	1.366(6)
Se1	C23	1.911(4)	C13	C14	1.334(5)
O1	C15	1.390(5)	C13	C15	1.471(5)
O1	C22	1.378(4)	C15	C16	1.356(5)
C1	C2	1.407(6)	C16	C17	1.445(6)
C1	C6	1.400(5)	C17	C18	1.384(5)
C1	C14	1.421(5)	C17	C22	1.371(6)
C2	C3	1.352(6)	C18	C19	1.384(7)
C3	C4	1.383(6)	C19	C20	1.383(7)
C4	C5	1.359(6)	C20	C21	1.370(6)
C5	C6	1.415(6)	C21	C22	1.377(6)
C6	C7	1.453(5)	C23	C24	1.374(6)
C7	C8	1.415(5)	C23	C28	1.378(6)
C7	C12	1.391(5)	C24	C25	1.376(6)
C8	C9	1.406(5)	C25	C26	1.365(6)
C8	C13	1.443(5)	C26	C27	1.372(6)
C9	C10	1.359(5)	C27	C28	1.367(6)
C10	C11	1.387(6)			

Bond Angles for 3l.

Atom	Atom	Atom	Angle/°	Atom	Atom	Atom	Angle/°
C16	Se1	C23	99.52(17)	C13	C14	C1	122.4(4)
C22	O1	C15	105.9(3)	O1	C15	C13	115.2(3)
C2	C1	C14	120.9(4)	C16	C15	O1	110.8(3)
C6	C1	C2	119.4(4)	C16	C15	C13	134.0(4)
C6	C1	C14	119.6(4)	C15	C16	Se1	126.1(3)
C3	C2	C1	121.5(4)	C15	C16	C17	106.5(4)
C2	C3	C4	119.7(5)	C17	C16	Se1	127.3(3)
C5	C4	C3	120.4(5)	C18	C17	C16	134.8(4)
C4	C5	C6	121.7(5)	C22	C17	C16	106.1(4)
C1	C6	C5	117.3(4)	C22	C17	C18	119.2(4)
C1	C6	C7	118.9(4)	C17	C18	C19	117.4(5)
C5	C6	C7	123.8(4)	C20	C19	C18	121.7(5)
C8	C7	C6	119.7(4)	C21	C20	C19	121.5(5)
C12	C7	C6	122.5(4)	C20	C21	C22	115.7(5)
C12	C7	C8	117.8(4)	O1	C22	C21	124.8(4)
C7	C8	C13	118.8(4)	C17	C22	O1	110.7(4)
C9	C8	C7	118.8(4)	C17	C22	C21	124.5(4)

Atom	Atom	Atom	Angle/˚	Atom	Atom	Atom	Angle/˚
C9	C8	C13	122.4(4)	C24	C23	Se1	117.8(3)
C10	C9	C8	121.2(4)	C24	C23	C28	118.3(4)
C9	C10	C11	120.2(4)	C28	C23	Se1	123.8(3)
C12	C11	C10	119.5(4)	C25	C24	C23	121.2(4)
C11	C12	C7	122.3(4)	C26	C25	C24	120.0(5)
C8	C13	C15	120.7(4)	C25	C26	C27	119.1(4)
C14	C13	C8	120.5(4)	C28	C27	C26	121.0(4)
C14	C13	C15	118.8(4)	C27	C28	C23	120.4(4)

Torsion Angles for 3l.

A	B	C	D	Angle/˚	A	B	C	D	Angle/˚
Se1	C16	C17	C18	-3.4(7)	C12	C7	C8	C13	177.4(4)
Se1	C16	C17	C22	176.0(3)	C13	C8	C9	C10	-178.5(4)
Se1	C23	C24	C25	-175.7(4)	C13	C15	C16	Se1	6.1(7)
Se1	C23	C28	C27	175.0(3)	C13	C15	C16	C17	-178.0(4)
O1	C15	C16	Se1	-175.8(3)	C14	C1	C2	C3	-177.6(4)
O1	C15	C16	C17	0.1(4)	C14	C1	C6	C5	178.3(4)
C1	C2	C3	C4	-0.6(8)	C14	C1	C6	C7	-0.5(6)
C1	C6	C7	C8	2.9(6)	C14	C13	C15	O1	-119.7(4)
C1	C6	C7	C12	-178.0(4)	C14	C13	C15	C16	58.3(6)
C2	C1	C6	C5	-0.5(6)	C15	O1	C22	C17	0.4(4)
C2	C1	C6	C7	-179.3(4)	C15	O1	C22	C21	-178.9(4)
C2	C1	C14	C13	177.4(4)	C15	C13	C14	C1	179.6(4)
C2	C3	C4	C5	-0.7(8)	C15	C16	C17	C18	-179.2(5)
C3	C4	C5	C6	1.4(8)	C15	C16	C17	C22	0.2(4)
C4	C5	C6	C1	-0.8(7)	C16	C17	C18	C19	-179.0(4)
C4	C5	C6	C7	177.9(4)	C16	C17	C22	O1	-0.3(4)
C5	C6	C7	C8	-175.9(4)	C16	C17	C22	C21	178.9(4)
C5	C6	C7	C12	3.2(7)	C17	C18	C19	C20	-0.9(7)
C6	C1	C2	C3	1.2(7)	C18	C17	C22	O1	179.1(3)
C6	C1	C14	C13	-1.3(6)	C18	C17	C22	C21	-1.6(6)
C6	C7	C8	C9	175.5(4)	C18	C19	C20	C21	-0.1(8)
C6	C7	C8	C13	-3.4(6)	C19	C20	C21	C22	0.3(7)
C6	C7	C12	C11	-177.3(4)	C20	C21	C22	O1	179.7(4)
C7	C8	C9	C10	2.6(6)	C20	C21	C22	C17	0.6(6)
C7	C8	C13	C14	1.7(6)	C22	O1	C15	C13	178.2(3)
C7	C8	C13	C15	-177.2(4)	C22	O1	C15	C16	-0.3(4)
C8	C7	C12	C11	1.8(7)	C22	C17	C18	C19	1.7(6)
C8	C9	C10	C11	0.4(7)	C23	Se1	C16	C15	-114.2(4)
C8	C13	C14	C1	0.7(6)	C23	Se1	C16	C17	70.7(4)
C8	C13	C15	O1	59.1(5)	C23	C24	C25	C26	0.1(7)
C8	C13	C15	C16	-122.8(5)	C24	C23	C28	C27	-0.9(6)
C9	C8	C13	C14	-177.2(4)	C24	C25	C26	C27	-0.1(7)

A	B	C	D	Angle/°	A	B	C	D	Angle/°
C9	C8	C13	C15	3.9(6)	C25	C26	C27	C28	-0.4(7)
C9	C10	C11	C12	-2.4(7)	C26	C27	C28	C23	0.9(7)
C10	C11	C12	C7	1.2(8)	C28	C23	C24	C25	0.4(7)
C12	C7	C8	C9	-3.6(6)					

Hydrogen Atom Coordinates (Å×104) and Isotropic Displacement Parameters (Å2×103) for 3l.

Atom	*x*	*y*	*z*	U(eq)
H2	6456.27	8913.92	6668.19	78
H3	6834.88	10117.96	6020.29	89
H4	7976.91	10180.06	4784.33	89
H5	8772.02	9045.76	4237.51	78
H9	8199.09	5562.15	4889.64	60
H10	9383.85	5575.72	3707.67	72
H11	10110.44	6778.97	3199.89	84
H12	9507.95	7955.79	3798.59	73
H14	6600.87	7454.49	6700.72	58
H18	4513.49	4352	6972.87	73
H19	5835.99	3303.22	8004.87	89
H20	8281.3	3257.28	8515.03	84
H21	9517.44	4262.28	8015.38	69
H24	1374.92	6206.46	6027.01	72
H25	645.02	6600.47	7408.44	83
H26	2314.9	6986.68	9048.29	77
H27	4711.68	6972.87	9287.07	78
H28	5447.39	6563.56	7922.73	68

Crystal structure determination of 3l

Crystal Data for $C_{28}H_{18}OSe$ (M =449.38 g/mol): monoclinic, space group $P2_1/c$ (no. 14), a = 9.9986(17) Å, b = 16.723(3) Å, c = 13.239(2) Å, β = 111.216(3)°, V = 2063.6(6) Å^3, Z = 4, T = 296.15 K, μ(MoKα) = 1.837 mm^{-1}, $Dcalc$ = 1.446 g/cm^3, 23052 reflections measured (5.886° ≤ 2Θ ≤ 50.23°), 3630 unique (R_{int} = 0.0957, R_{sigma} = 0.0884) which were used in all calculations. The final R_1 was 0.0484 (I > 2σ(I)) and wR_2 was 0.1120 (all data).

NMR Spectra

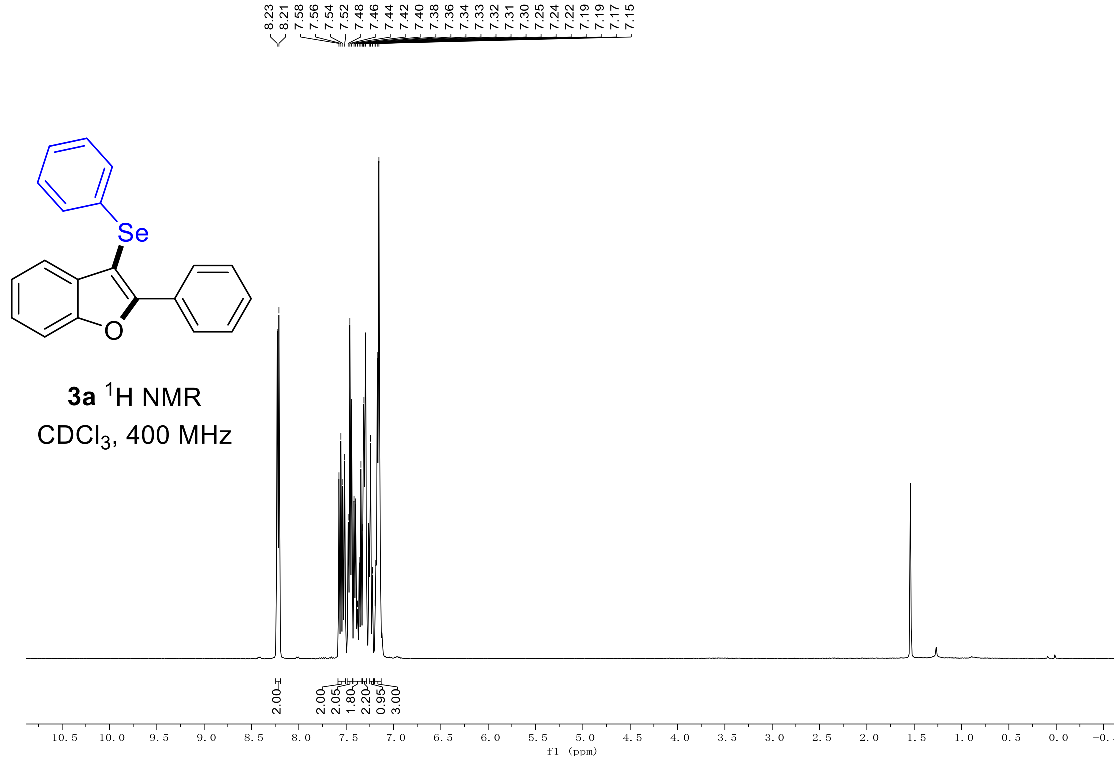
8.23
8.21
7.58
7.56
7.54
7.52
7.48
7.46
7.44
7.42
7.40
7.38
7.36
7.34
7.33
7.32
7.31
7.30
7.25
7.24
7.22
7.19
7.17
7.15
Se
O
3a 1H NMR
CDCl3, 400 MHz
2.00
2.00
2.05
1.80
2.20
0.95
3.00
f1 (ppm)

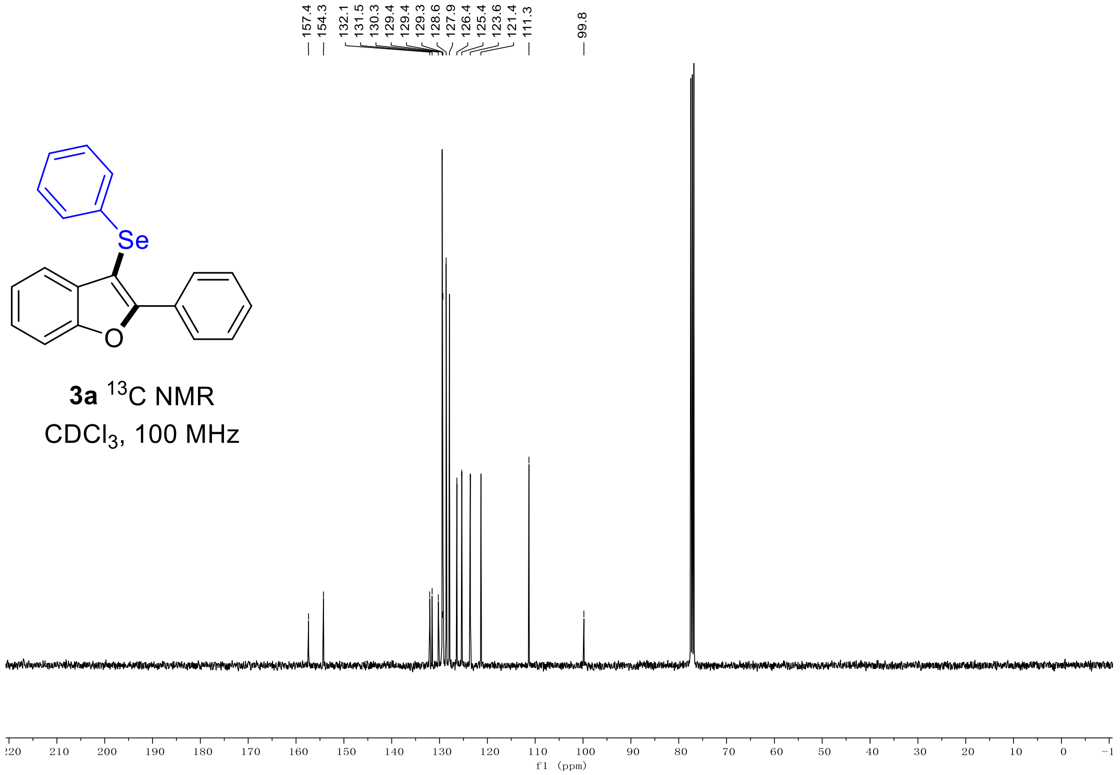
157.4
154.3
132.1
131.5
130.3
129.4
129.4
129.3
128.6
127.9
126.4
125.4
123.6
121.4
111.3
99.8
Se
O
3a 13C NMR
CDCl3, 100 MHz
f1 (ppm)

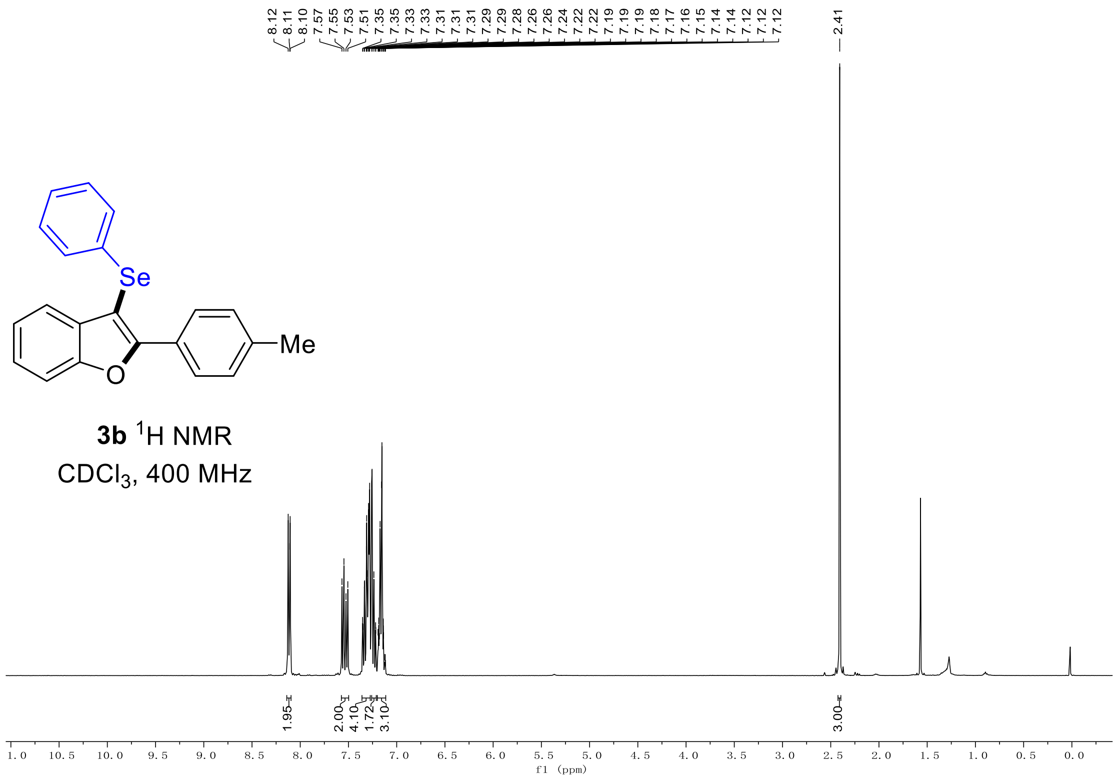
8.12
8.11
8.10
7.57
7.55
7.53
7.51
7.35
7.35
7.33
7.33
7.31
7.31
7.31
7.29
7.29
7.28
7.26
7.24
7.22
7.22
7.22
7.19
7.19
7.19
7.18
7.17
7.16
7.15
7.14
7.14
7.12
7.12
7.12
2.41
Se
Me
O
3b ^{1}H NMR
$CDCl_3$, 400 MHz
1.95
2.00
4.10
1.72
3.10
3.00
f1 (ppm)

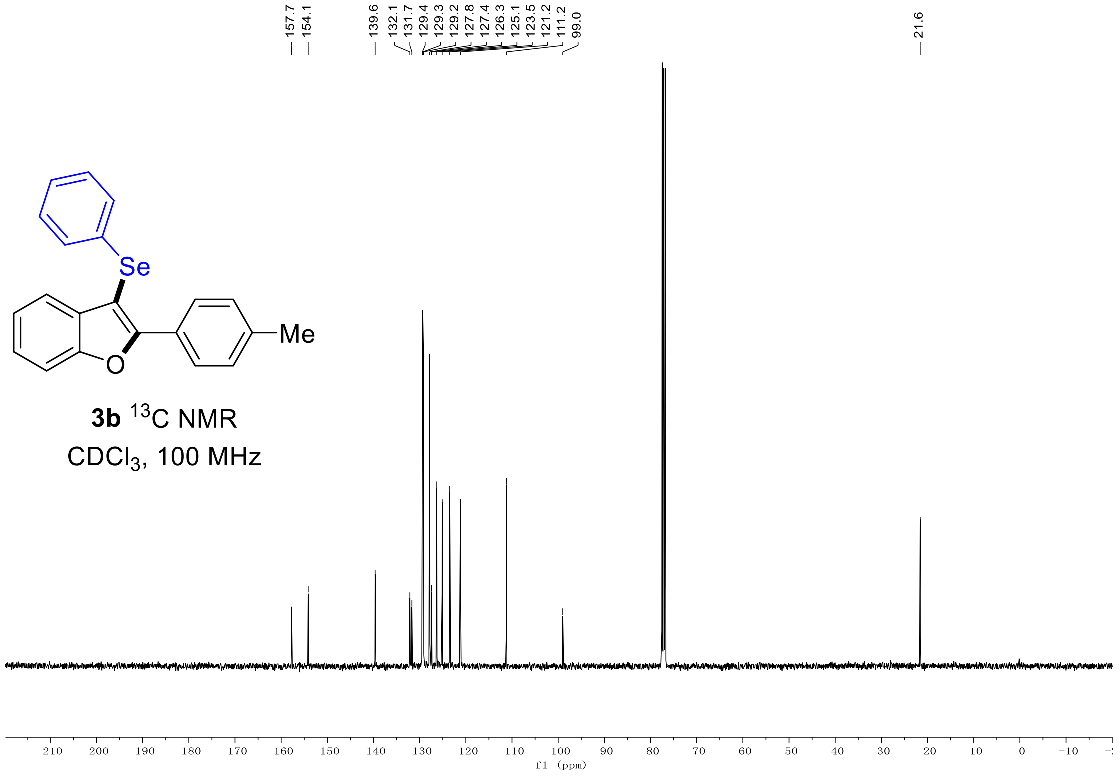
157.7
154.1
139.6
132.1
131.7
129.4
129.3
129.2
127.8
127.4
126.3
125.1
123.5
121.2
111.2
99.0
21.6
Se
Me
O
3b ^{13}C NMR
$CDCl_3$, 100 MHz
f1 (ppm)

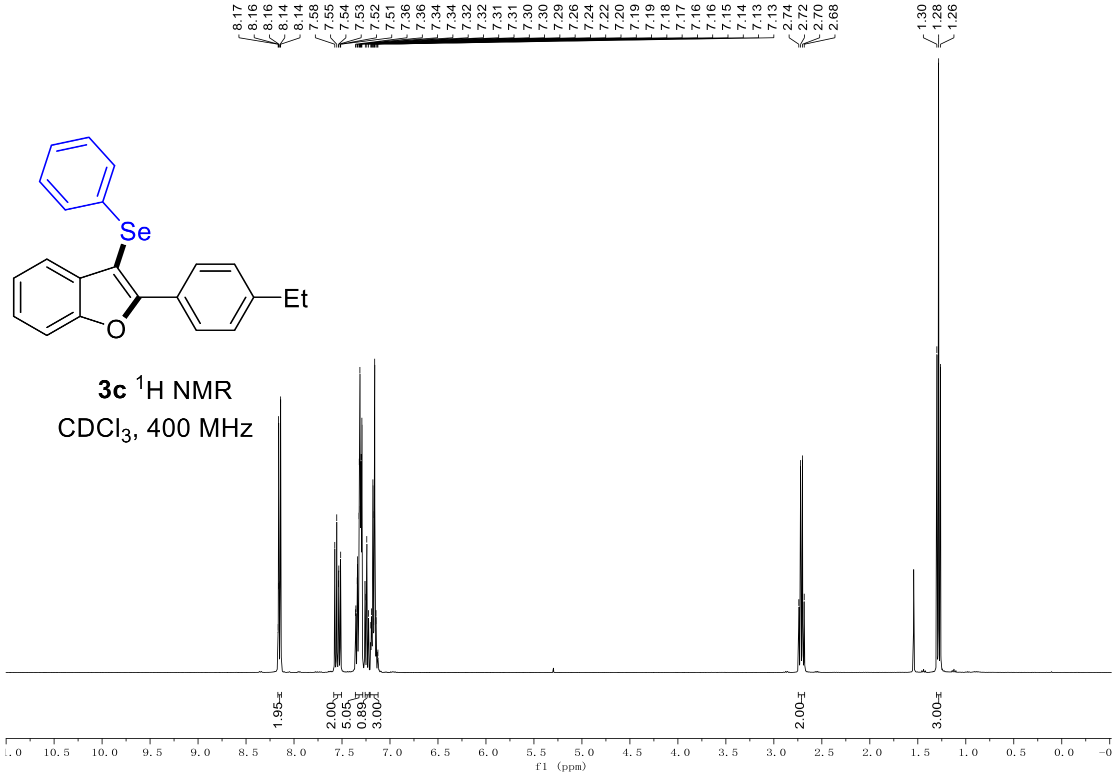
Se
O
Et
3c 1H NMR
CDCl3, 400 MHz
8.17 8.16 8.16 8.14 8.14 7.58 7.55 7.54 7.53 7.52 7.51 7.36 7.36 7.34 7.34 7.32 7.32 7.31 7.31 7.30 7.30 7.29 7.26 7.24 7.22 7.20 7.19 7.19 7.18 7.17 7.16 7.16 7.15 7.14 7.13 7.13 2.74 2.72 2.70 2.68 1.30 1.28 1.26
1.95 2.00 5.05 0.89 3.00 2.00 3.00
f1 (ppm)

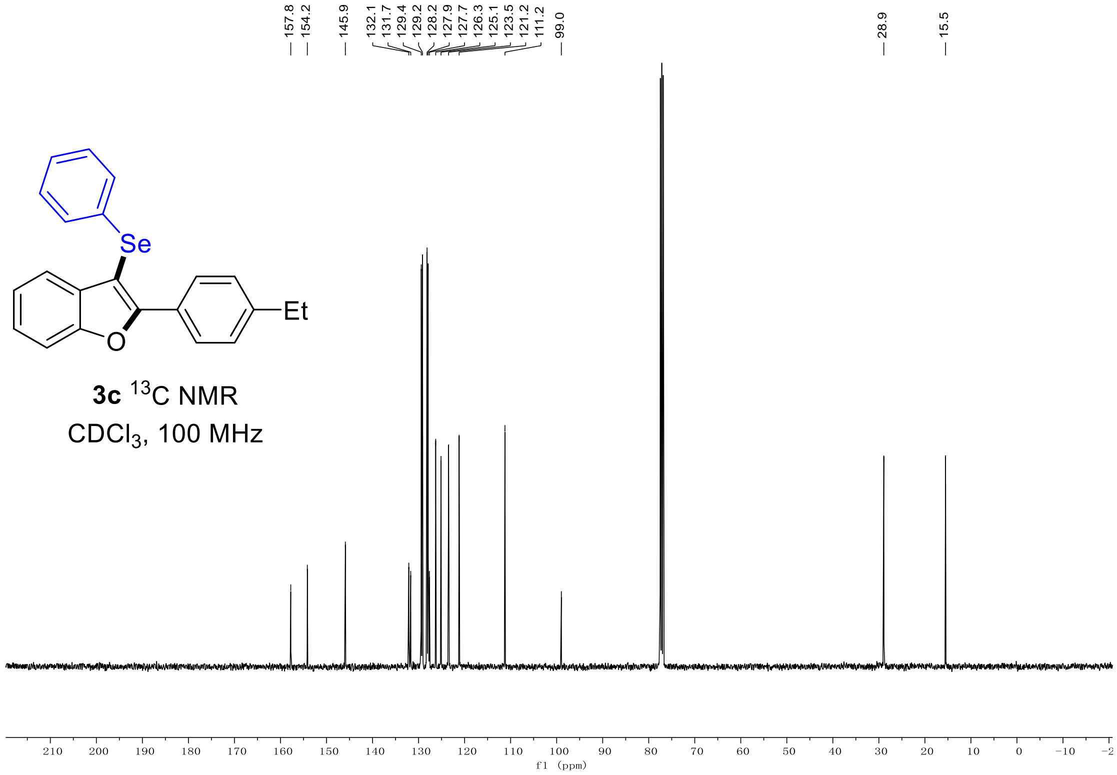
Se
O
Et
3c 13C NMR
CDCl3, 100 MHz
157.8 154.2 145.9 132.1 131.7 129.4 129.2 128.2 127.9 127.7 126.3 125.1 123.5 121.2 111.2 99.0 28.9 15.5
f1 (ppm)

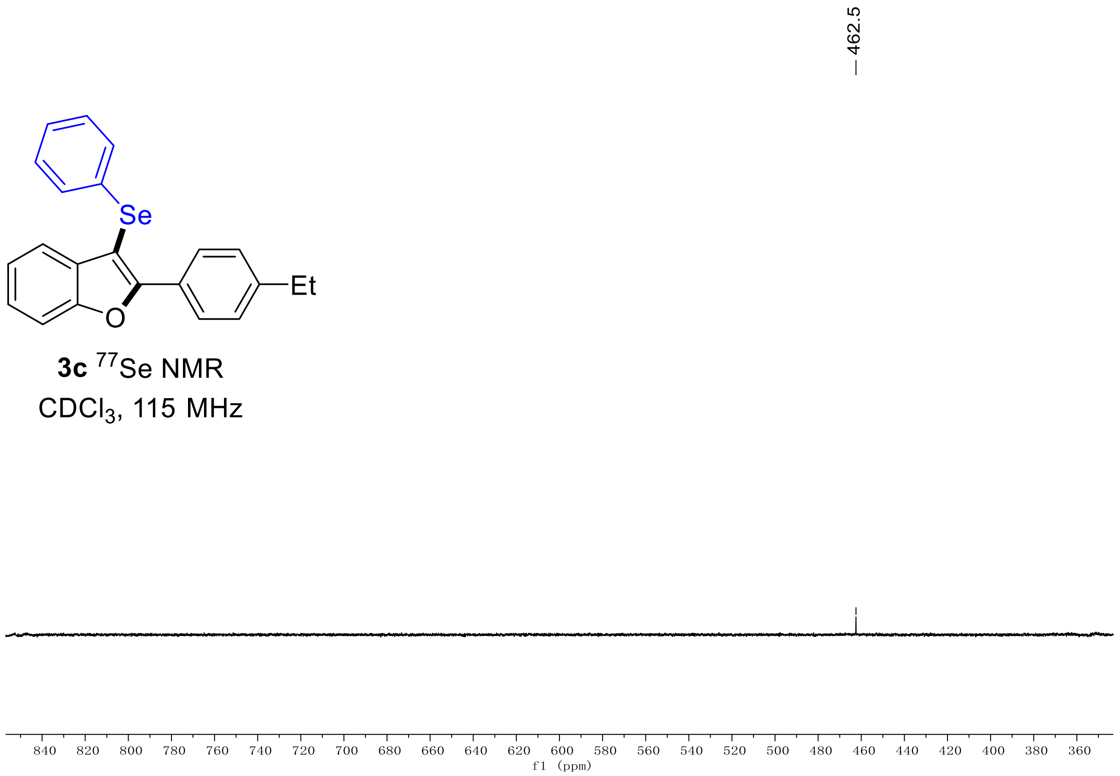
Se
Et
O
3c 77Se NMR
CDCl3, 115 MHz
462.5
f1 (ppm)

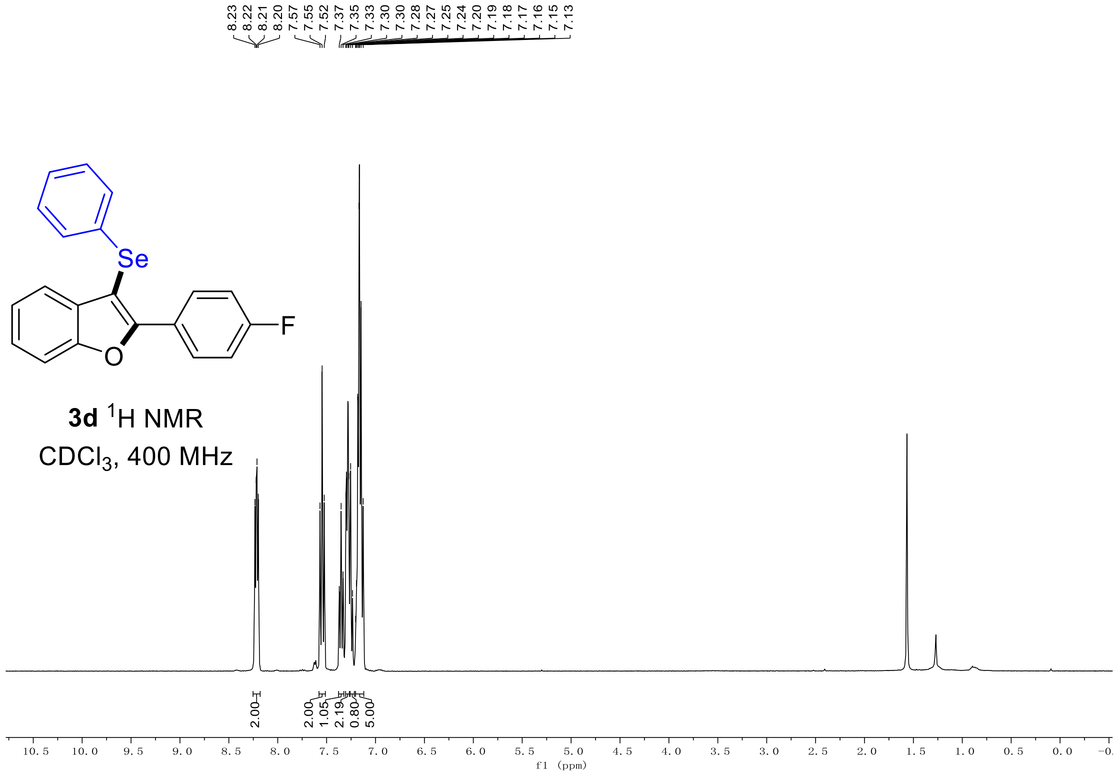
Se
F
O
3d 1H NMR
CDCl3, 400 MHz
f1 (ppm)

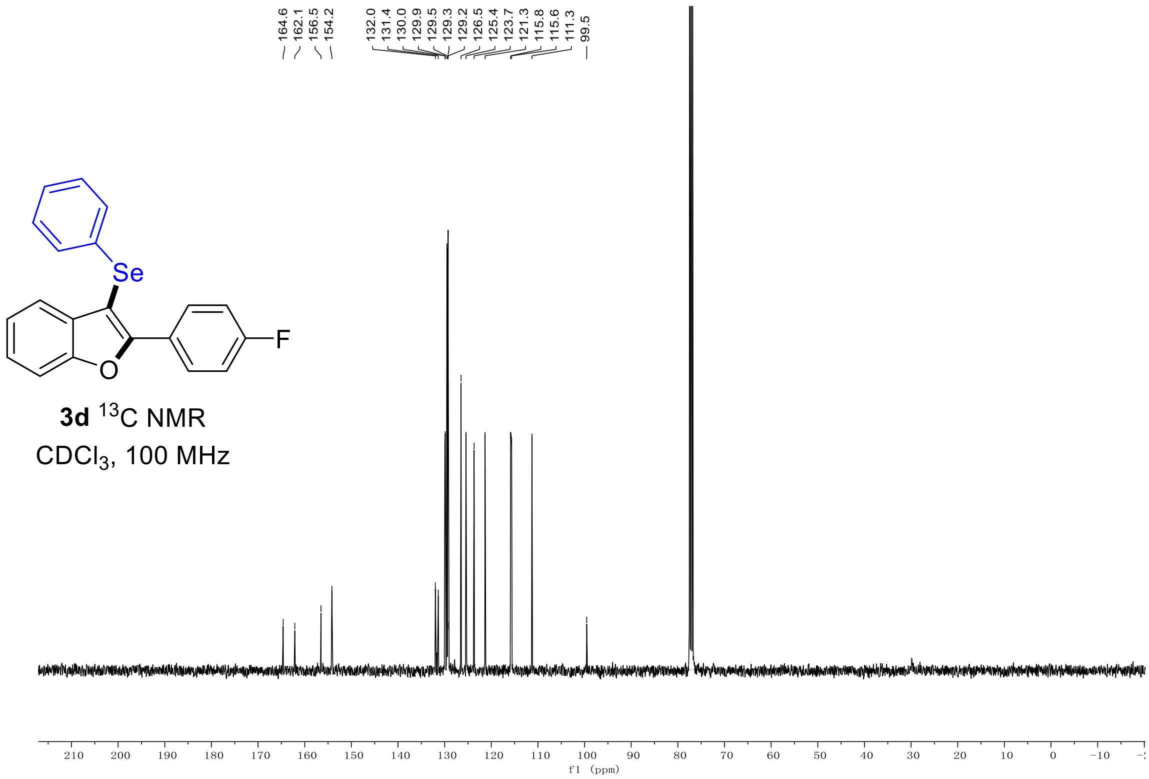
164.6
162.1
156.5
154.2
132.0
131.4
130.0
129.9
129.5
129.3
129.2
126.5
125.4
123.7
121.3
115.8
115.6
111.3
99.5
Se
F
O
3d ^{13}C NMR
$CDCl_3$, 100 MHz
f1 (ppm)

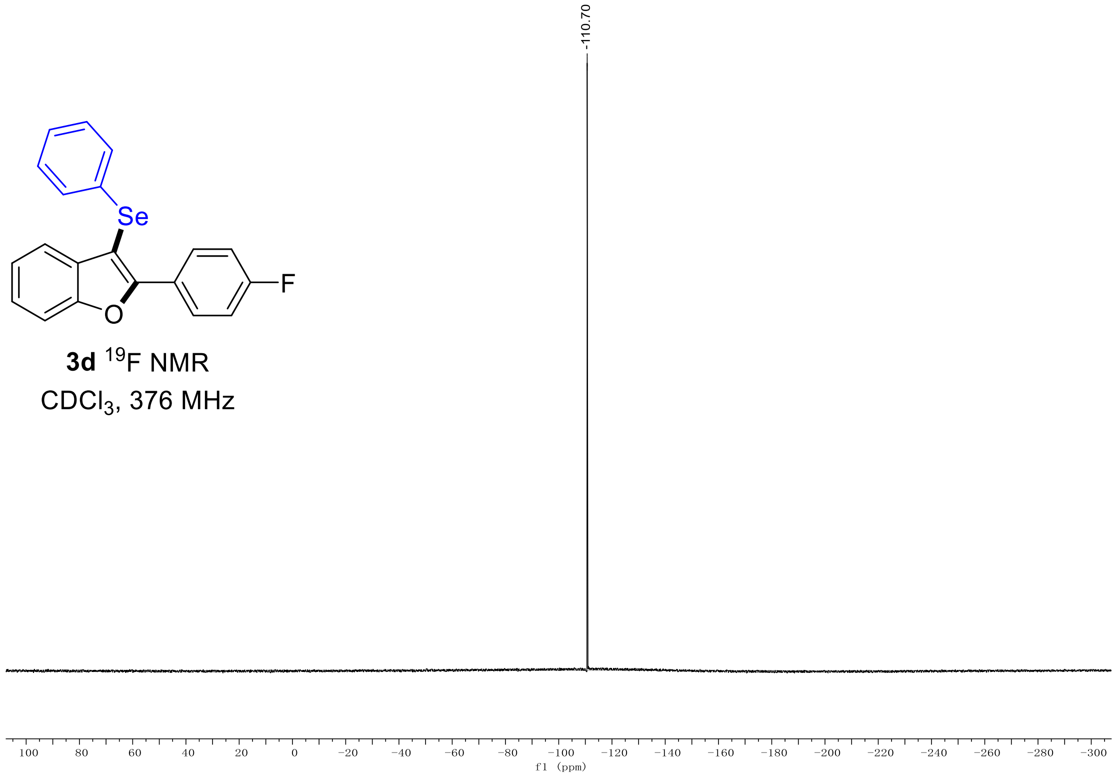
-110.70
Se
F
O
3d ^{19}F NMR
$CDCl_3$, 376 MHz
f1 (ppm)

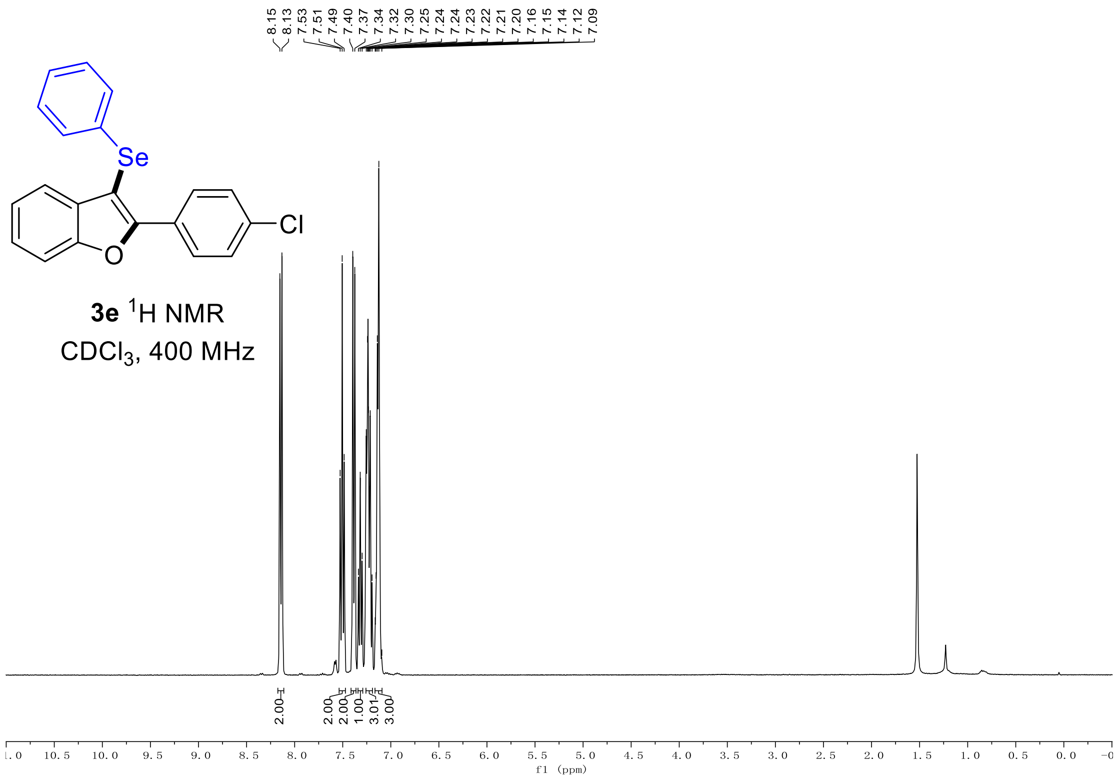
8.15
8.13
7.53
7.51
7.49
7.40
7.37
7.34
7.32
7.30
7.25
7.24
7.24
7.23
7.22
7.21
7.20
7.16
7.15
7.14
7.12
7.09
Se
Cl
O
3e 1H NMR
CDCl3, 400 MHz
2.00
2.00
2.00
1.00
3.01
3.00
f1 (ppm)

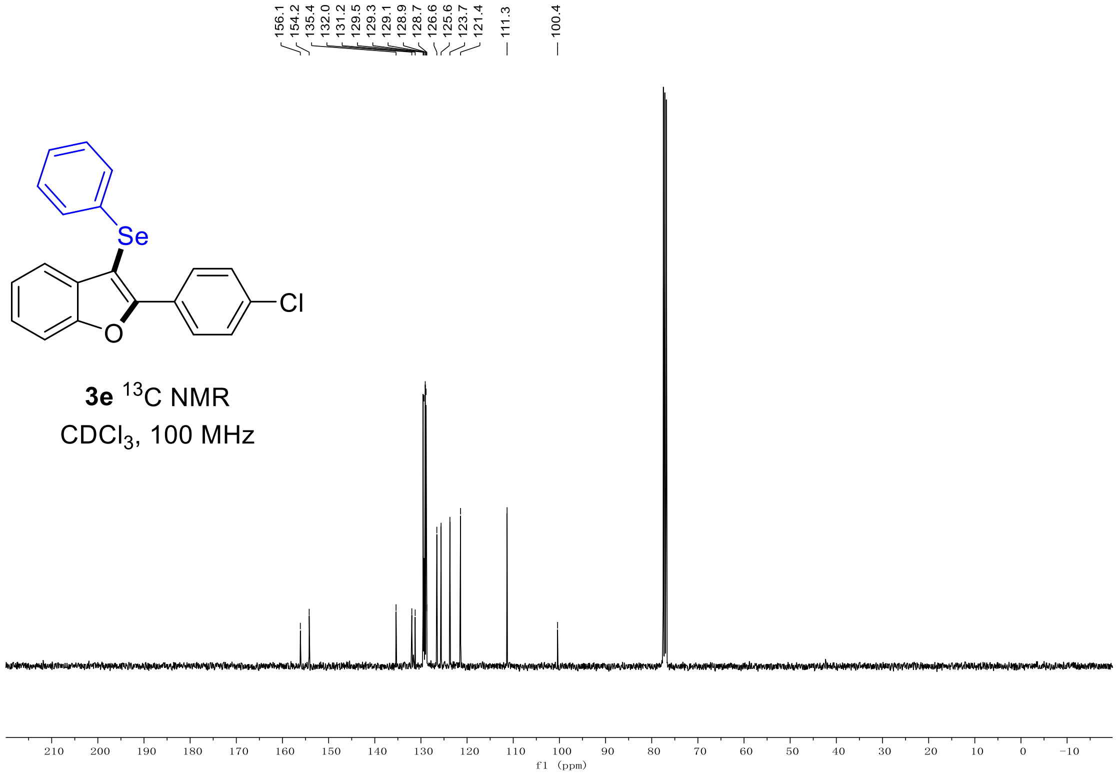
156.1
154.2
135.4
132.0
131.2
129.5
129.3
129.1
128.9
128.7
126.6
125.6
123.7
121.4
111.3
100.4
Se
Cl
O
3e 13C NMR
CDCl3, 100 MHz
f1 (ppm)

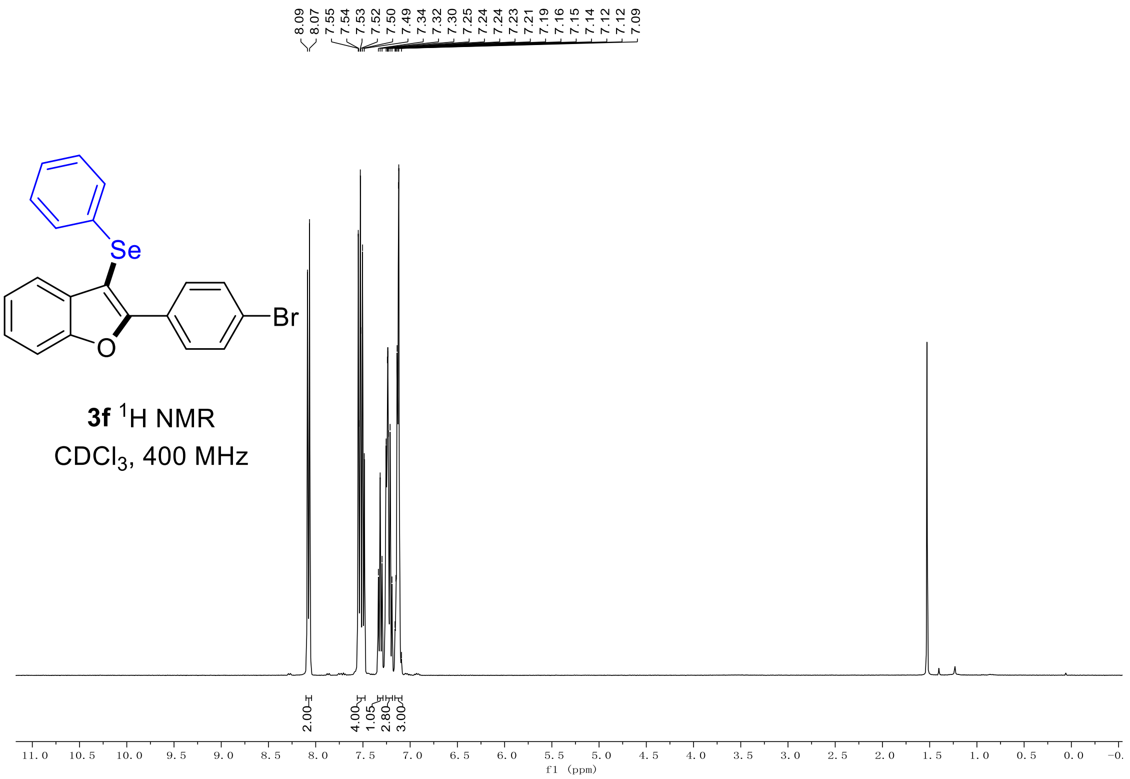
3f ^{1}H NMR
$CDCl_3$, 400 MHz
Se
Br
O
8.09
8.07
7.55
7.54
7.53
7.52
7.50
7.49
7.34
7.32
7.30
7.25
7.24
7.24
7.23
7.21
7.19
7.16
7.15
7.14
7.12
7.12
7.09
2.00
4.00
1.05
2.80
3.00
f1 (ppm)

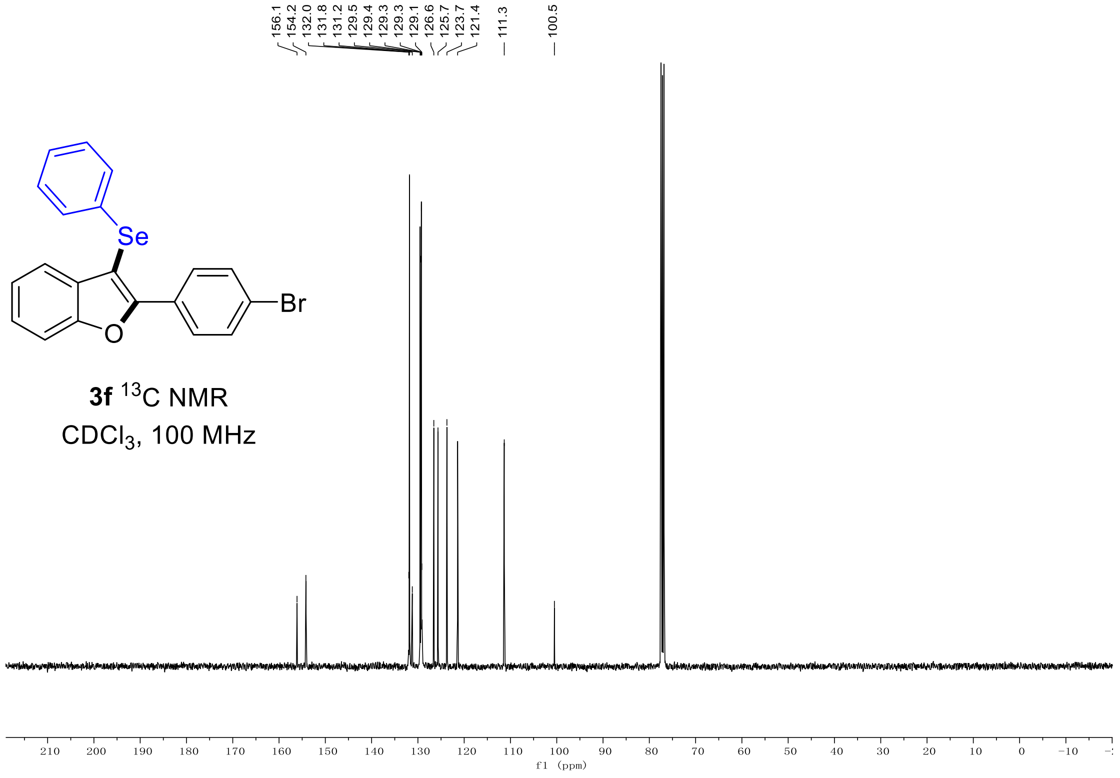
3f ^{13}C NMR
$CDCl_3$, 100 MHz
Se
Br
O
156.1
154.2
132.0
131.8
131.2
129.5
129.4
129.3
129.3
129.1
126.6
125.7
123.7
121.4
111.3
100.5
f1 (ppm)

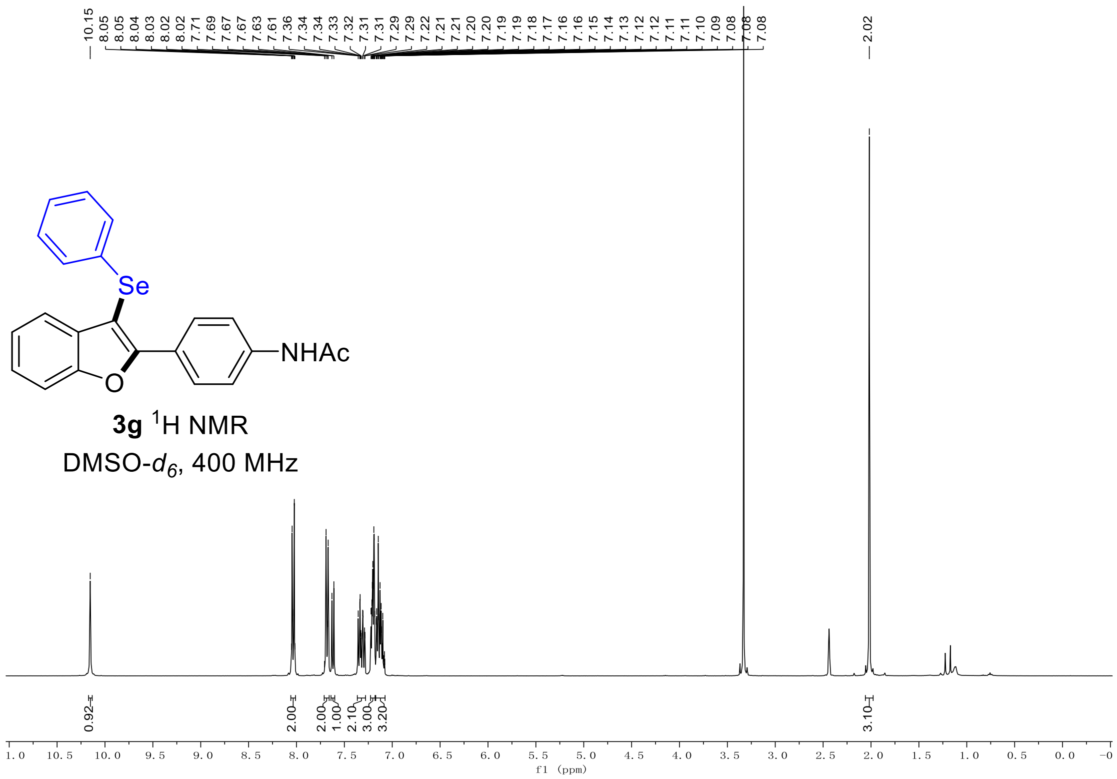
10.15
8.05
8.05
8.04
8.03
8.02
7.71
7.69
7.67
7.63
7.61
7.36
7.34
7.34
7.33
7.32
7.31
7.31
7.29
7.29
7.22
7.21
7.21
7.20
7.19
7.19
7.18
7.17
7.16
7.16
7.15
7.14
7.13
7.12
7.12
7.11
7.11
7.10
7.09
7.08
7.08
7.08
2.02
Se
NHAc
O
3g 1H NMR
DMSO-d6, 400 MHz
0.92
2.00
2.00
1.00
2.10
3.00
3.20
3.10
f1 (ppm)

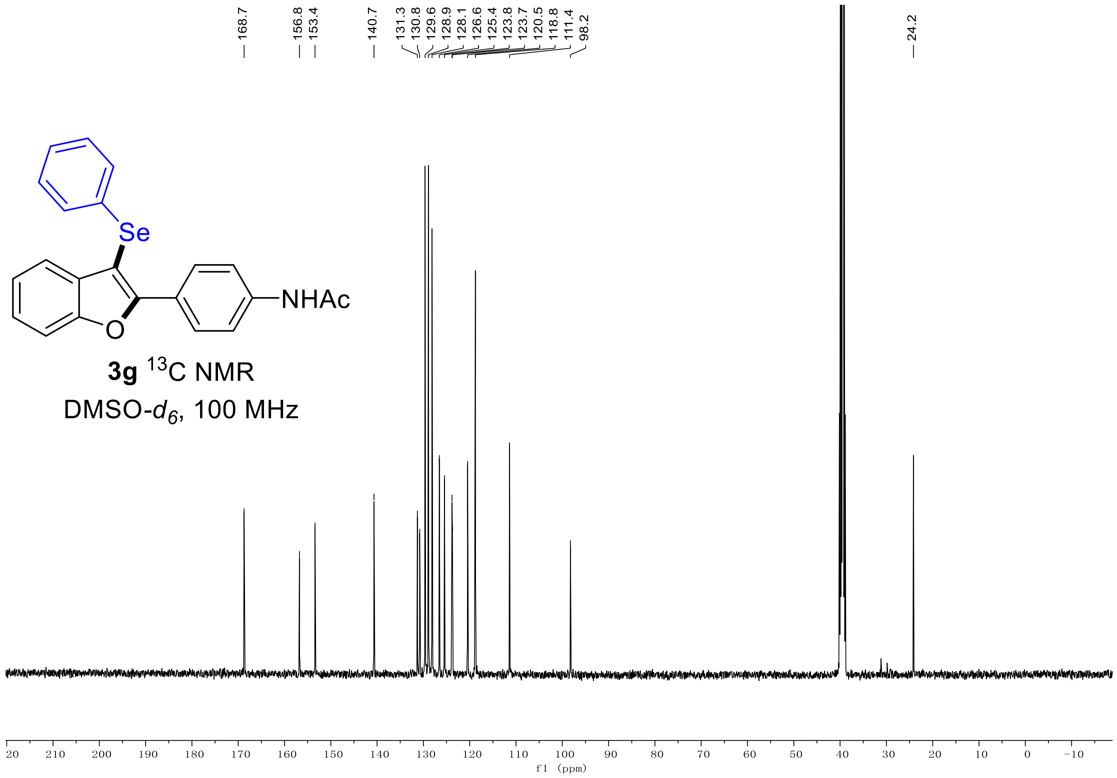
168.7
156.8
153.4
140.7
131.3
130.8
129.6
128.9
128.1
126.6
125.4
123.8
123.7
120.5
118.8
111.4
98.2
24.2
Se
NHAc
O
3g 13C NMR
DMSO-d6, 100 MHz
f1 (ppm)

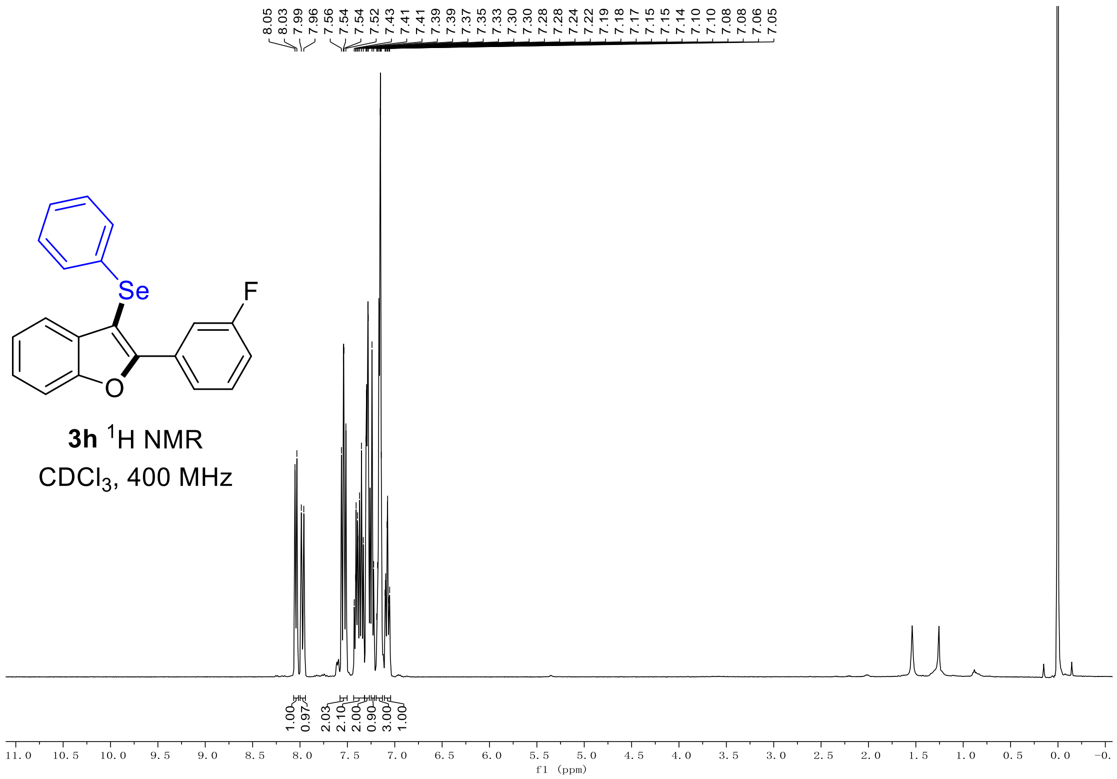
8.05
8.03
7.99
7.96
7.56
7.54
7.54
7.52
7.43
7.41
7.39
7.39
7.37
7.35
7.33
7.30
7.30
7.28
7.28
7.24
7.22
7.19
7.18
7.17
7.15
7.14
7.10
7.10
7.08
7.06
7.05
Se
F
O
3h ¹H NMR
CDCl3, 400 MHz
1.00
0.97
2.03
2.10
2.00
0.90
3.00
1.00
11.0 10.5 10.0 9.5 9.0 8.5 8.0 7.5 7.0 6.5 6.0 5.5 5.0 4.5 4.0 3.5 3.0 2.5 2.0 1.5 1.0 0.5 0.0 -0.
f1 (ppm)

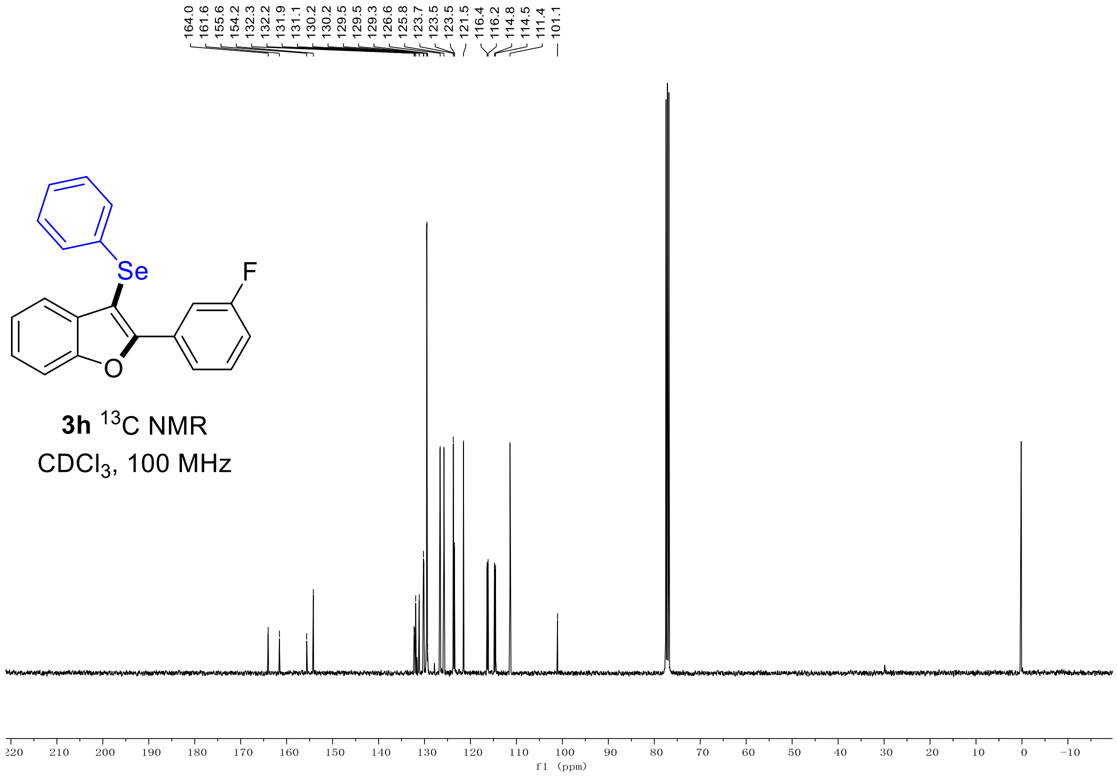
164.0
161.6
155.6
154.2
132.3
132.2
131.9
131.1
130.2
130.2
129.5
129.5
129.3
126.6
125.8
123.7
123.5
123.5
121.5
116.4
116.2
114.8
114.5
111.4
101.1
Se
F
O
3h ¹³C NMR
CDCl3, 100 MHz
220 210 200 190 180 170 160 150 140 130 120 110 100 90 80 70 60 50 40 30 20 10 0 -10
f1 (ppm)

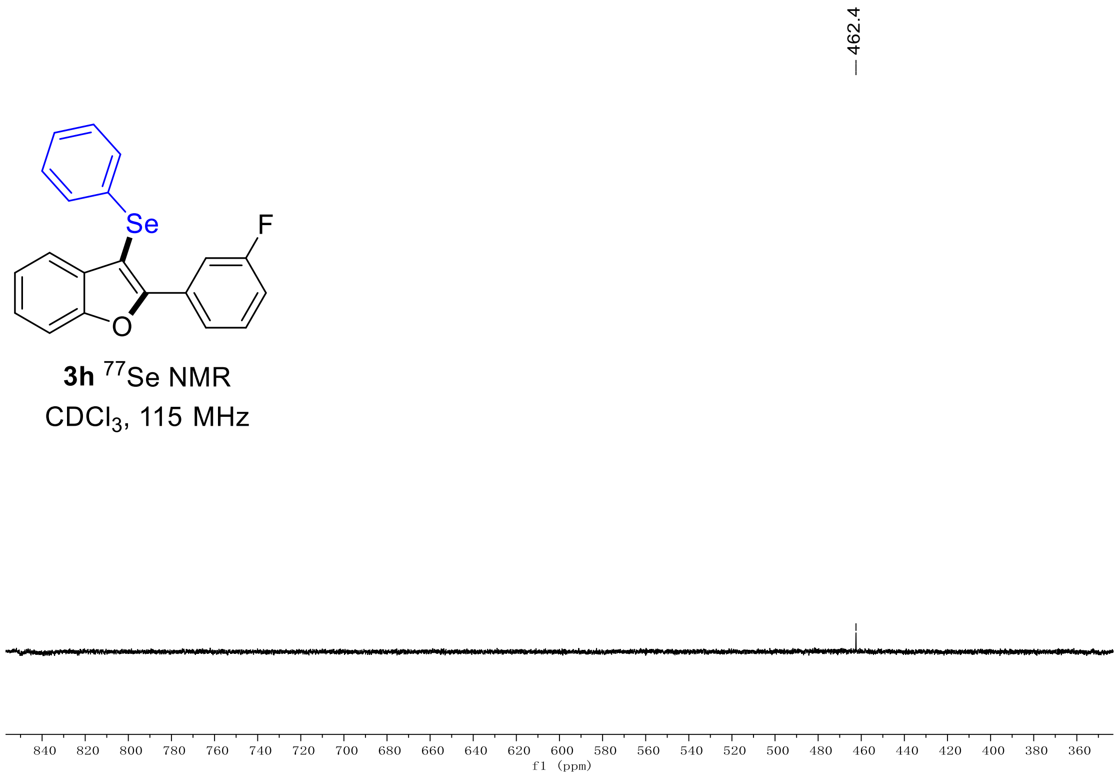
462.4
Se
F
O
3h 77Se NMR
CDCl3, 115 MHz
840 820 800 780 760 740 720 700 680 660 640 620 600 580 560 540 520 500 480 460 440 420 400 380 360
f1 (ppm)

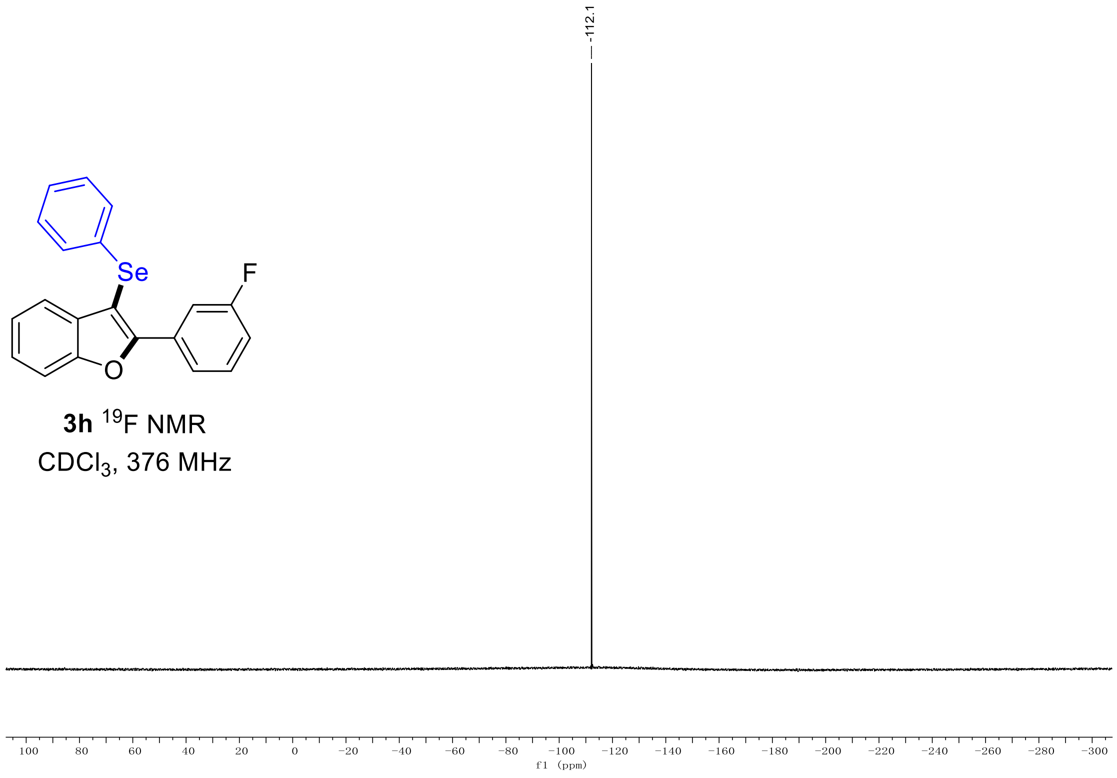
-112.1
Se
F
O
3h 19F NMR
CDCl3, 376 MHz
100 80 60 40 20 0 −20 −40 −60 −80 −100 −120 −140 −160 −180 −200 −220 −240 −260 −280 −300
f1 (ppm)

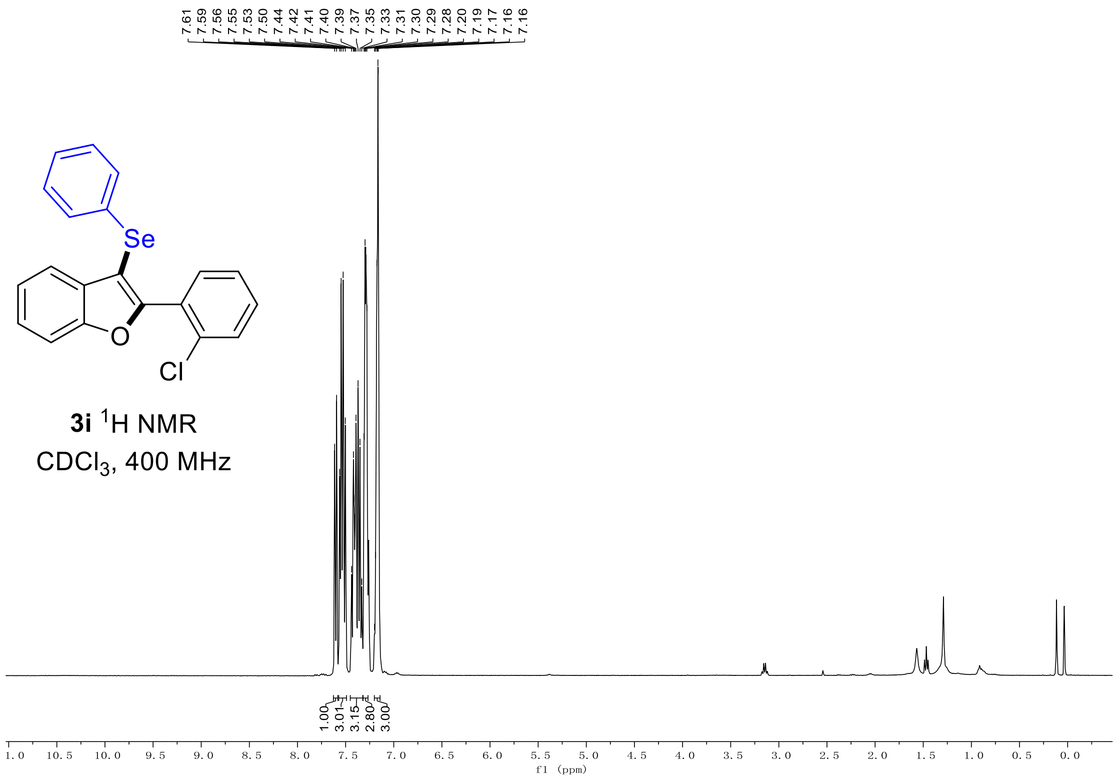
7.61 7.59 7.56 7.55 7.53 7.50 7.44 7.42 7.41 7.40 7.39 7.37 7.35 7.33 7.31 7.30 7.29 7.28 7.20 7.19 7.17 7.16 7.16
Se
O
Cl
3i 1H NMR
CDCl3, 400 MHz
1.00 3.01 3.15 2.80 3.00
f1 (ppm)

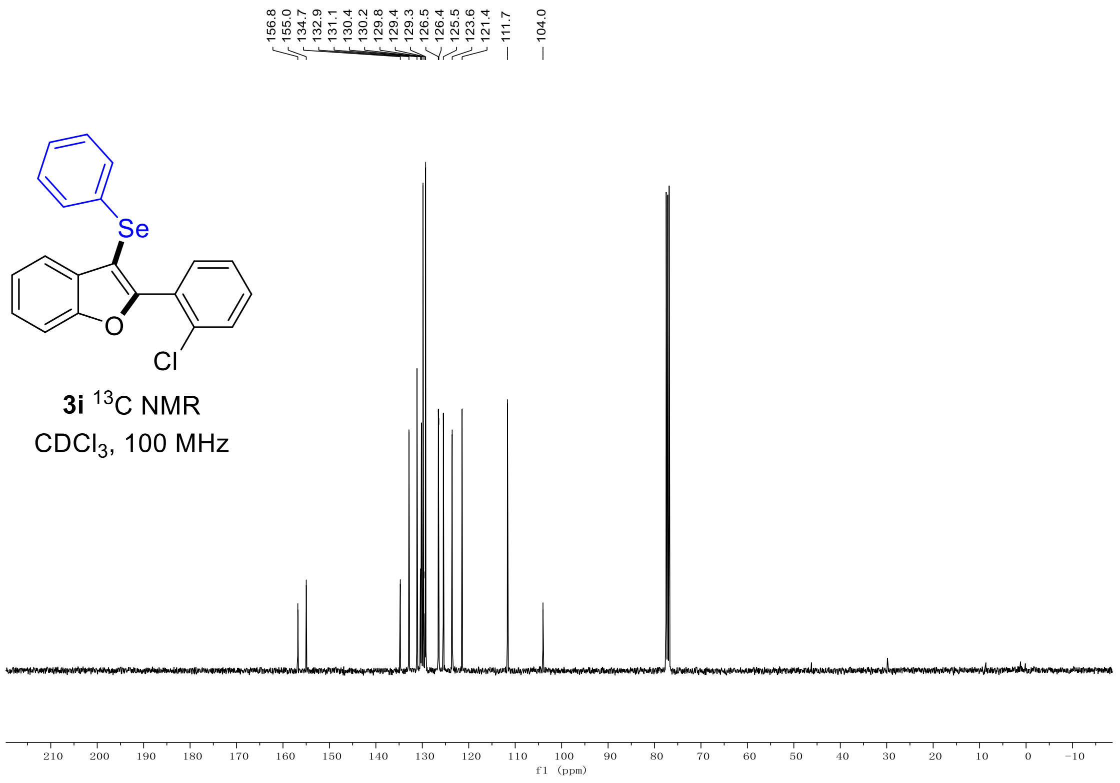
156.8 155.0 134.7 132.9 131.1 130.4 130.2 129.8 129.4 129.3 126.5 126.4 125.5 123.6 121.4 111.7 104.0
Se
O
Cl
3i 13C NMR
CDCl3, 100 MHz
f1 (ppm)

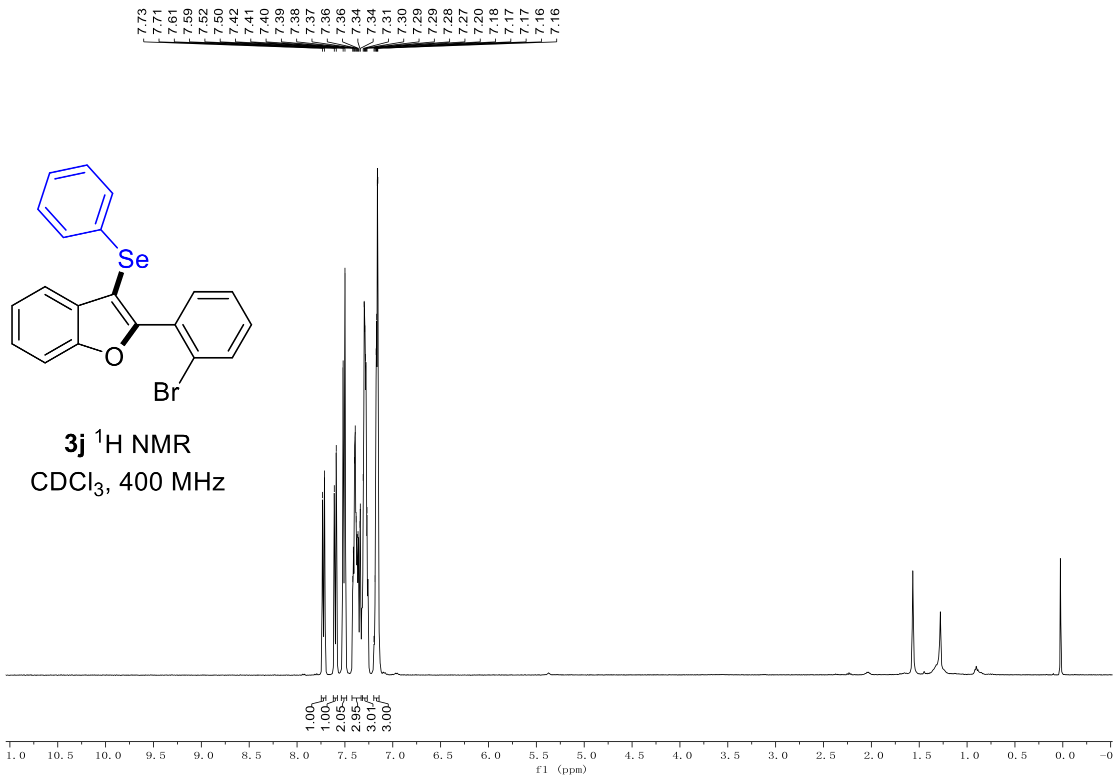
7.73 7.71 7.61 7.59 7.52 7.50 7.42 7.41 7.40 7.39 7.38 7.37 7.36 7.36 7.34 7.34 7.31 7.30 7.29 7.29 7.28 7.27 7.20 7.18 7.17 7.17 7.16 7.16
Se
O
Br
3j ^{1}H NMR
$CDCl_3$, 400 MHz
1.00 1.00 2.05 2.95 3.01 3.00
f1 (ppm)

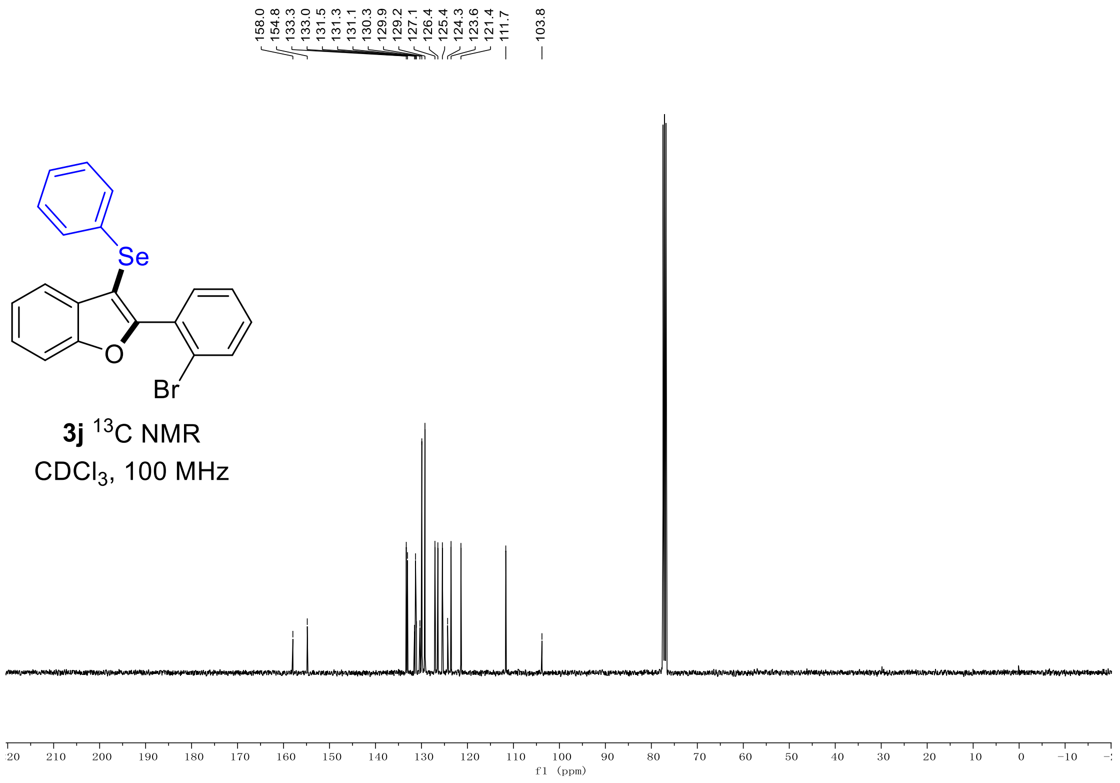
158.0 154.8 133.3 133.0 131.5 131.3 131.1 130.3 129.9 129.2 127.1 126.4 125.4 124.3 123.6 121.4 111.7 103.8
Se
O
Br
3j ^{13}C NMR
$CDCl_3$, 100 MHz
f1 (ppm)

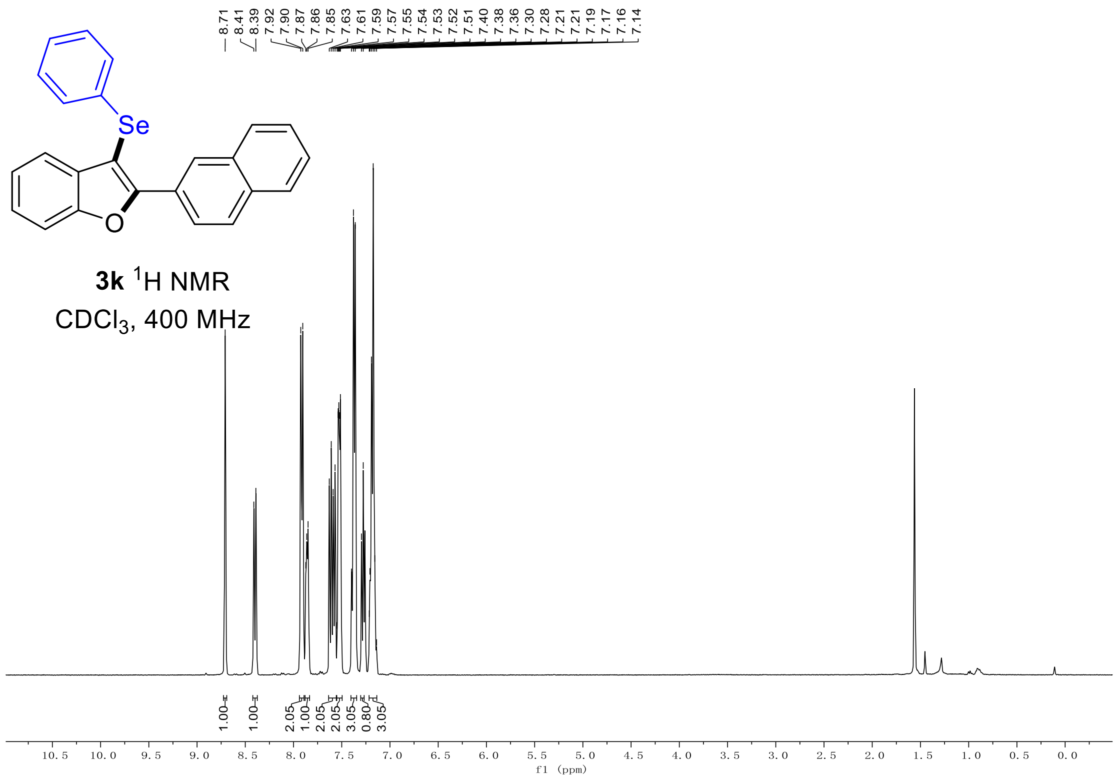
8.71
8.41
8.39
7.92
7.90
7.87
7.86
7.85
7.63
7.61
7.59
7.57
7.55
7.54
7.53
7.52
7.51
7.40
7.38
7.36
7.30
7.28
7.21
7.21
7.19
7.17
7.16
7.14
Se
O
3k ^{1}H NMR
$CDCl_3$, 400 MHz
1.00
1.00
2.05
1.00
2.05
2.05
3.05
0.80
3.05
10.5 10.0 9.5 9.0 8.5 8.0 7.5 7.0 6.5 6.0 5.5 5.0 4.5 4.0 3.5 3.0 2.5 2.0 1.5 1.0 0.5 0.0
f1 (ppm)

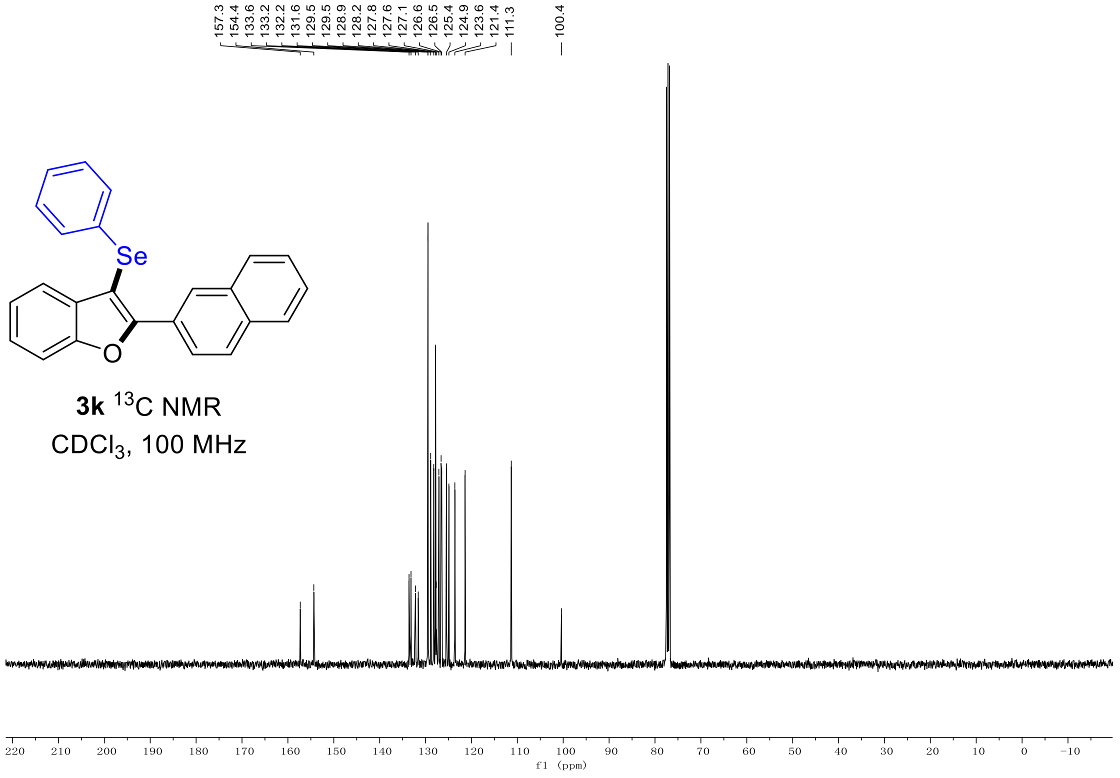
157.3
154.4
133.6
133.2
132.2
131.6
129.5
129.5
128.9
128.2
127.8
127.6
127.1
126.6
126.5
125.4
124.9
123.6
121.4
111.3
100.4
Se
O
3k ^{13}C NMR
$CDCl_3$, 100 MHz
220 210 200 190 180 170 160 150 140 130 120 110 100 90 80 70 60 50 40 30 20 10 0 -10
f1 (ppm)

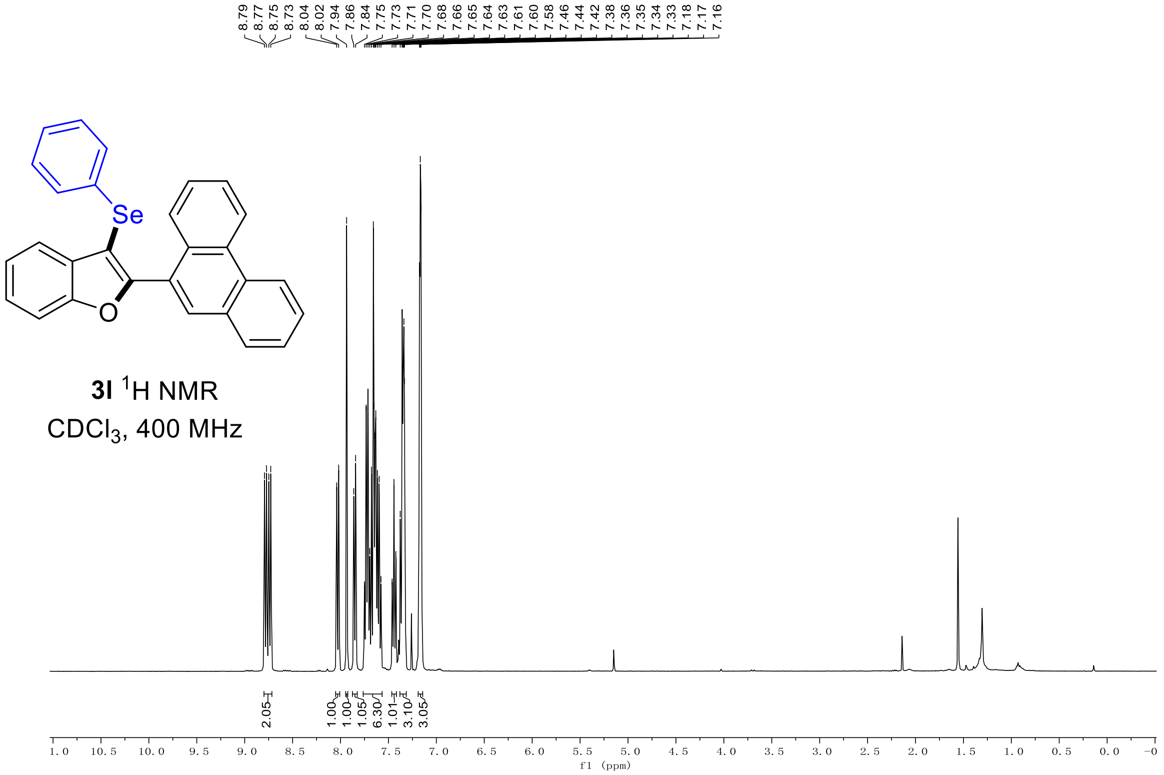
8.79 8.77 8.75 8.73 8.04 8.02 7.94 7.86 7.84 7.75 7.73 7.71 7.70 7.68 7.66 7.65 7.64 7.63 7.61 7.60 7.58 7.46 7.44 7.42 7.38 7.36 7.35 7.34 7.33 7.18 7.17 7.16
Se
O
3l 1H NMR
CDCl3, 400 MHz
2.05 1.00 1.00 1.05 6.30 1.01 3.10 3.05
f1 (ppm)

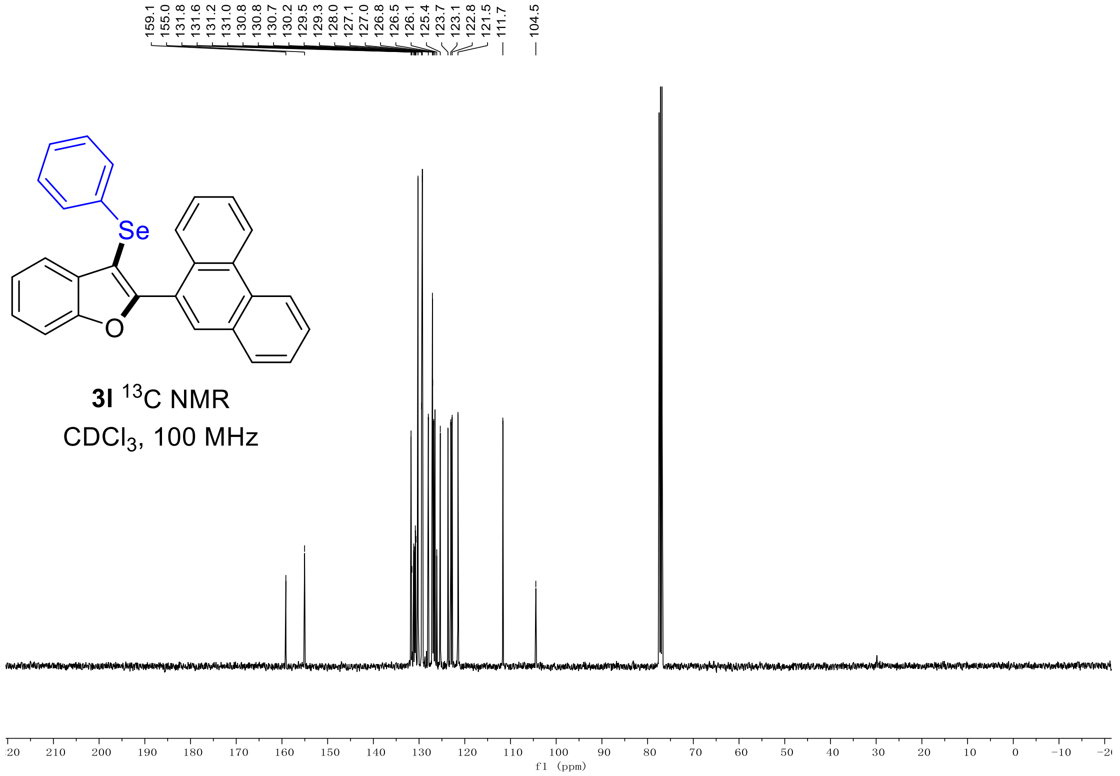
159.1 155.0 131.8 131.6 131.2 131.0 130.8 130.8 130.7 130.2 129.5 129.3 128.0 127.1 127.0 126.8 126.5 126.1 125.4 123.7 123.1 122.8 121.5 111.7 104.5
Se
O
3l 13C NMR
CDCl3, 100 MHz
f1 (ppm)

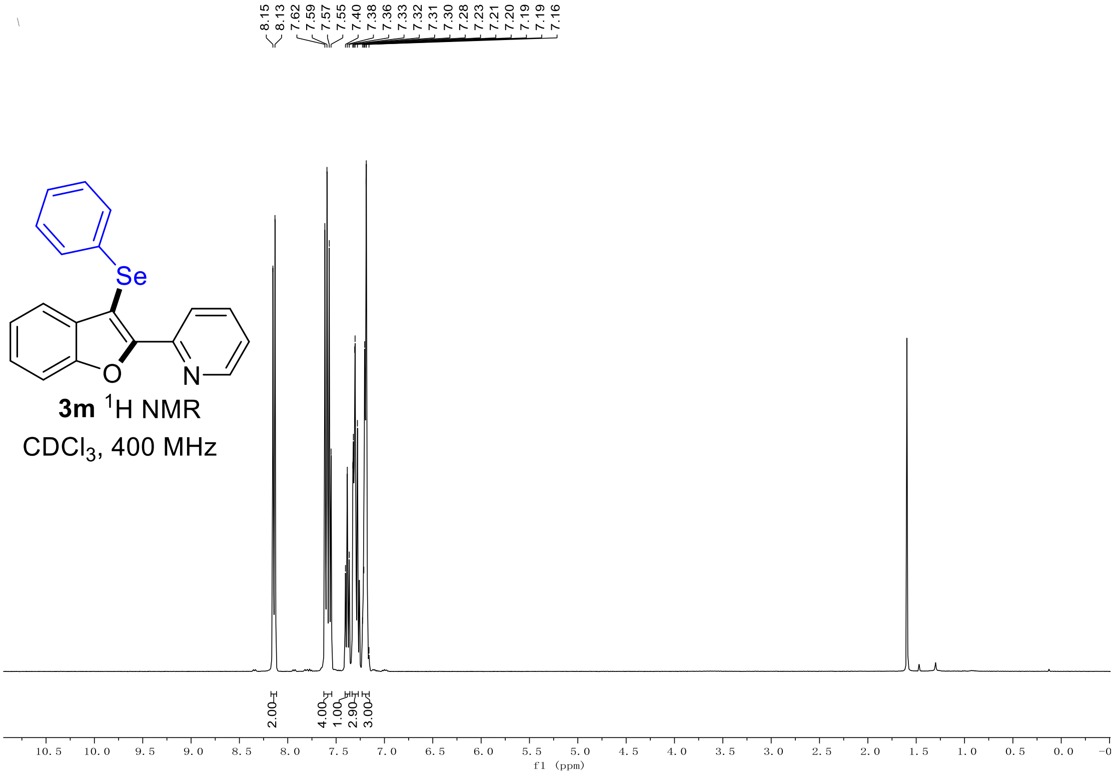
8.15
8.13
7.62
7.59
7.57
7.55
7.40
7.38
7.36
7.33
7.32
7.31
7.30
7.28
7.23
7.21
7.20
7.19
7.19
7.16
Se
O
N
3m 1H NMR
CDCl3, 400 MHz
2.00
4.00
1.00
2.90
3.00
f1 (ppm)

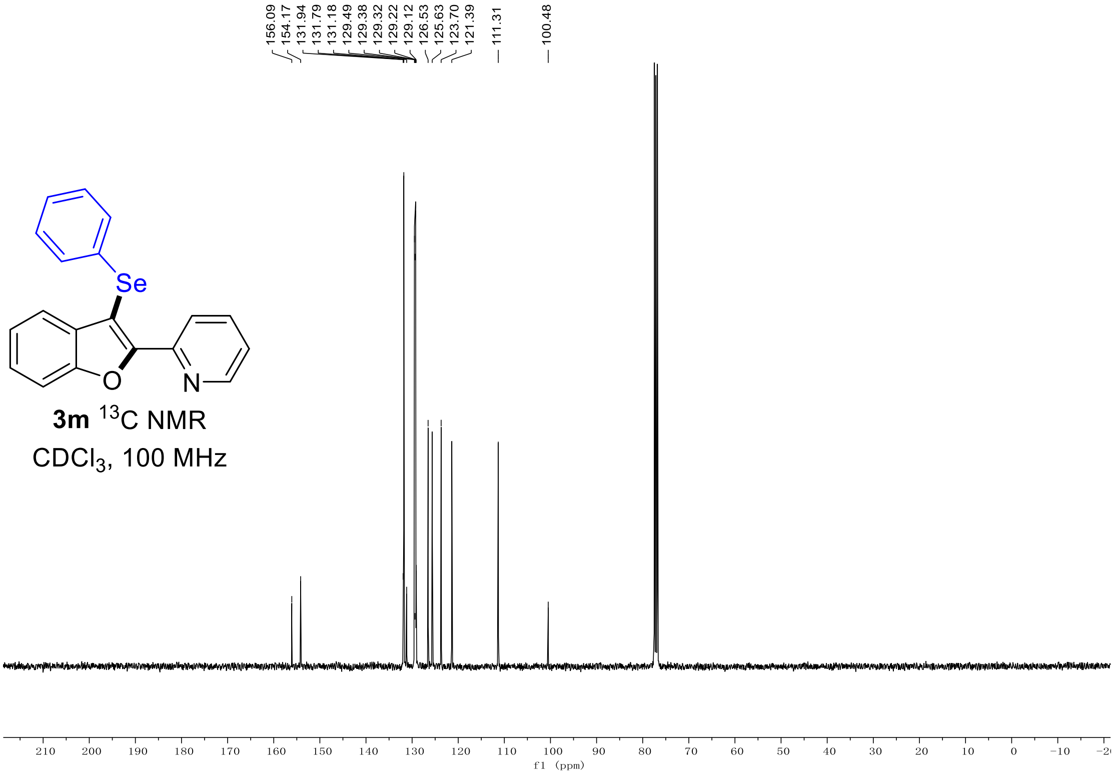
156.09
154.17
131.94
131.79
131.18
129.49
129.38
129.32
129.22
129.12
126.53
125.63
123.70
121.39
111.31
100.48
Se
O
N
3m 13C NMR
CDCl3, 100 MHz
f1 (ppm)

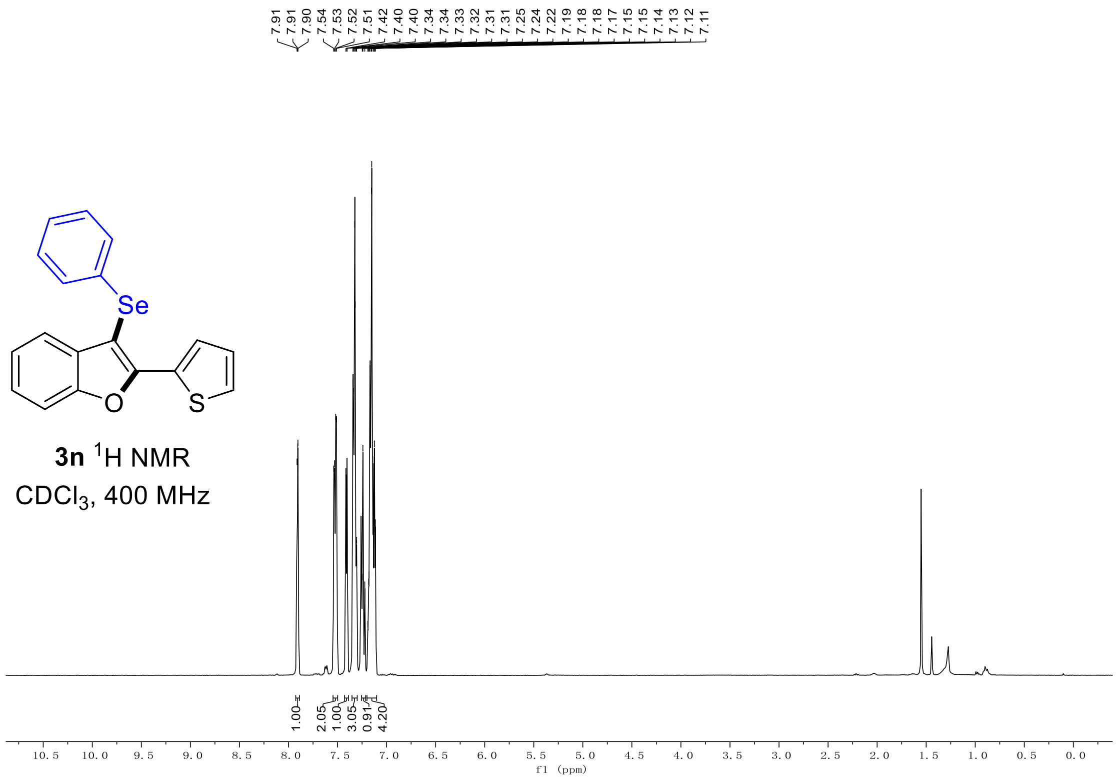
7.91 7.91 7.90 7.54 7.53 7.52 7.51 7.42 7.40 7.40 7.34 7.34 7.33 7.32 7.31 7.31 7.25 7.24 7.22 7.19 7.18 7.18 7.17 7.15 7.15 7.14 7.13 7.12 7.11
Se
O
S
3n 1H NMR
CDCl3, 400 MHz
1.00 2.05 1.00 3.05 0.91 4.20
f1 (ppm)

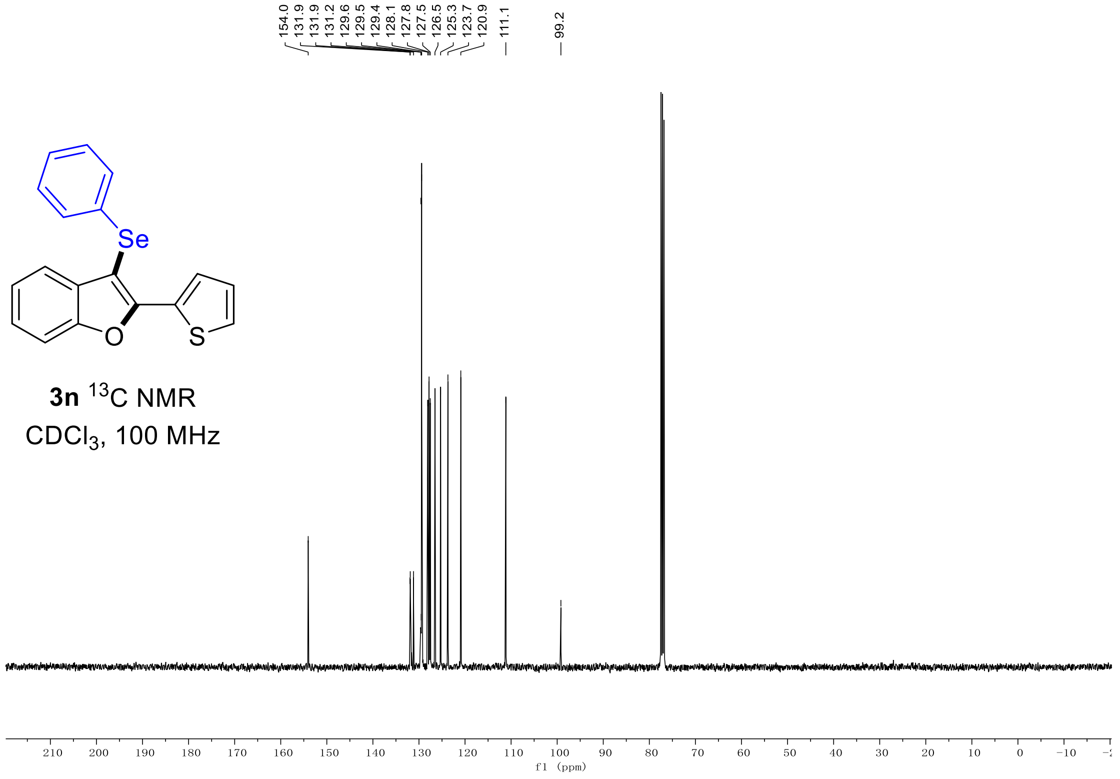
154.0 131.9 131.9 131.2 129.6 129.5 129.4 128.1 127.8 127.5 126.5 125.3 123.7 120.9 111.1 99.2
Se
O
S
3n 13C NMR
CDCl3, 100 MHz
f1 (ppm)

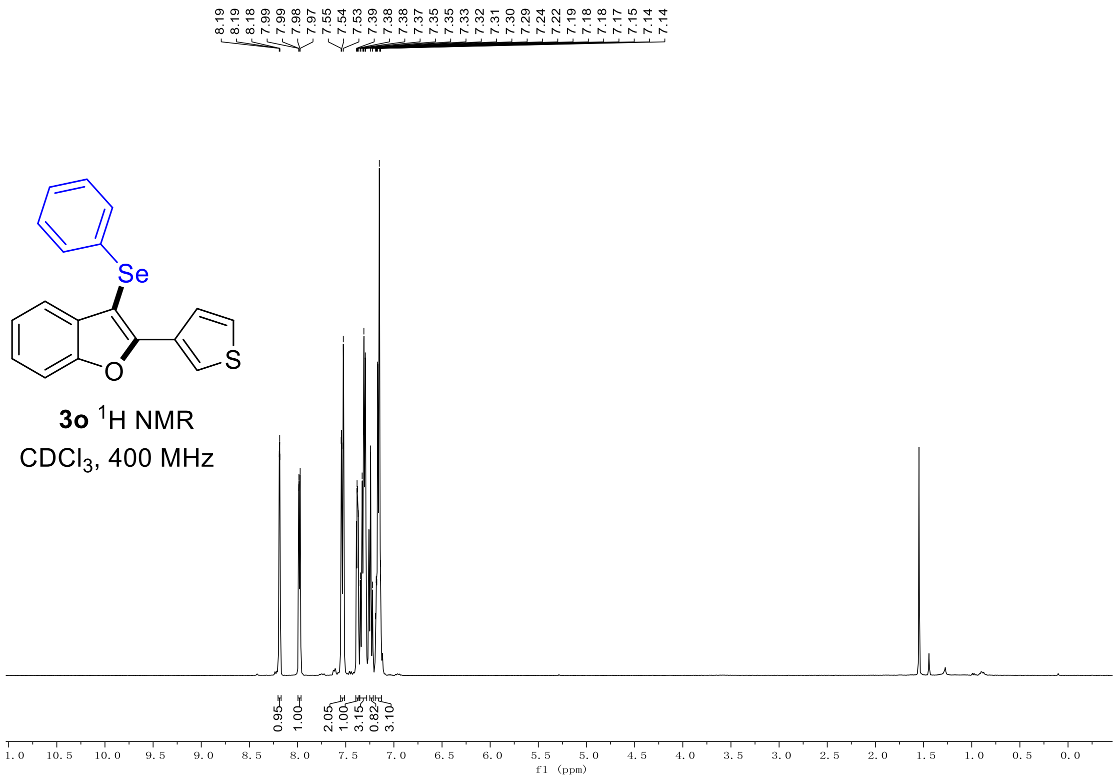
8.19
8.19
8.18
7.99
7.99
7.98
7.97
7.55
7.54
7.53
7.39
7.38
7.38
7.37
7.35
7.35
7.33
7.32
7.31
7.30
7.29
7.24
7.22
7.19
7.18
7.18
7.17
7.15
7.14
7.14
Se
O
S
3o ^{1}H NMR
$CDCl_3$, 400 MHz
0.95
1.00
2.05
1.00
3.15
0.82
3.10
f1 (ppm)

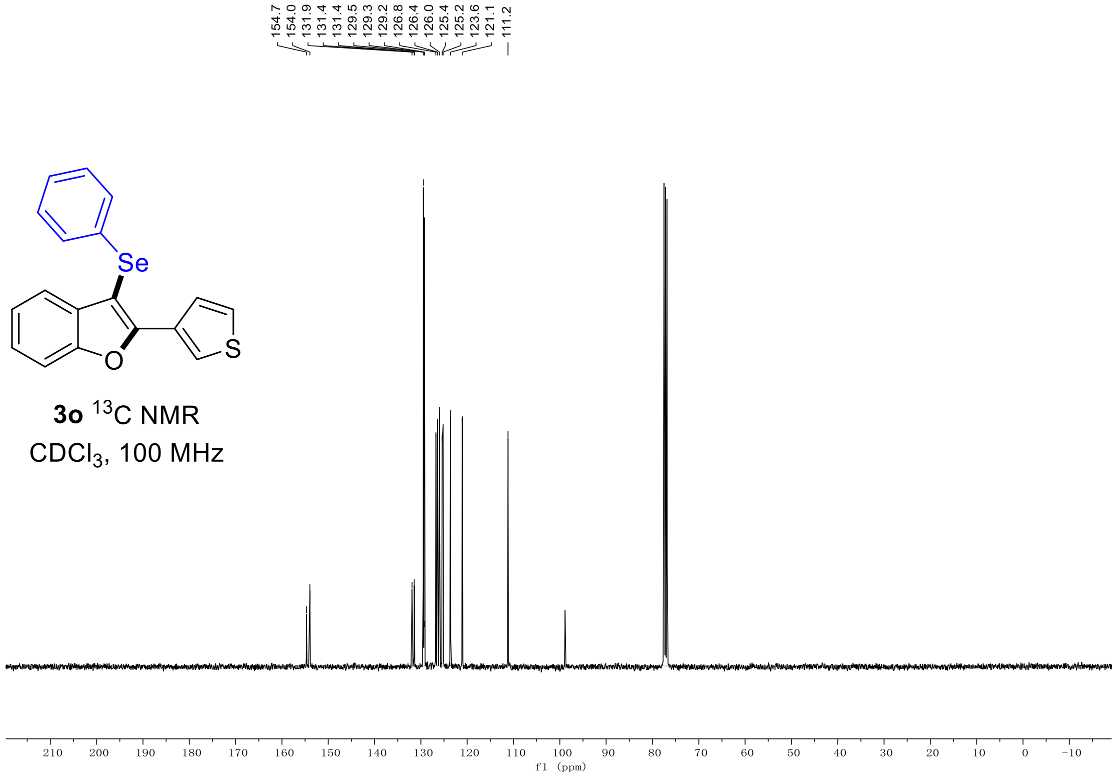
154.7
154.0
131.9
131.4
131.4
129.5
129.3
129.2
126.8
126.4
126.0
125.4
125.2
123.6
121.1
111.2
Se
O
S
3o ^{13}C NMR
$CDCl_3$, 100 MHz
f1 (ppm)

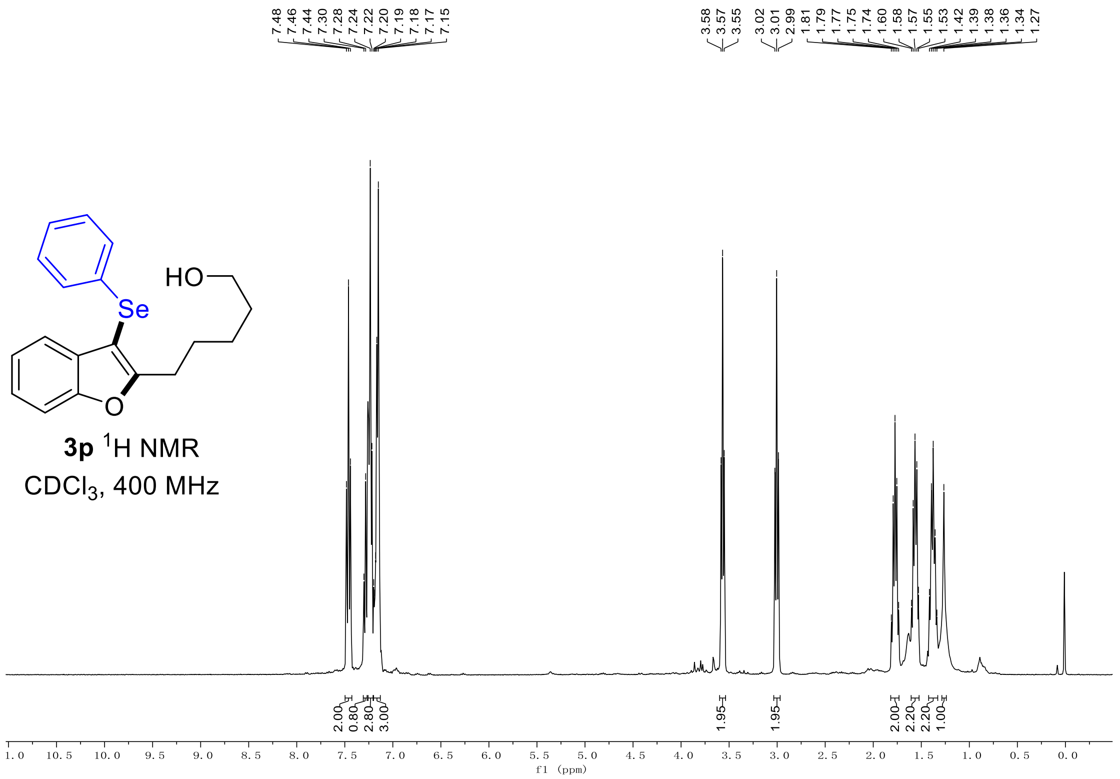
7.48 7.46 7.44 7.30 7.28 7.24 7.22 7.20 7.19 7.18 7.17 7.15
3.58 3.57 3.55 3.02 3.01 2.99 1.81 1.79 1.77 1.75 1.74 1.60 1.58 1.57 1.55 1.53 1.42 1.39 1.38 1.36 1.34 1.27
HO
Se
O
3p 1H NMR
CDCl3, 400 MHz
2.00 0.80 2.80 3.00 1.95 1.95 2.00 2.20 2.20 1.00
f1 (ppm)

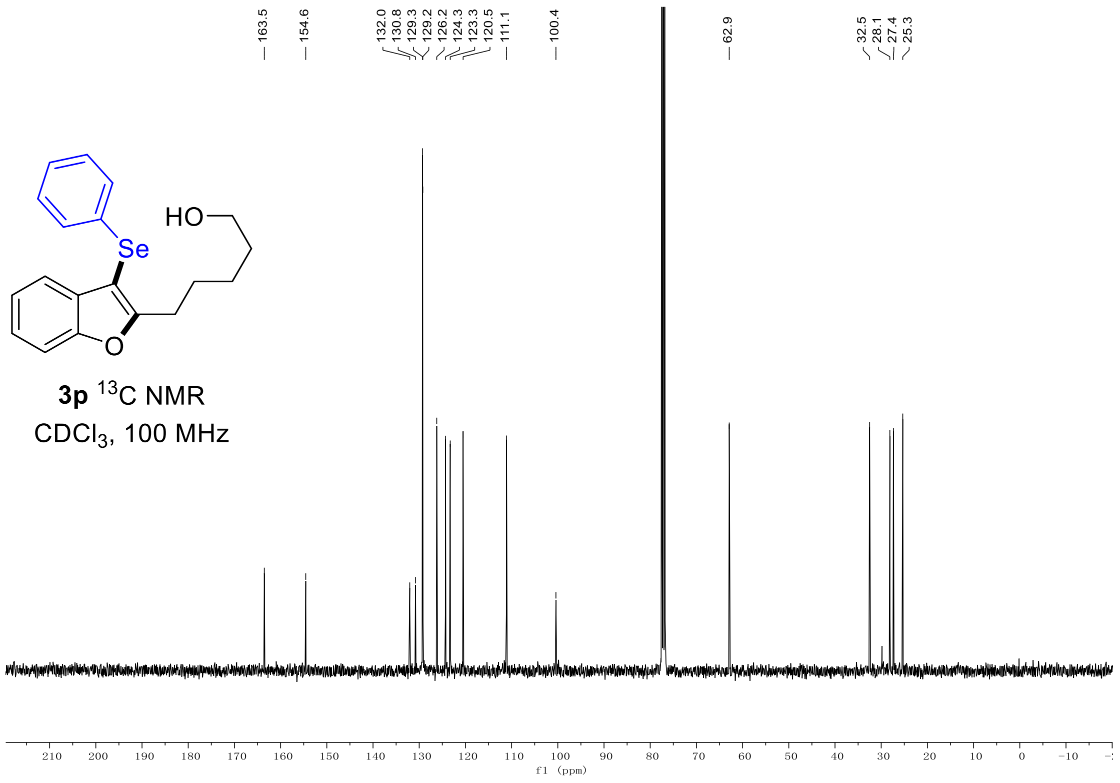
163.5 154.6 132.0 130.8 129.3 129.2 126.2 124.3 123.3 120.5 111.1 100.4 62.9 32.5 28.1 27.4 25.3
HO
Se
O
3p 13C NMR
CDCl3, 100 MHz
f1 (ppm)

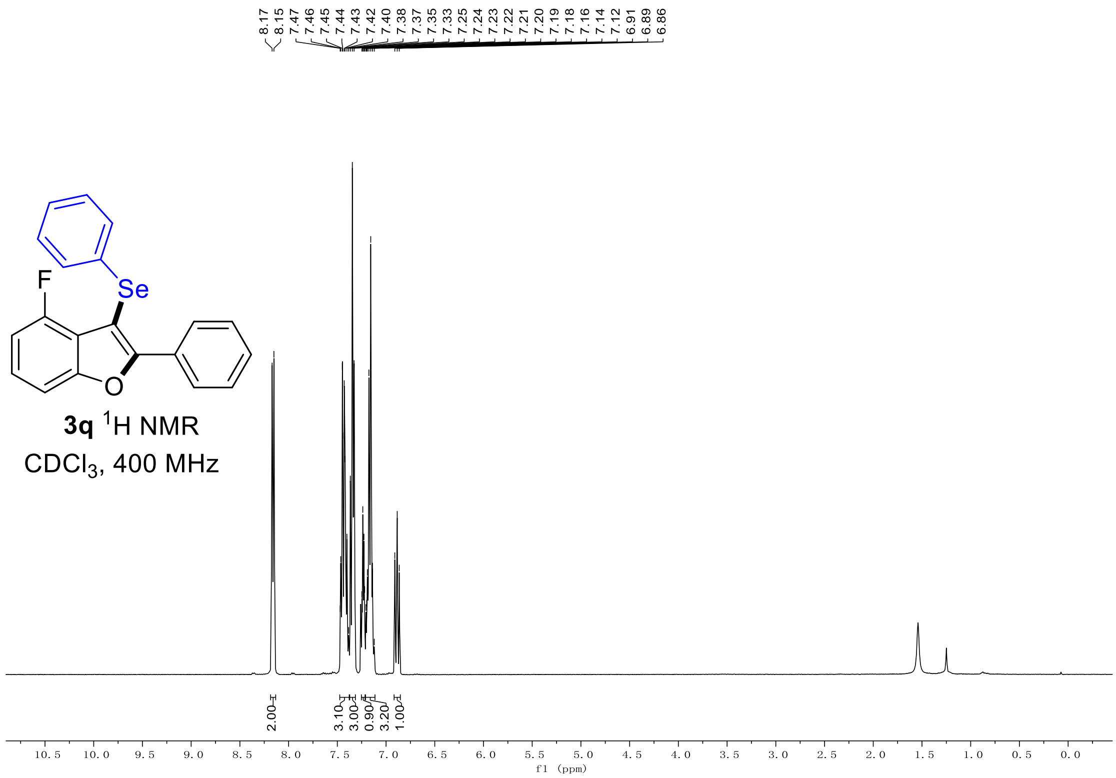

8.17
8.15
7.47
7.46
7.45
7.44
7.43
7.42
7.40
7.38
7.37
7.35
7.33
7.25
7.24
7.23
7.22
7.21
7.20
7.19
7.18
7.16
7.14
7.12
6.91
6.89
6.86
F
Se
O
3q ¹H NMR
CDCl3, 400 MHz
2.00
3.10
3.00
0.90
3.20
1.00
f1 (ppm)

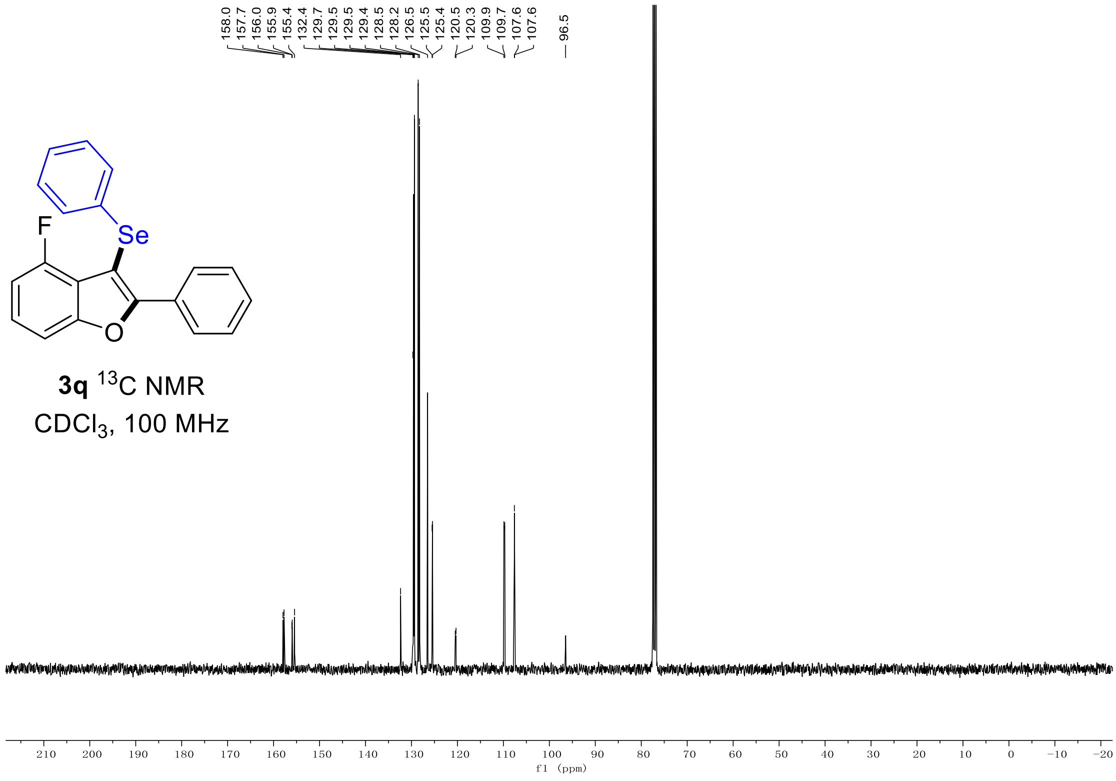

158.0
157.7
156.0
155.9
155.4
132.4
129.7
129.5
129.5
129.4
128.5
128.2
126.5
125.5
125.4
120.5
120.3
109.9
109.7
107.6
107.6
96.5
F
Se
O
3q ¹³C NMR
CDCl3, 100 MHz
f1 (ppm)

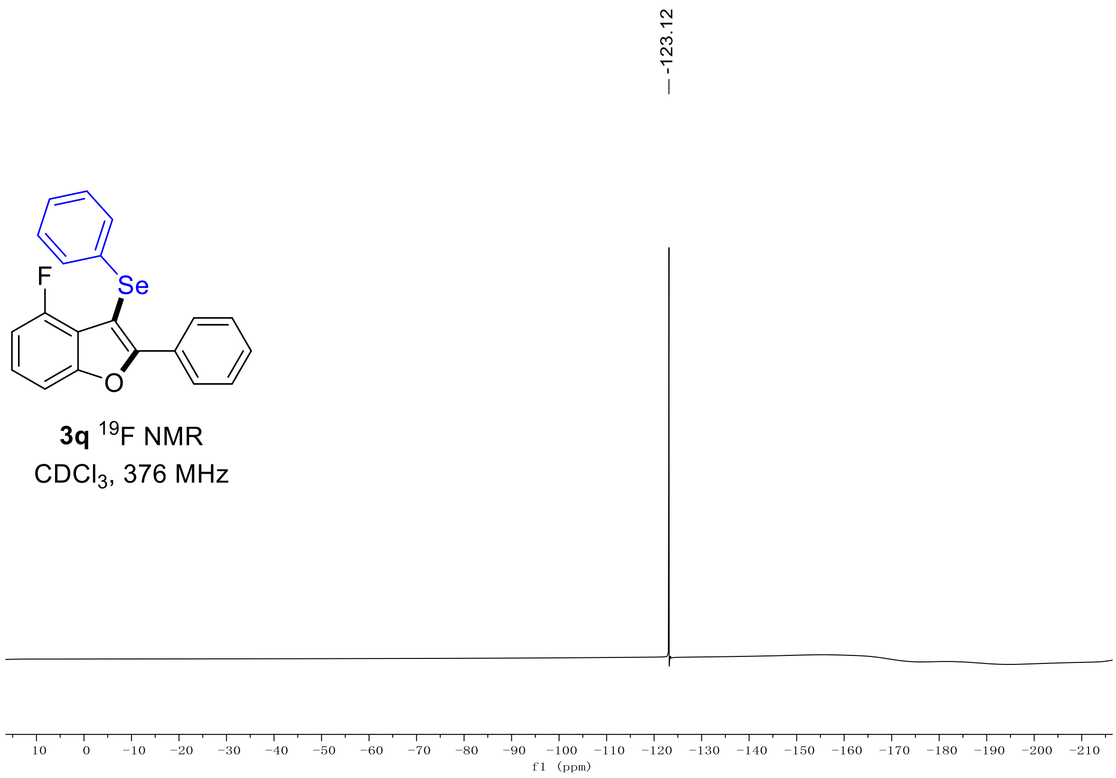
-123.12
F
Se
O
3q 19F NMR
CDCl3, 376 MHz
10 0 −10 −20 −30 −40 −50 −60 −70 −80 −90 −100 −110 −120 −130 −140 −150 −160 −170 −180 −190 −200 −210
f1 (ppm)

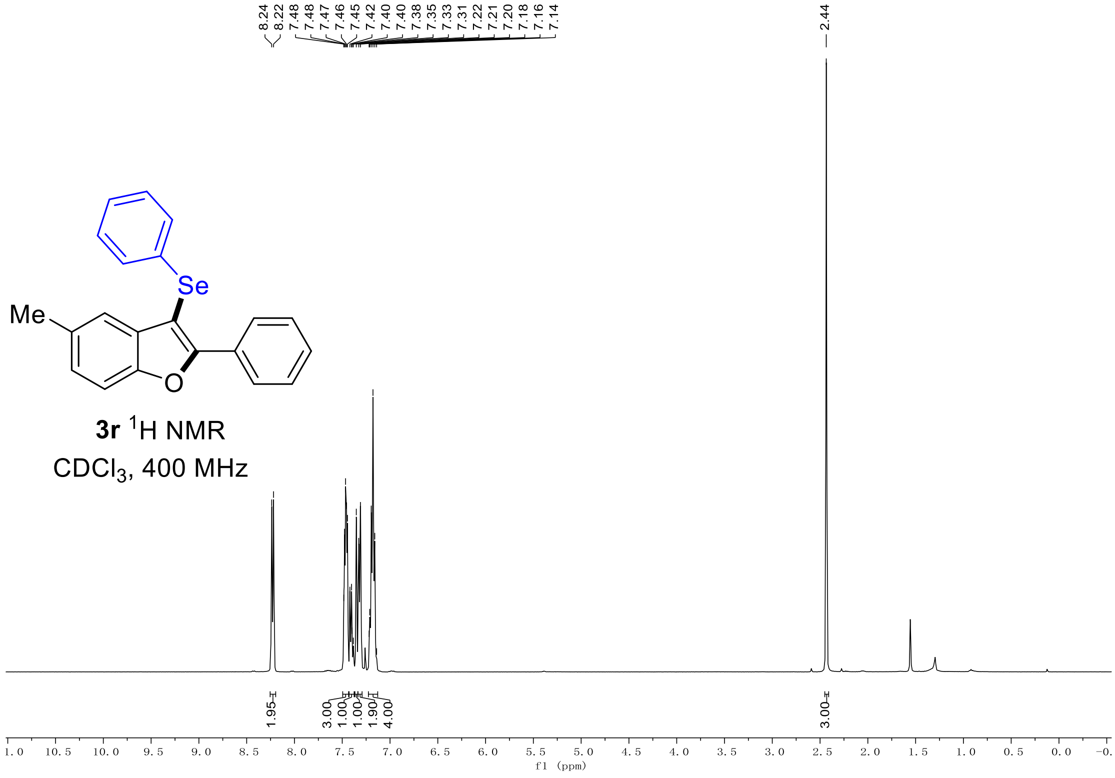
8.24
8.22
7.48
7.48
7.47
7.46
7.45
7.42
7.40
7.40
7.38
7.35
7.33
7.31
7.22
7.21
7.20
7.18
7.16
7.14
2.44
Se
Me
O
3r 1H NMR
CDCl3, 400 MHz
1.95
3.00
1.00
1.00
1.90
4.00
3.00
1.0 10.5 10.0 9.5 9.0 8.5 8.0 7.5 7.0 6.5 6.0 5.5 5.0 4.5 4.0 3.5 3.0 2.5 2.0 1.5 1.0 0.5 0.0 -0.
f1 (ppm)

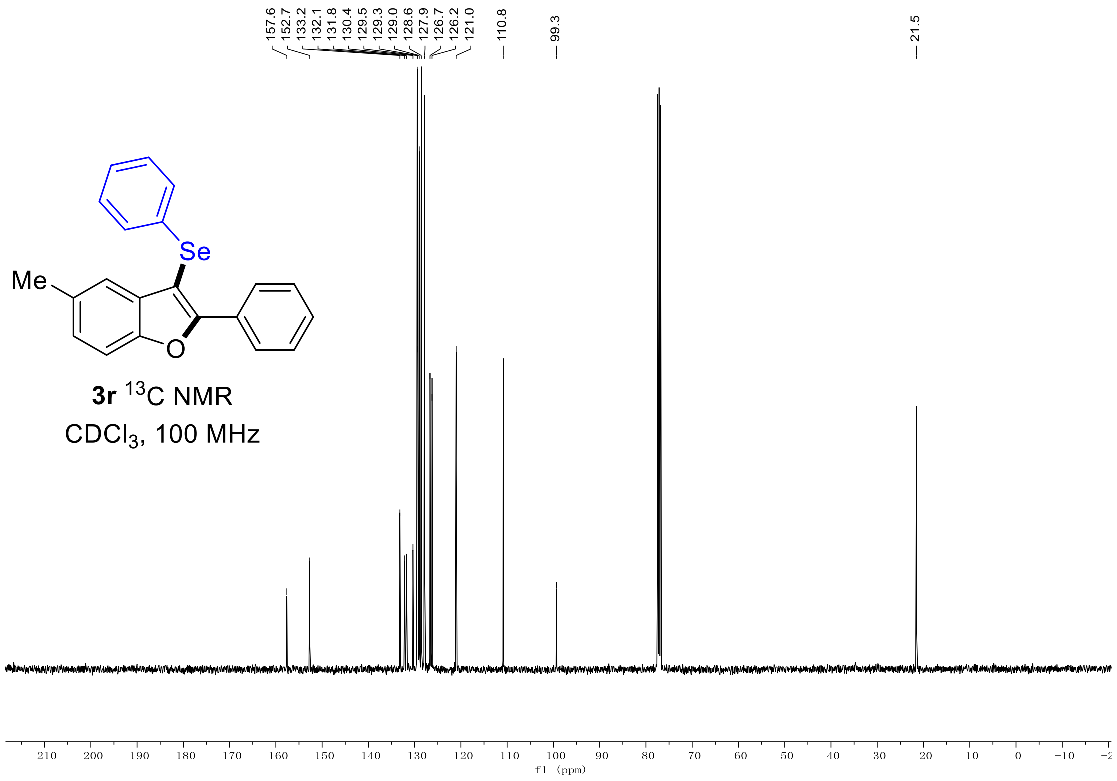
157.6
152.7
133.2
132.1
131.8
130.4
129.5
129.3
129.0
128.6
127.9
126.7
126.2
121.0
110.8
99.3
21.5
Se
Me
O
3r ^{13}C NMR
$CDCl_3$, 100 MHz
210 200 190 180 170 160 150 140 130 120 110 100 90 80 70 60 50 40 30 20 10 0 -10
f1 (ppm)

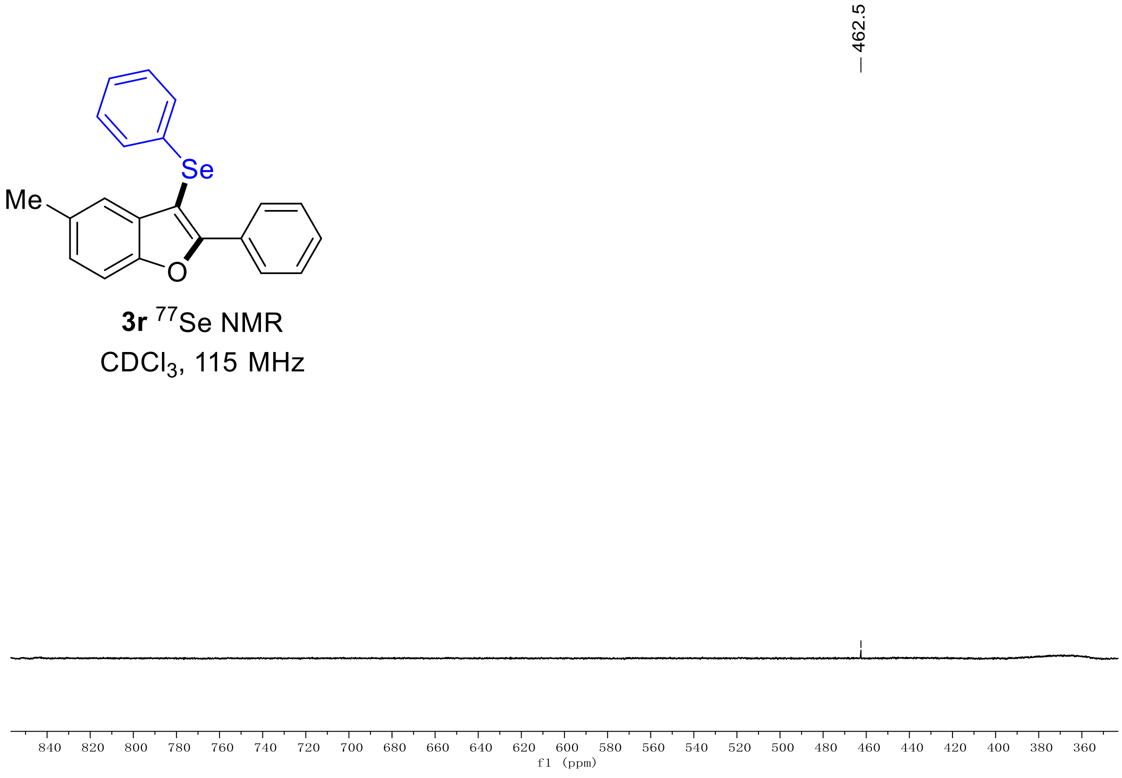
462.5
Se
Me
O
3r ^{77}Se NMR
$CDCl_3$, 115 MHz
840 820 800 780 760 740 720 700 680 660 640 620 600 580 560 540 520 500 480 460 440 420 400 380 360
f1 (ppm)

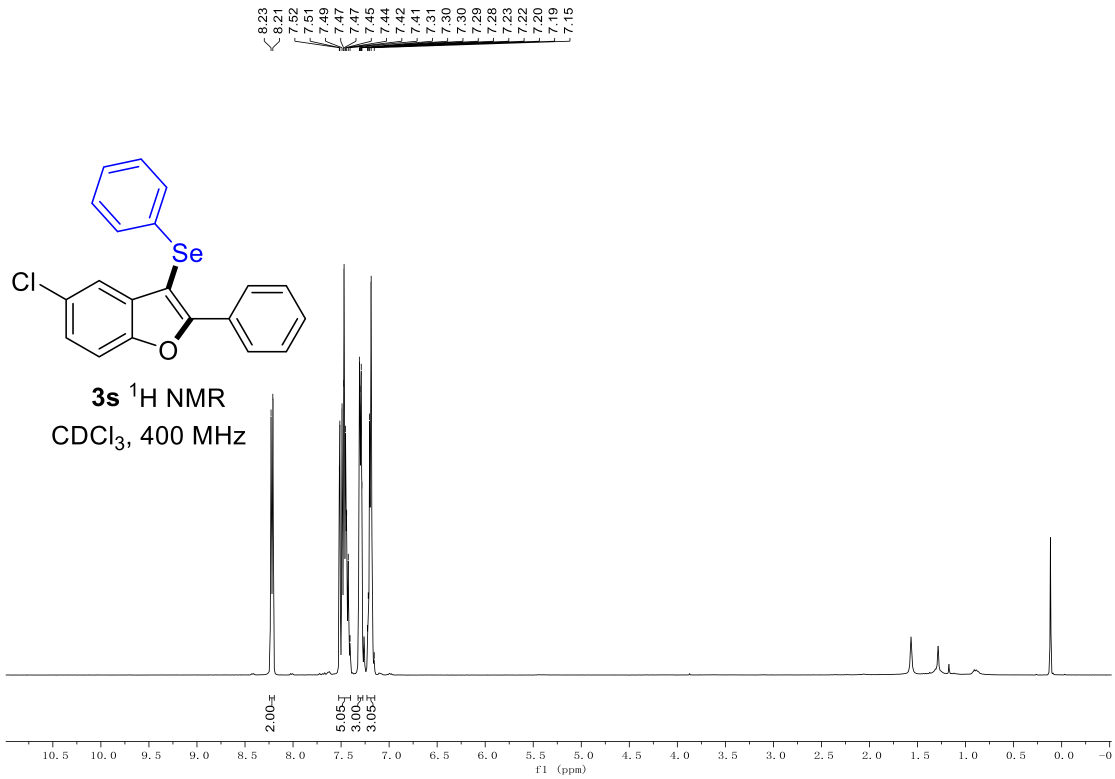
8.23
8.21
7.52
7.51
7.49
7.47
7.47
7.45
7.44
7.42
7.41
7.31
7.30
7.30
7.29
7.28
7.23
7.22
7.20
7.19
7.15
Se
Cl
O
3s 1H NMR
CDCl3, 400 MHz
2.00
5.05
3.00
3.05
f1 (ppm)

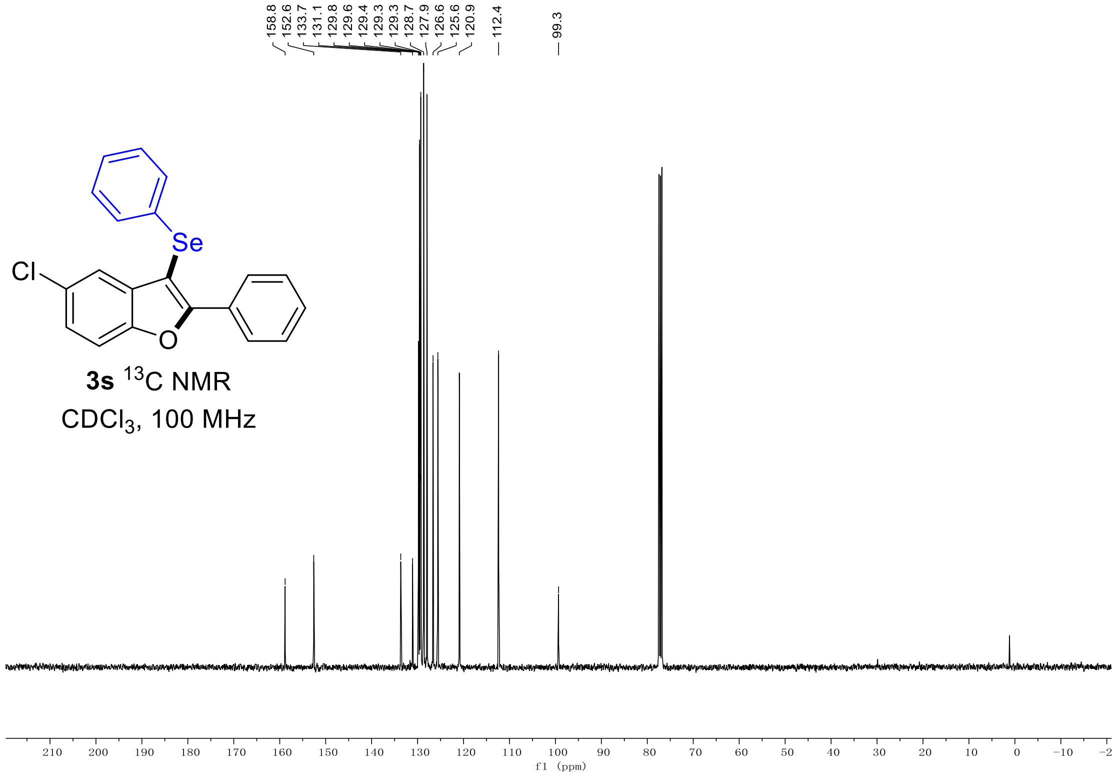
158.8
152.6
133.7
131.1
129.8
129.6
129.4
129.3
129.3
128.7
127.9
126.6
125.6
120.9
112.4
99.3
Se
Cl
O
3s 13C NMR
CDCl3, 100 MHz
f1 (ppm)

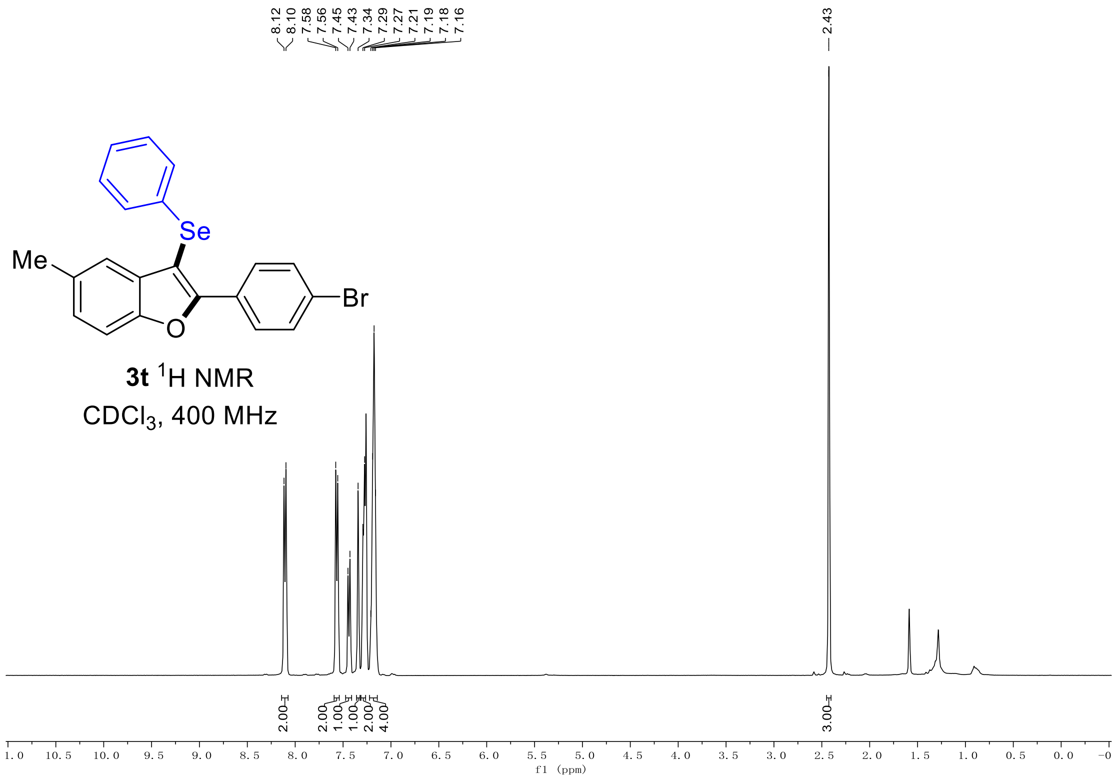
8.12
8.10
7.58
7.56
7.45
7.43
7.34
7.29
7.27
7.21
7.19
7.18
7.16
2.43
Se
Me
Br
O
3t 1H NMR
CDCl3, 400 MHz
2.00
2.00
1.00
1.00
2.00
4.00
3.00
f1 (ppm)

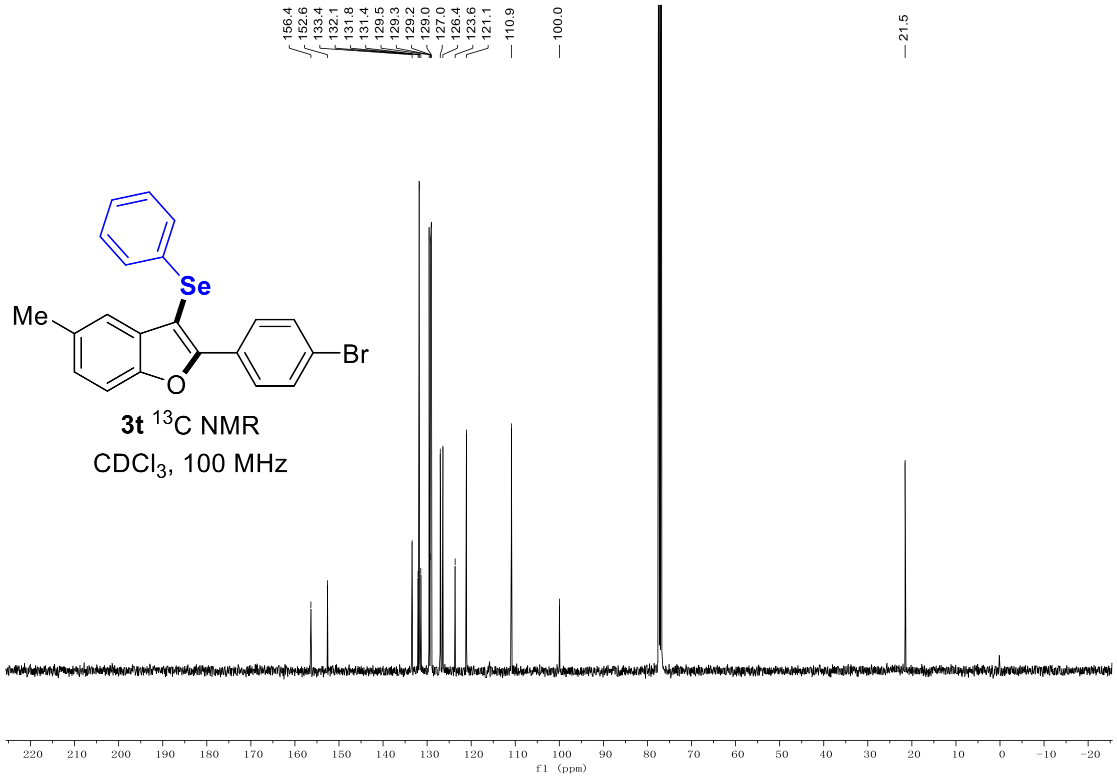
156.4
152.6
133.4
132.1
131.8
131.4
129.5
129.3
129.2
129.0
127.0
126.4
123.6
121.1
110.9
100.0
21.5
Se
Me
Br
O
3t 13C NMR
CDCl3, 100 MHz
f1 (ppm)

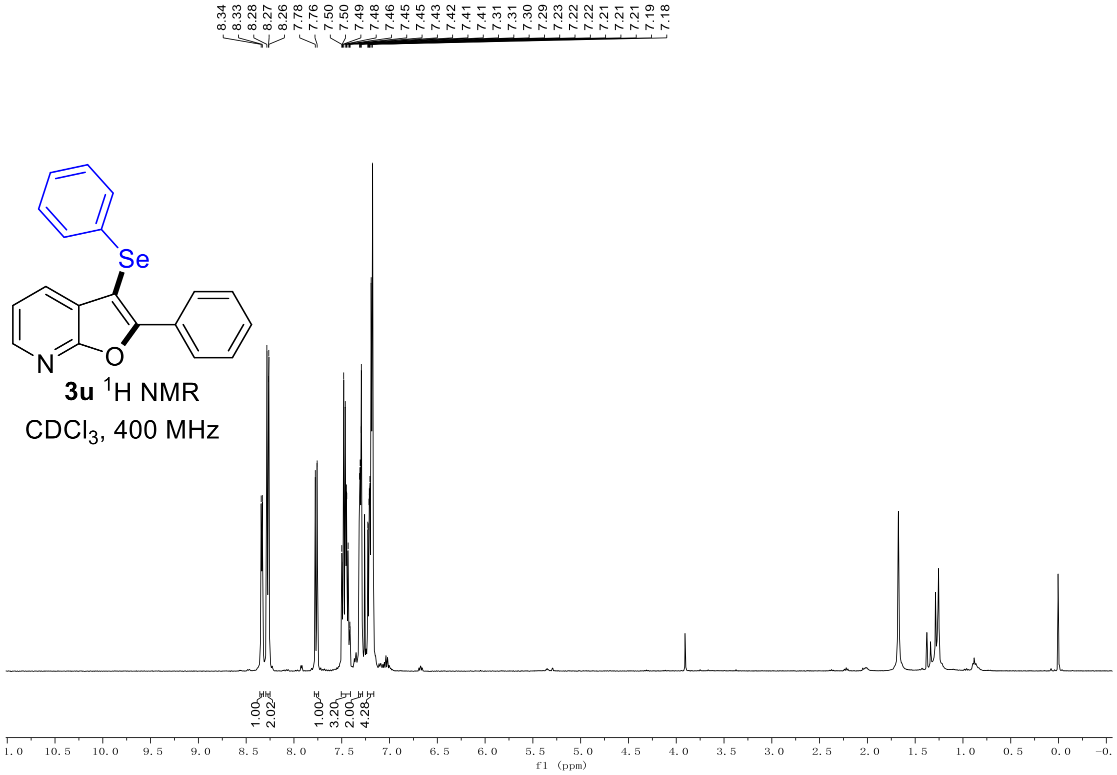
8.34
8.33
8.28
8.27
8.26
7.78
7.76
7.50
7.49
7.48
7.46
7.45
7.45
7.43
7.42
7.41
7.41
7.31
7.30
7.29
7.23
7.22
7.22
7.21
7.21
7.19
7.18
Se
N
O
3u 1H NMR
CDCl3, 400 MHz
1.00
2.02
1.00
3.20
2.00
4.28
f1 (ppm)

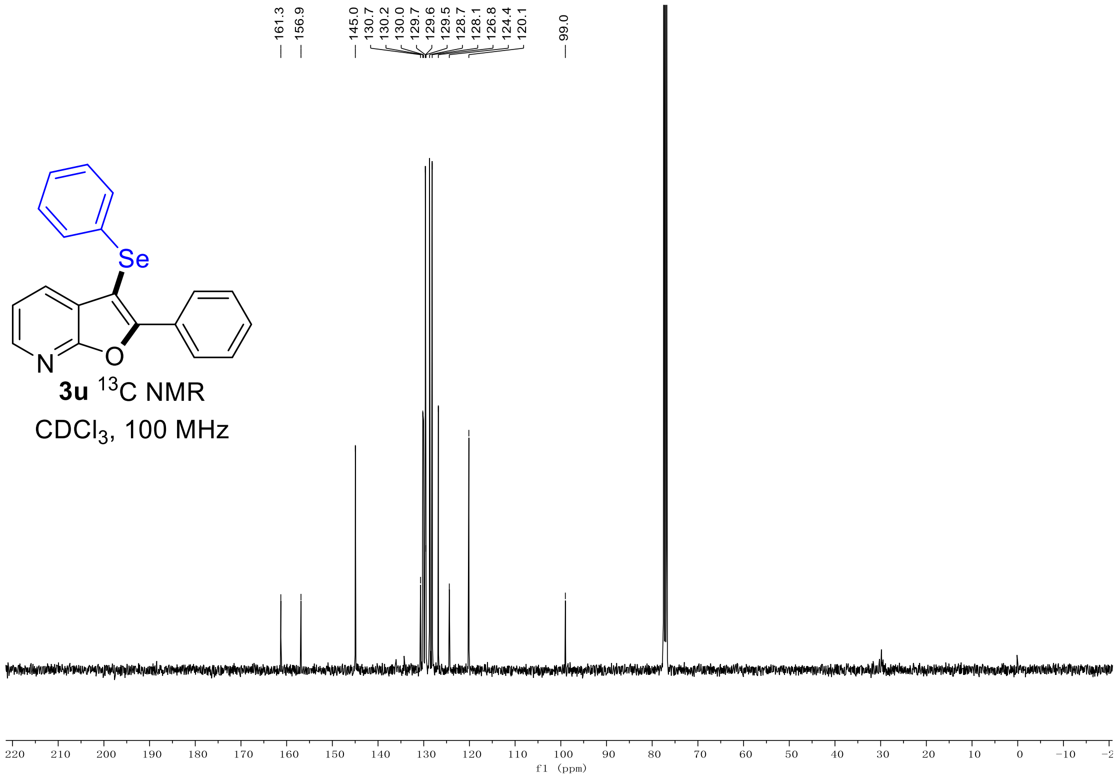
161.3
156.9
145.0
130.7
130.2
130.0
129.7
129.6
129.5
128.7
128.1
126.8
124.4
120.1
99.0
Se
N
O
3u 13C NMR
CDCl3, 100 MHz
f1 (ppm)

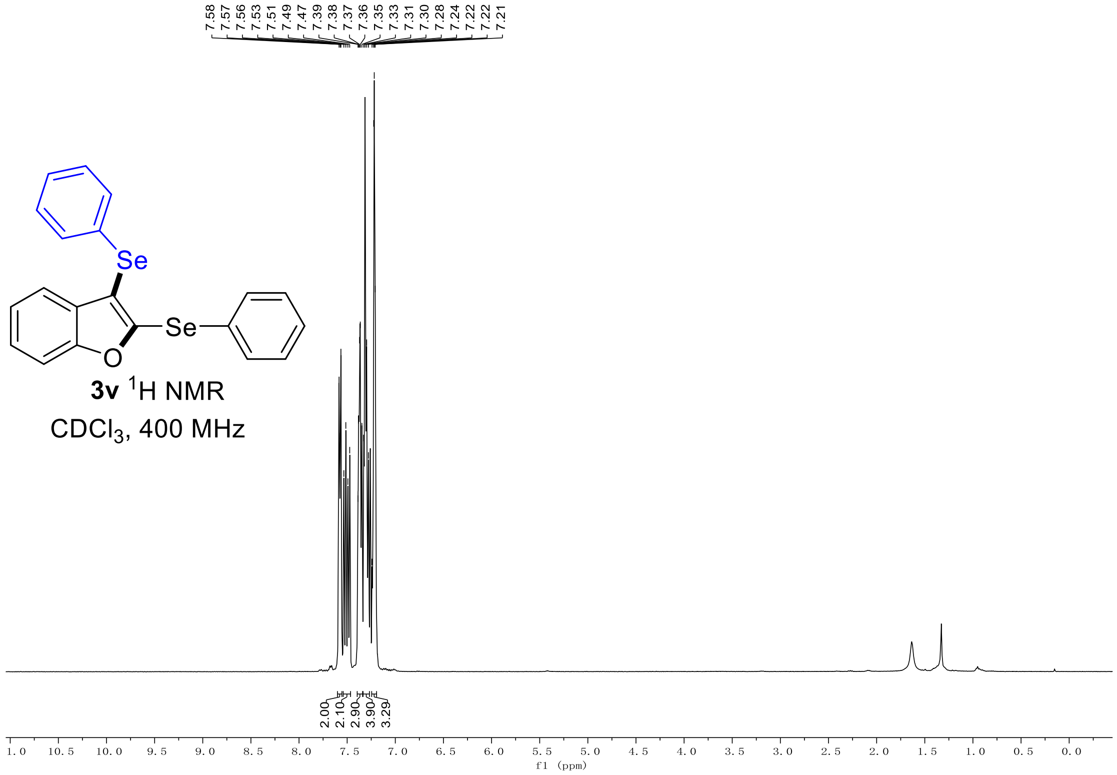
7.58
7.57
7.56
7.53
7.51
7.49
7.47
7.39
7.38
7.37
7.36
7.35
7.33
7.31
7.30
7.28
7.24
7.22
7.22
7.21
Se
Se
O
3v 1H NMR
CDCl3, 400 MHz
2.00
2.10
2.90
3.90
3.29
f1 (ppm)

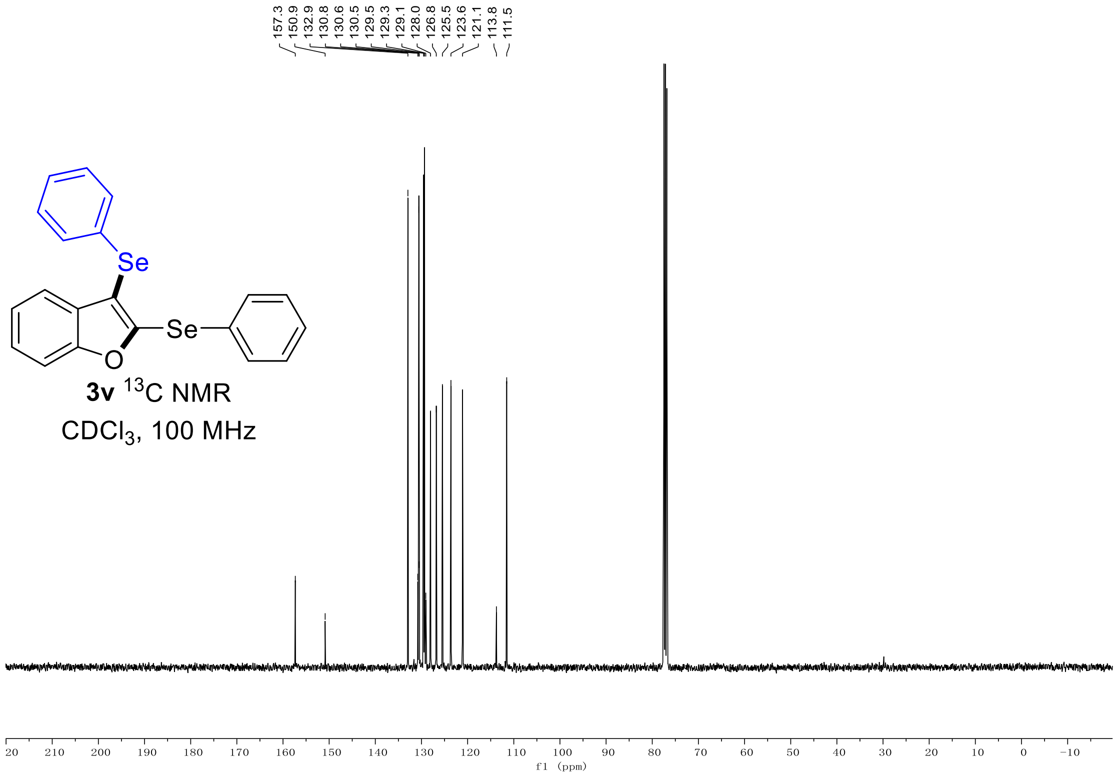
157.3
150.9
132.9
130.8
130.6
130.5
129.5
129.3
129.1
128.0
126.8
125.5
123.6
121.1
113.8
111.5
Se
Se
O
3v 13C NMR
CDCl3, 100 MHz
f1 (ppm)

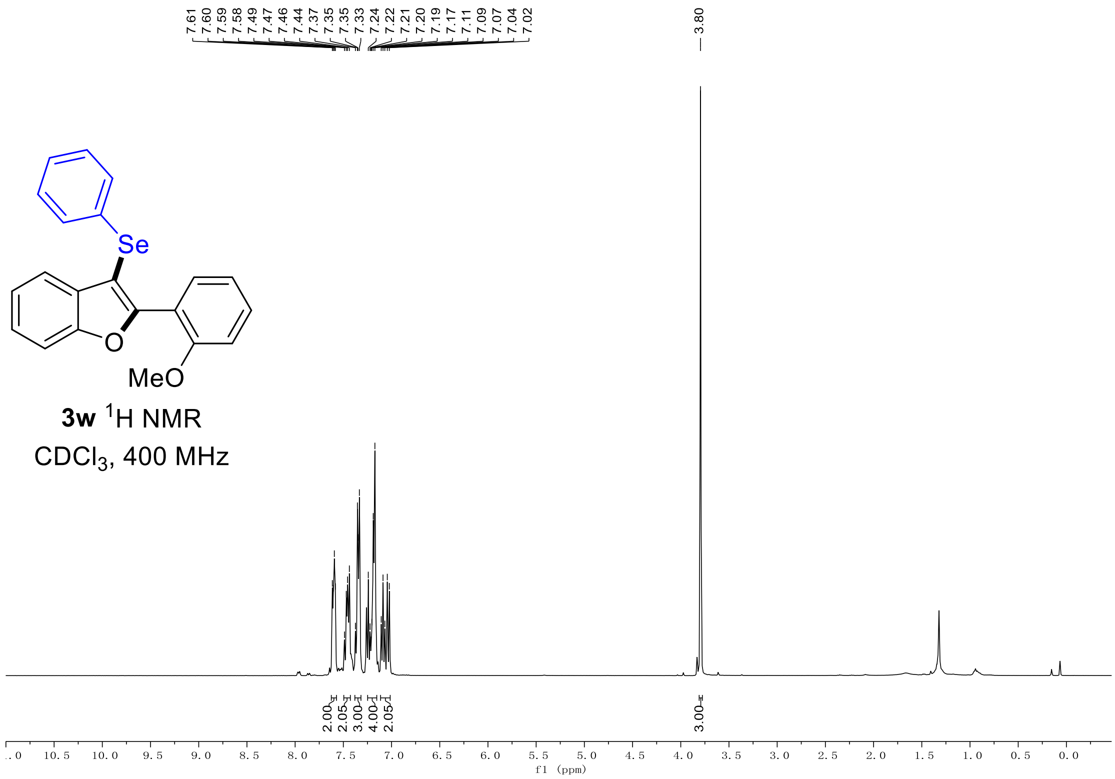
7.61 7.60 7.59 7.58 7.49 7.47 7.46 7.44 7.37 7.35 7.35 7.33 7.24 7.22 7.21 7.20 7.19 7.17 7.11 7.09 7.07 7.04 7.02
3.80
Se
O
MeO
3w ^{1}H NMR
$CDCl_3$, 400 MHz
2.00 2.05 2.05 3.00 4.00 2.05
3.00
f1 (ppm)

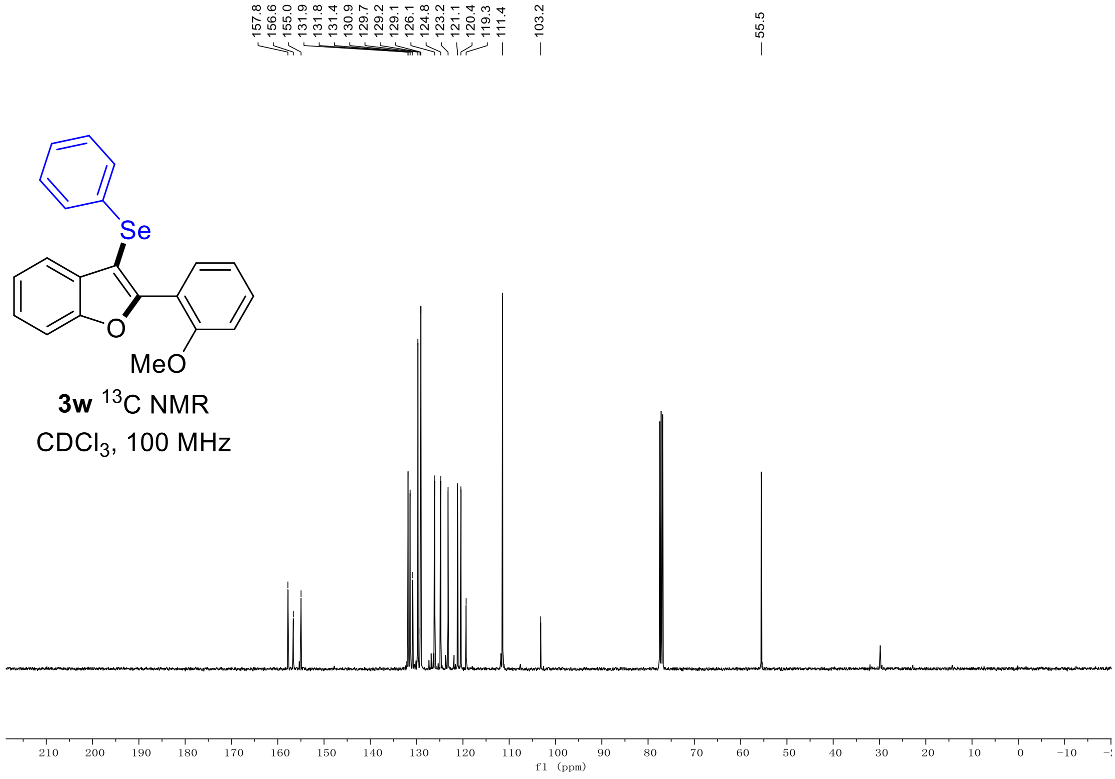
157.8 156.6 155.0 131.9 131.8 131.4 130.9 129.7 129.2 129.1 126.1 124.8 123.2 121.1 120.4 119.3 111.4
103.2
55.5
Se
O
MeO
3w ^{13}C NMR
$CDCl_3$, 100 MHz
f1 (ppm)

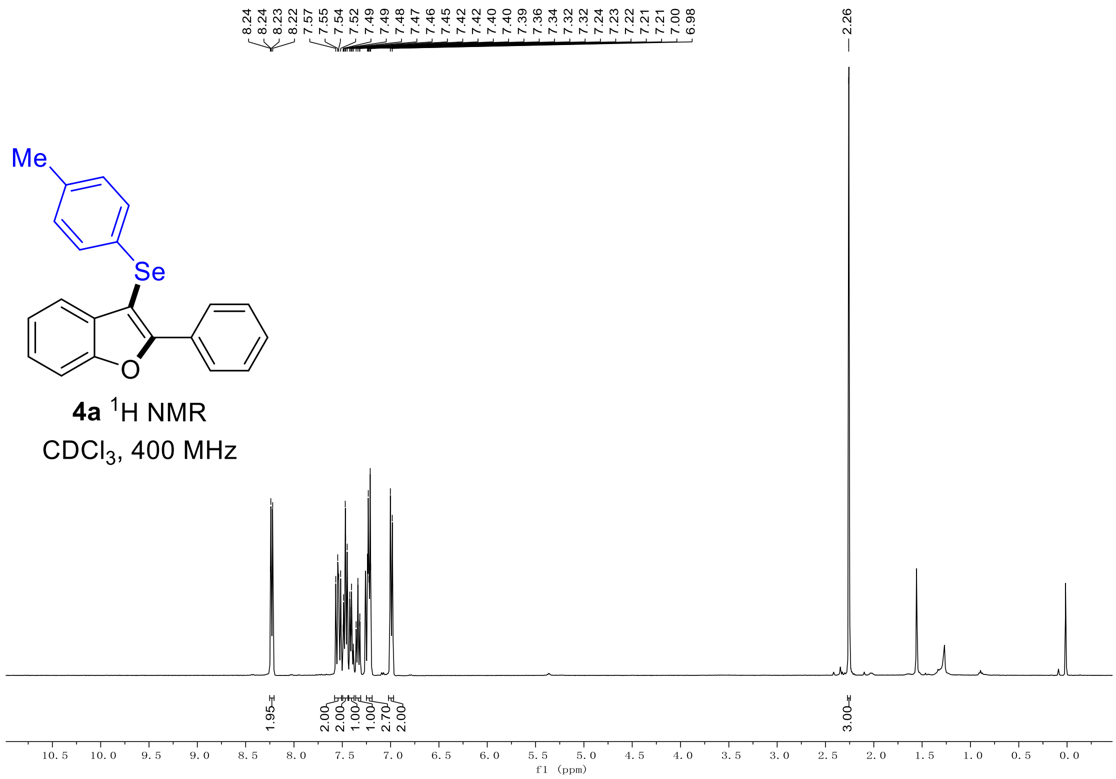
8.24
8.24
8.23
8.22
7.57
7.55
7.54
7.52
7.49
7.49
7.48
7.47
7.46
7.45
7.42
7.42
7.40
7.39
7.36
7.34
7.32
7.32
7.24
7.23
7.22
7.21
7.00
6.98
2.26
Me
Se
O
4a ¹H NMR
CDCl3, 400 MHz
1.95
2.00
2.00
1.00
1.00
2.70
2.00
3.00
f1 (ppm)

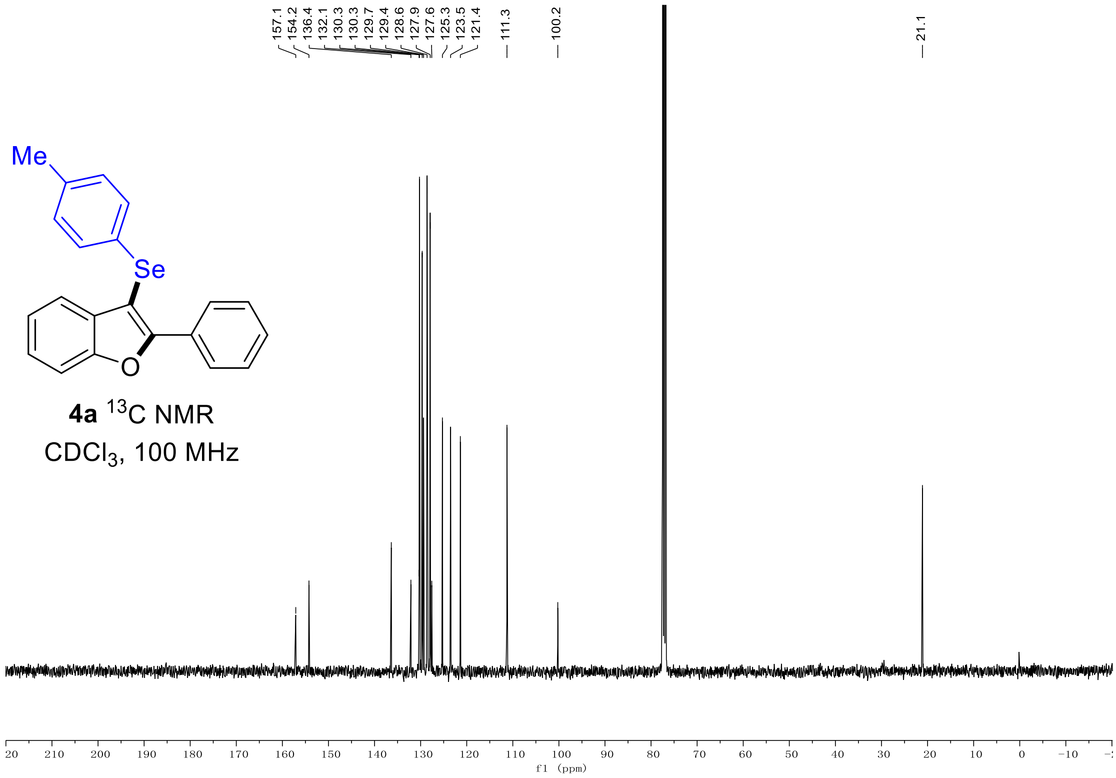
157.1
154.2
136.4
132.1
130.3
130.3
129.7
129.4
128.6
127.9
127.6
125.3
123.5
121.4
111.3
100.2
21.1
Me
Se
O
4a ¹³C NMR
CDCl3, 100 MHz
f1 (ppm)

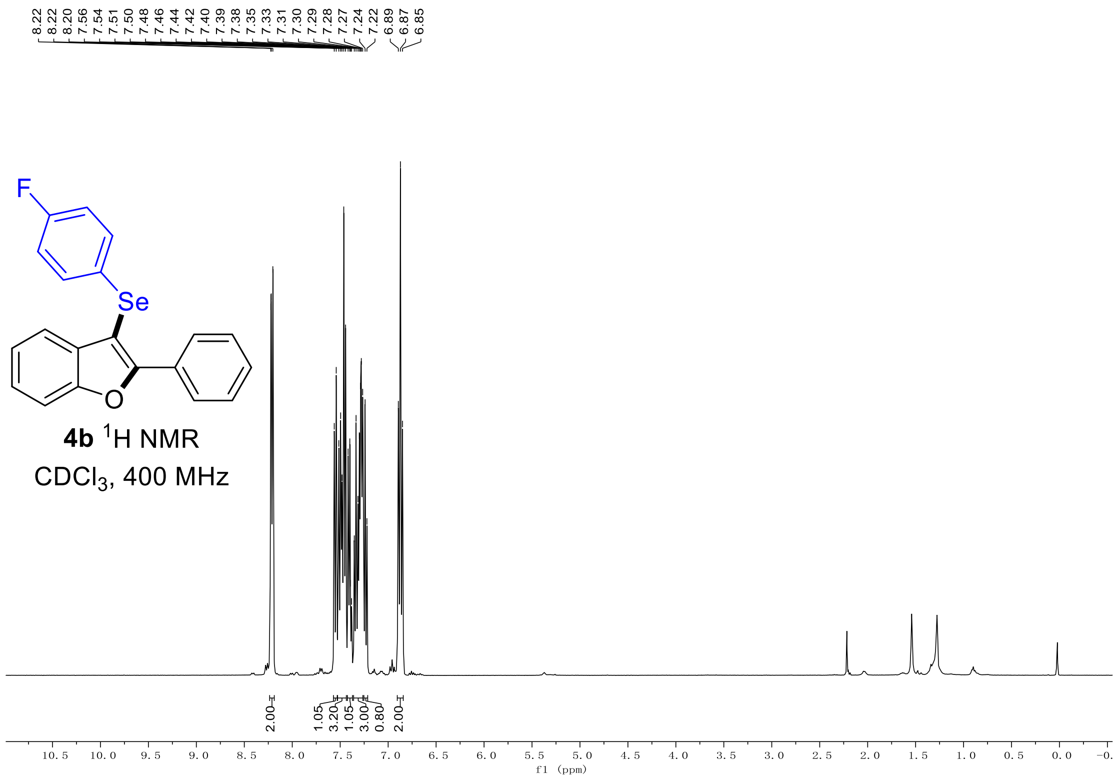
8.22 8.22 8.20 7.56 7.54 7.51 7.50 7.48 7.46 7.44 7.42 7.40 7.39 7.38 7.35 7.33 7.31 7.30 7.29 7.28 7.27 7.24 7.22 6.89 6.87 6.85
F
Se
O
4b ¹H NMR
$CDCl_3$, 400 MHz
2.00 1.05 3.20 1.05 3.00 0.80 2.00
f1 (ppm)

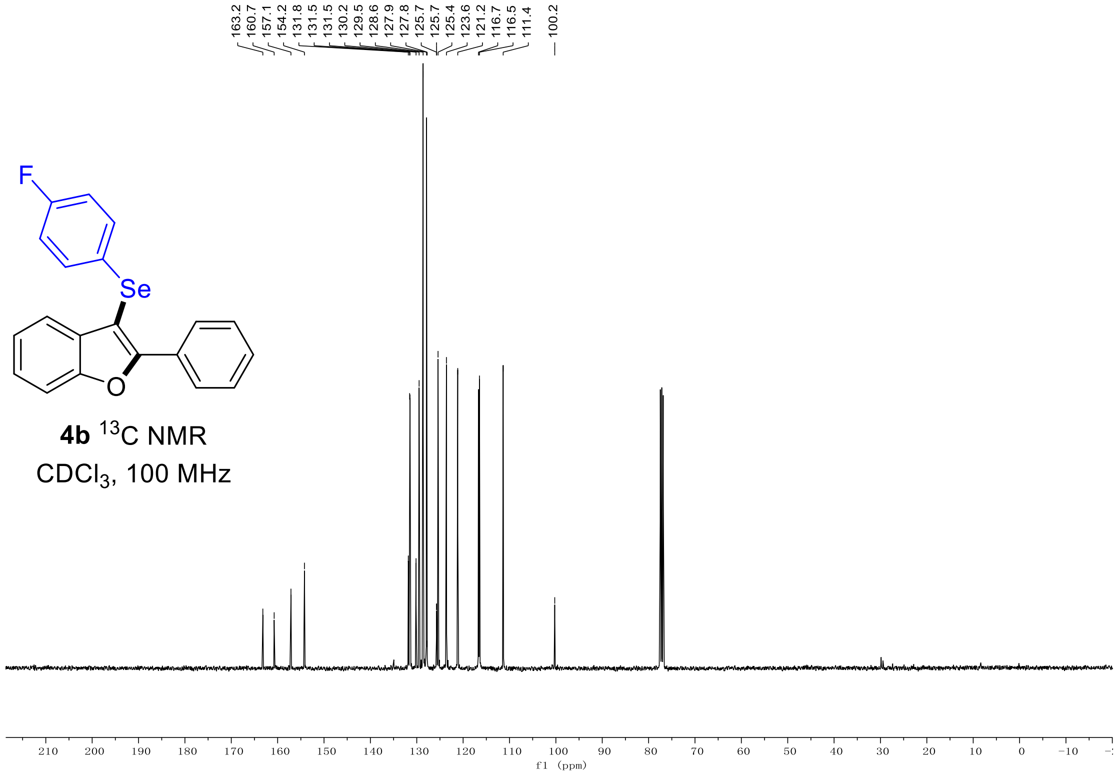
163.2 160.7 157.1 154.2 131.8 131.5 131.5 130.2 129.5 128.6 127.9 127.8 125.7 125.4 123.6 121.2 116.7 116.5 111.4 100.2
F
Se
O
4b ¹³C NMR
$CDCl_3$, 100 MHz
f1 (ppm)

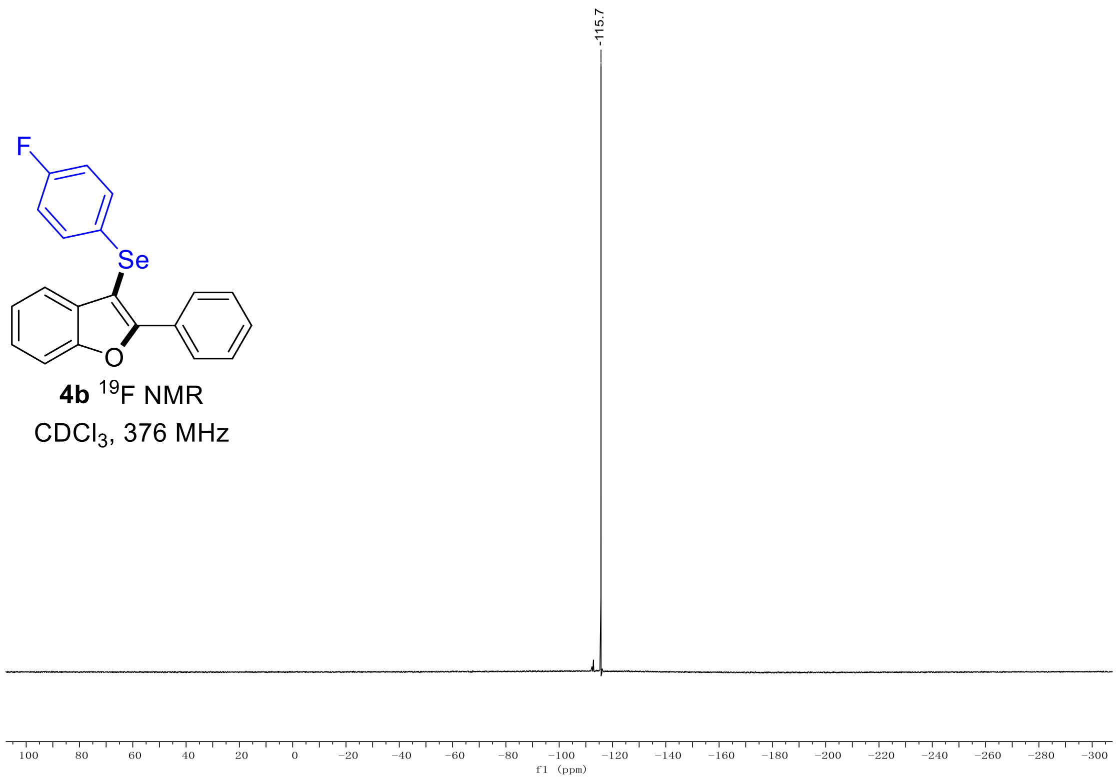

F
Se
O
4b 19F NMR
CDCl3, 376 MHz
-115.7
100 80 60 40 20 0 −20 −40 −60 −80 −100 −120 −140 −160 −180 −200 −220 −240 −260 −280 −300
f1 (ppm)

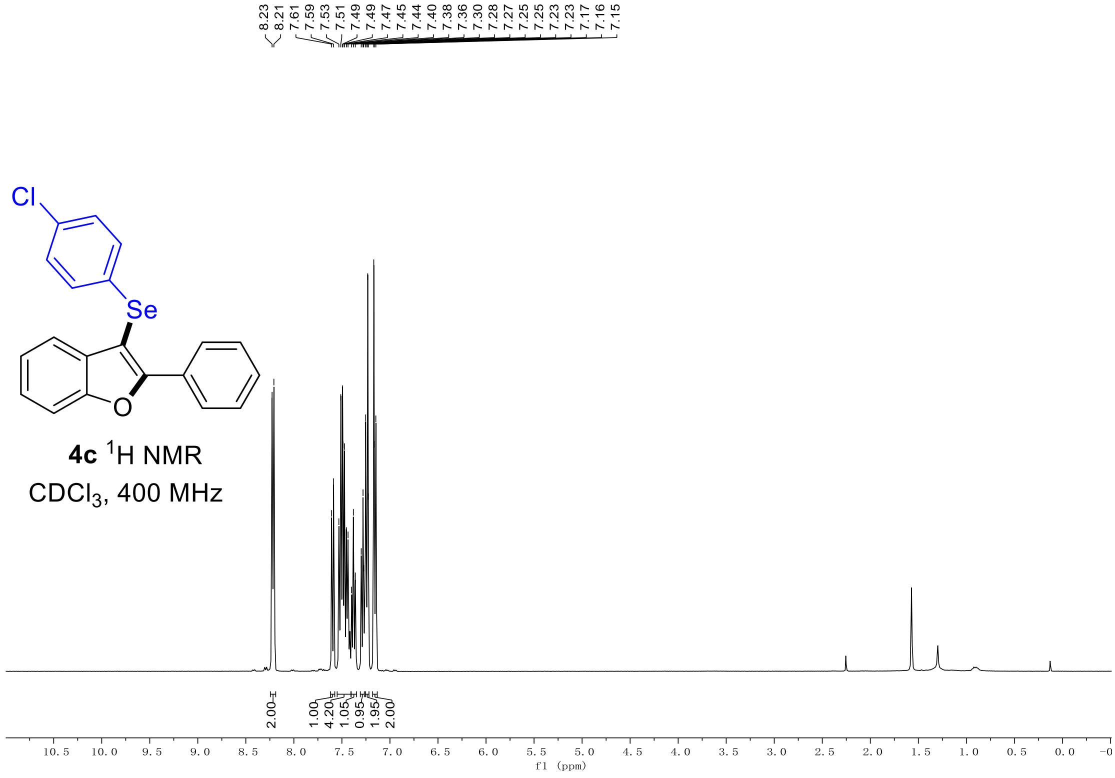

8.23
8.21
7.61
7.59
7.53
7.51
7.49
7.49
7.47
7.45
7.44
7.40
7.38
7.36
7.30
7.28
7.27
7.25
7.25
7.23
7.23
7.17
7.16
7.15
Cl
Se
O
4c 1H NMR
CDCl3, 400 MHz
2.00
1.00
4.20
1.05
0.95
1.95
2.00
10.5 10.0 9.5 9.0 8.5 8.0 7.5 7.0 6.5 6.0 5.5 5.0 4.5 4.0 3.5 3.0 2.5 2.0 1.5 1.0 0.5 0.0 −0
f1 (ppm)

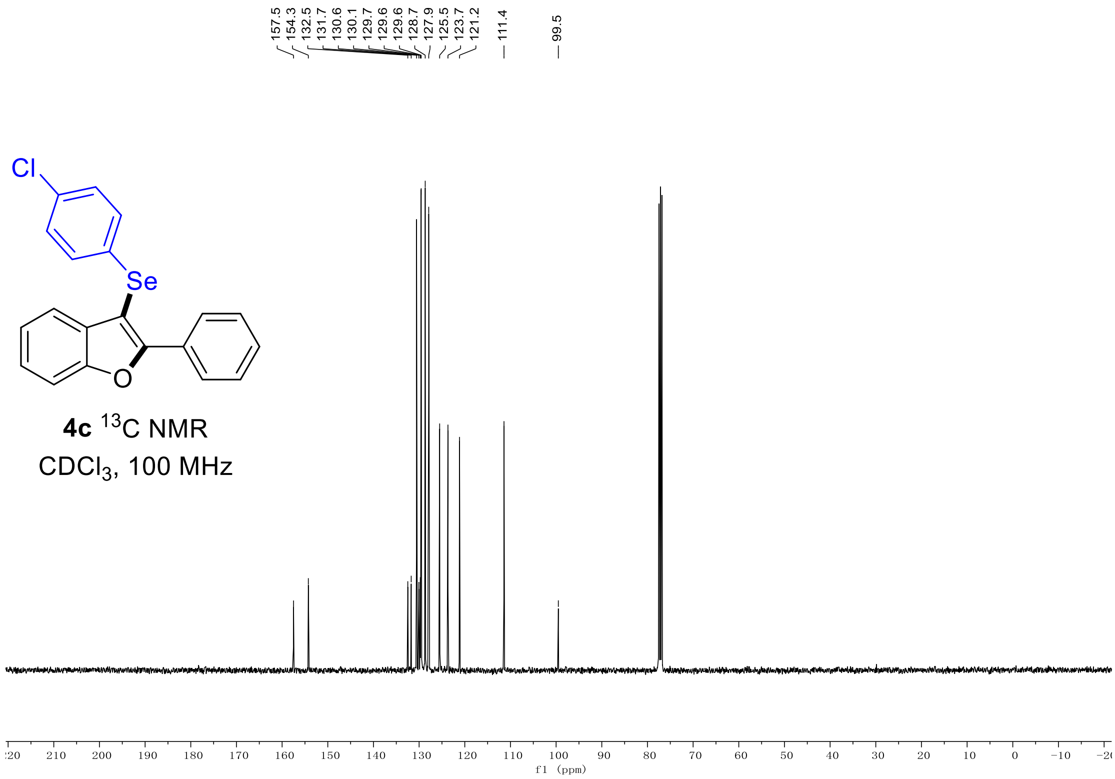
157.5
154.3
132.5
131.7
130.6
130.1
129.7
129.6
129.6
128.7
127.9
125.5
123.7
121.2
111.4
99.5
Cl
Se
O
4c 13C NMR
CDCl3, 100 MHz
f1 (ppm)

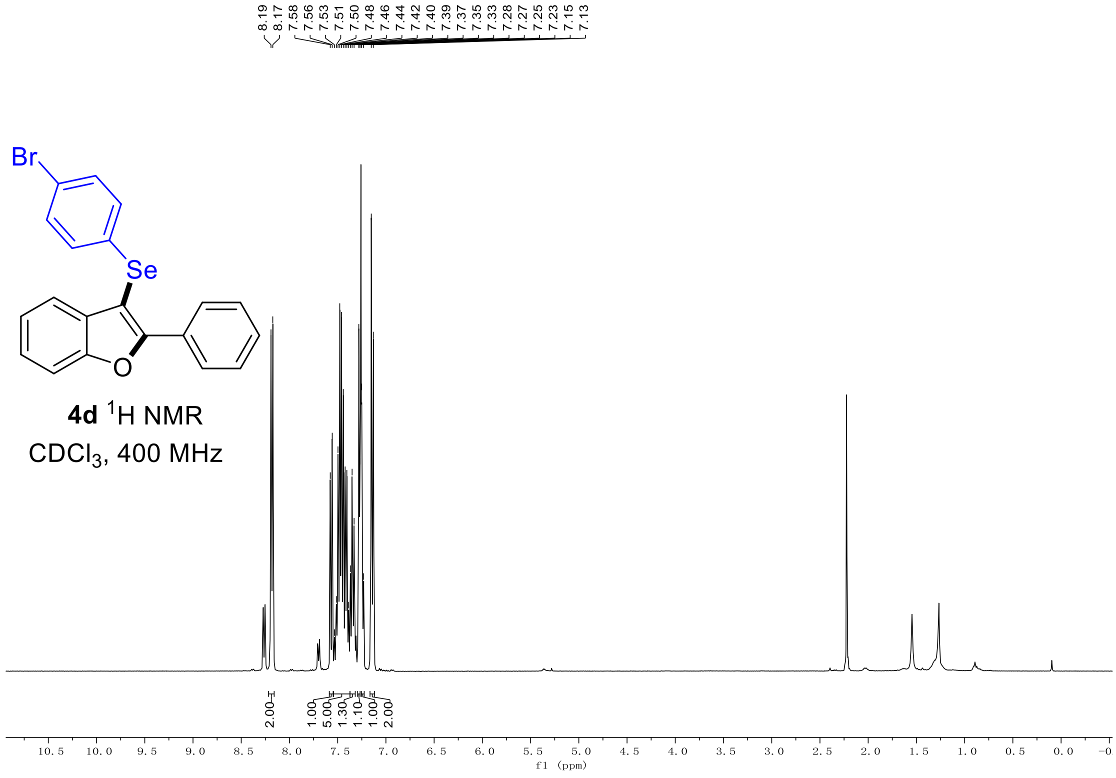
8.19
8.17
7.58
7.56
7.53
7.51
7.50
7.48
7.46
7.44
7.42
7.40
7.39
7.37
7.35
7.33
7.28
7.27
7.25
7.23
7.15
7.13
Br
Se
O
4d 1H NMR
CDCl3, 400 MHz
2.00
1.00
5.00
1.30
1.10
1.00
2.00
f1 (ppm)

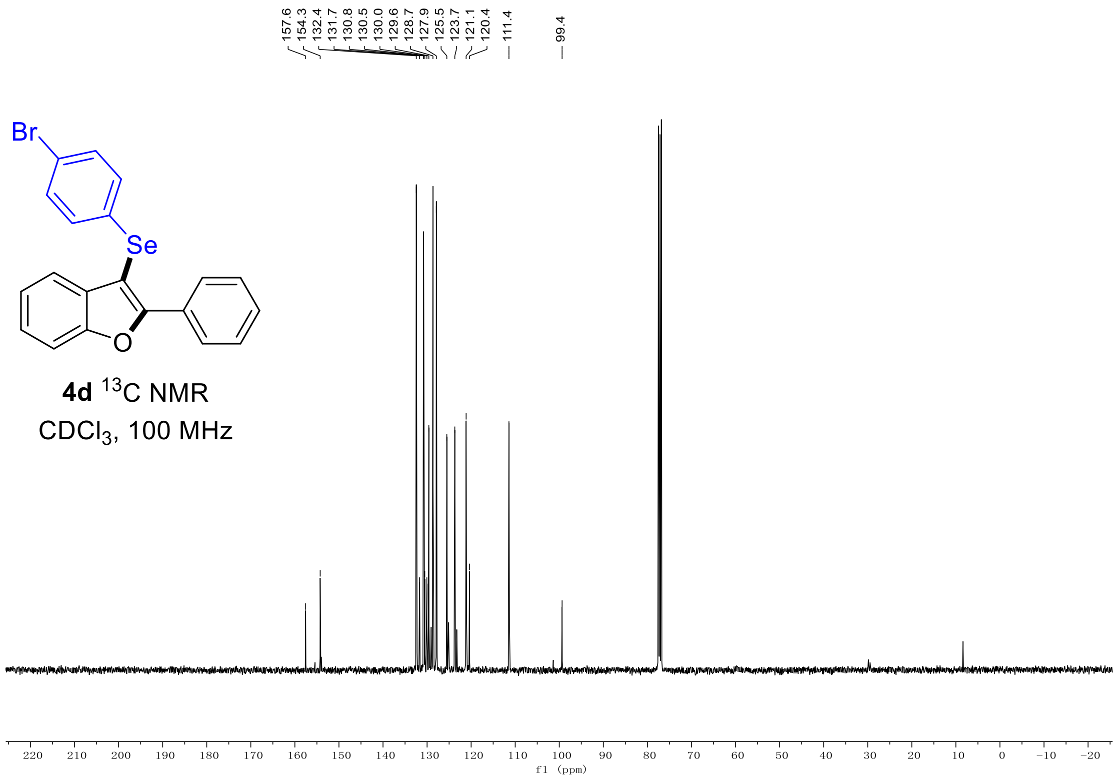
157.6
154.3
132.4
131.7
130.8
130.5
130.0
129.6
128.7
127.9
125.5
123.7
121.1
120.4
111.4
99.4
Br
Se
O
4d ^{13}C NMR
$CDCl_3$, 100 MHz
f1 (ppm)

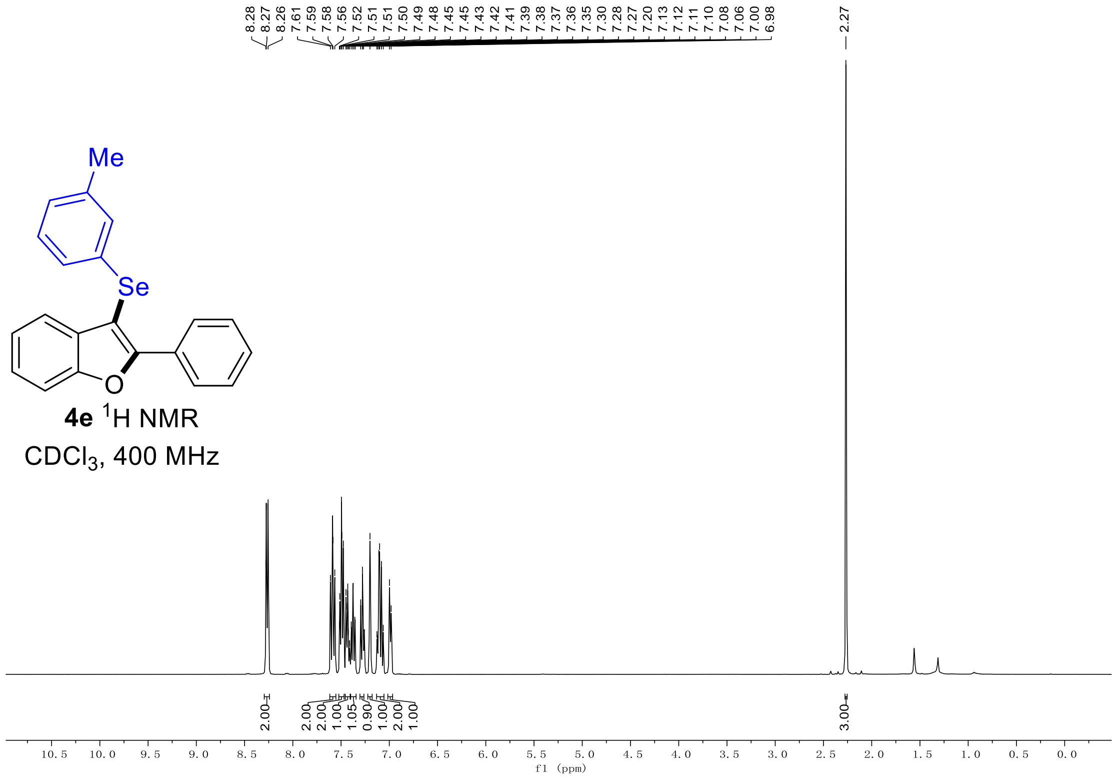
8.28
8.27
8.26
7.61
7.59
7.58
7.56
7.52
7.51
7.50
7.49
7.48
7.45
7.45
7.43
7.42
7.41
7.39
7.38
7.37
7.36
7.35
7.30
7.28
7.27
7.20
7.13
7.12
7.11
7.10
7.08
7.06
7.00
6.98
2.27
Me
Se
O
4e ^{1}H NMR
$CDCl_3$, 400 MHz
2.00
2.00
2.00
1.00
1.05
0.90
1.00
2.00
1.00
3.00
f1 (ppm)

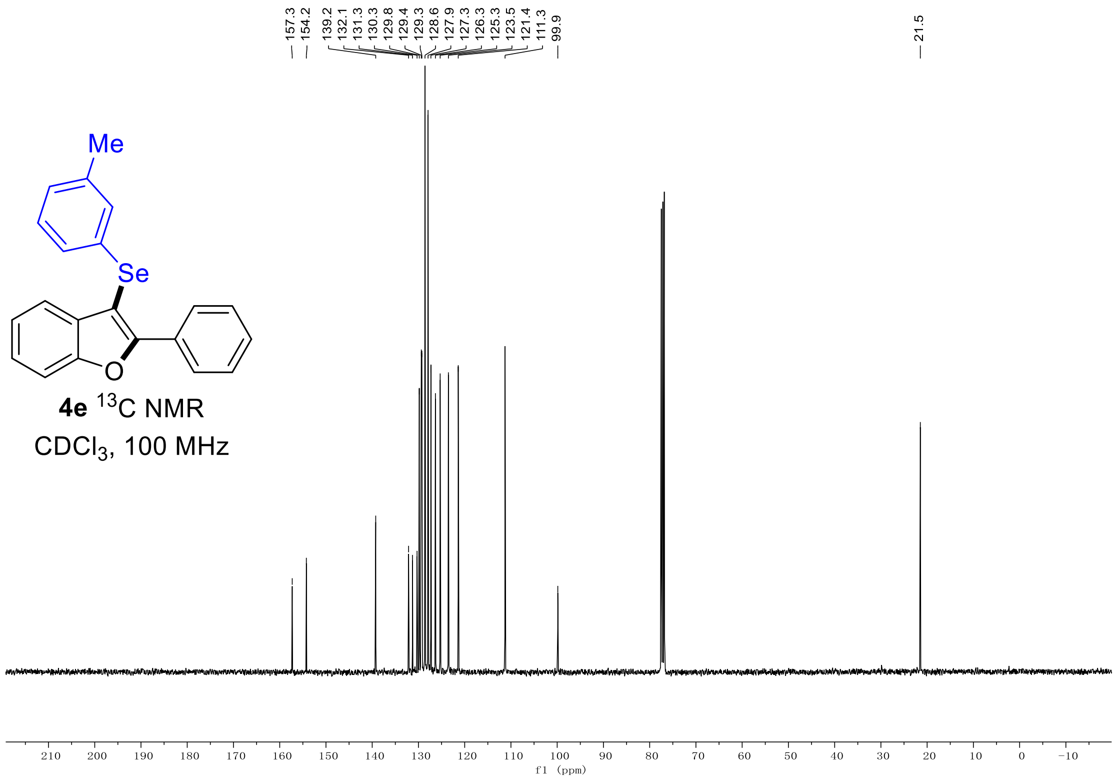
157.3
154.2
139.2
132.1
131.3
130.3
129.8
129.4
129.3
128.6
127.9
127.3
126.3
125.3
123.5
121.4
111.3
99.9
21.5
Me
Se
O
4e ¹³C NMR
CDCl₃, 100 MHz
f1 (ppm)

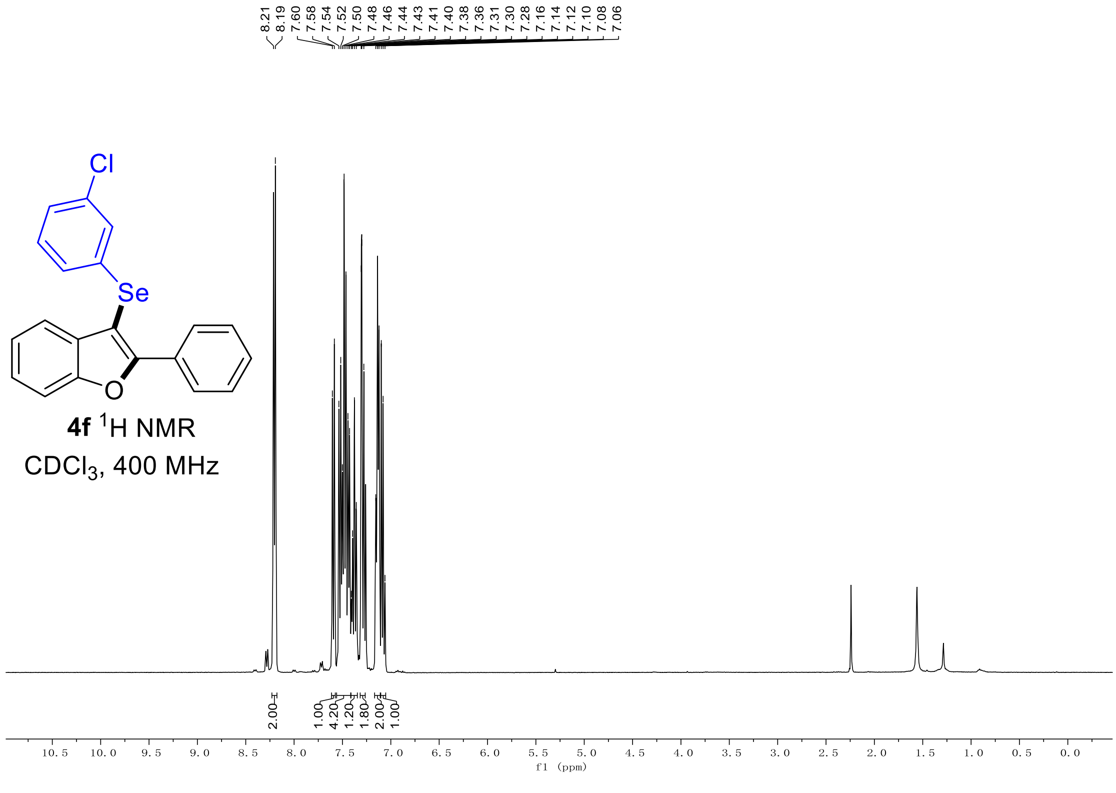
8.21
8.19
7.60
7.58
7.54
7.52
7.50
7.48
7.46
7.44
7.43
7.41
7.40
7.38
7.36
7.31
7.30
7.28
7.16
7.14
7.12
7.10
7.08
7.06
Cl
Se
O
4f ¹H NMR
CDCl₃, 400 MHz
2.00
1.00
4.20
1.20
1.80
2.00
1.00
f1 (ppm)

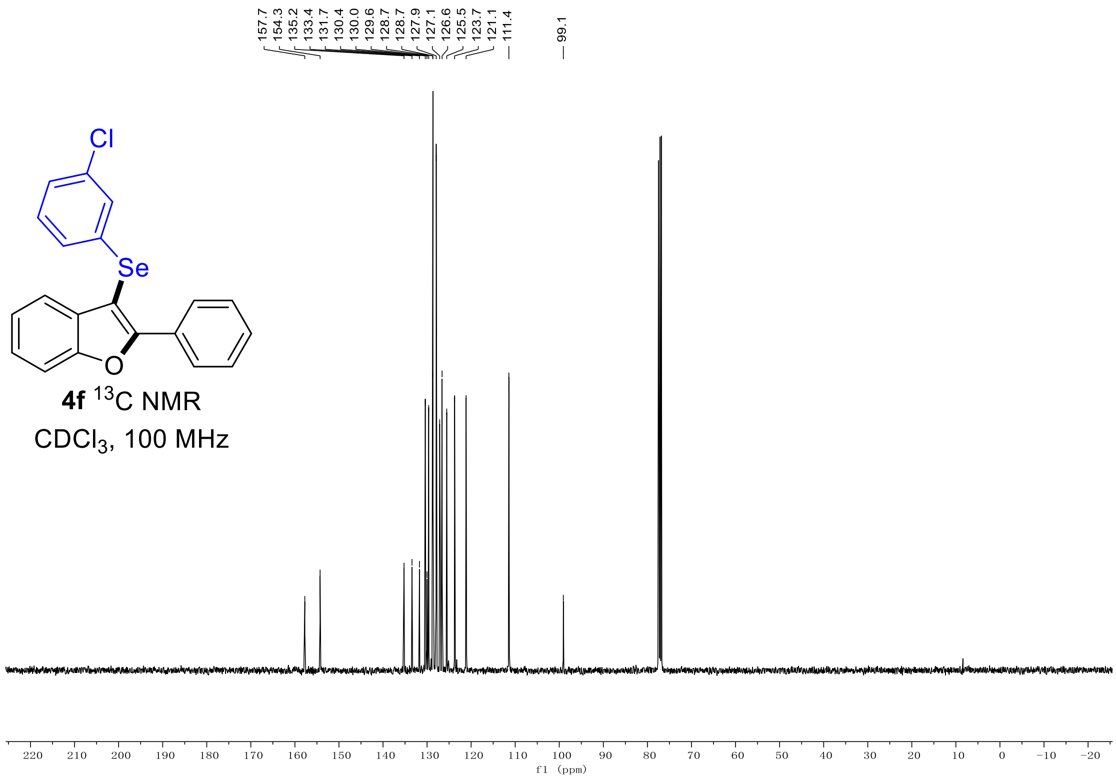
157.7
154.3
135.2
133.4
131.7
130.4
130.0
129.6
128.7
128.7
127.9
127.1
126.6
125.5
123.7
121.1
111.4
99.1
Cl
Se
O
4f 13C NMR
CDCl3, 100 MHz
f1 (ppm)

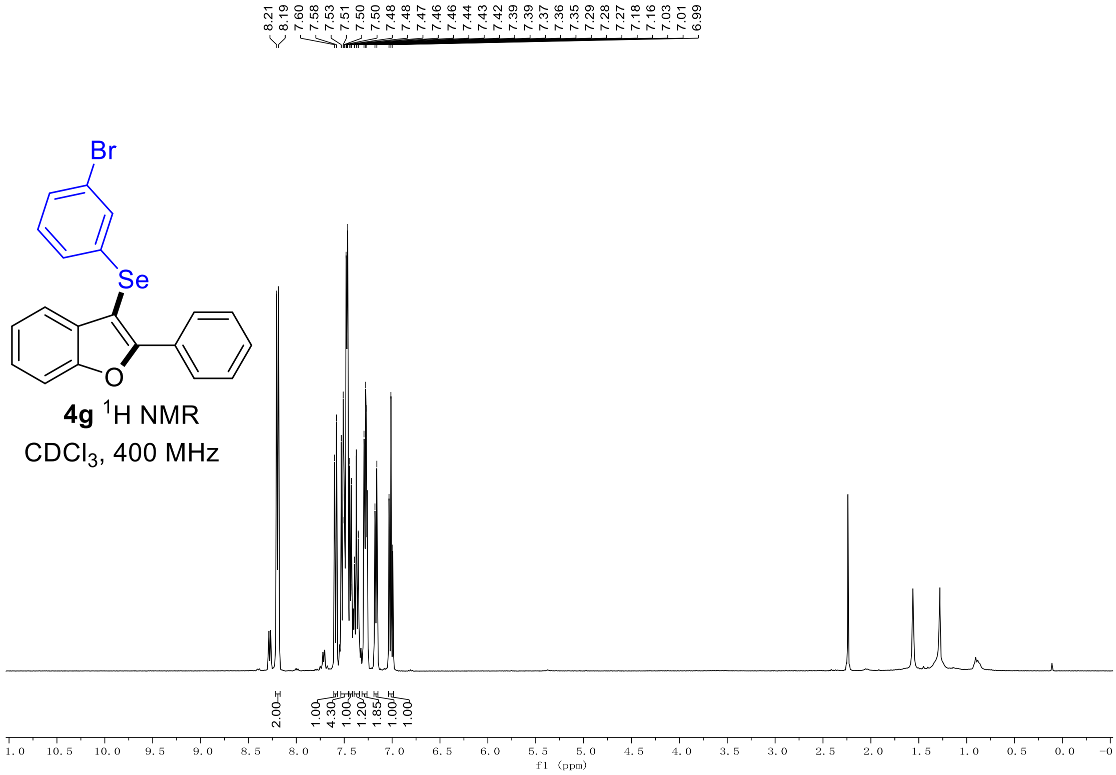
8.21
8.19
7.60
7.58
7.53
7.51
7.50
7.50
7.48
7.48
7.47
7.46
7.46
7.44
7.43
7.42
7.39
7.39
7.37
7.36
7.35
7.29
7.28
7.27
7.18
7.16
7.03
7.01
6.99
Br
Se
O
4g 1H NMR
CDCl3, 400 MHz
2.00
1.00
4.30
1.00
1.20
1.85
1.00
1.00
f1 (ppm)

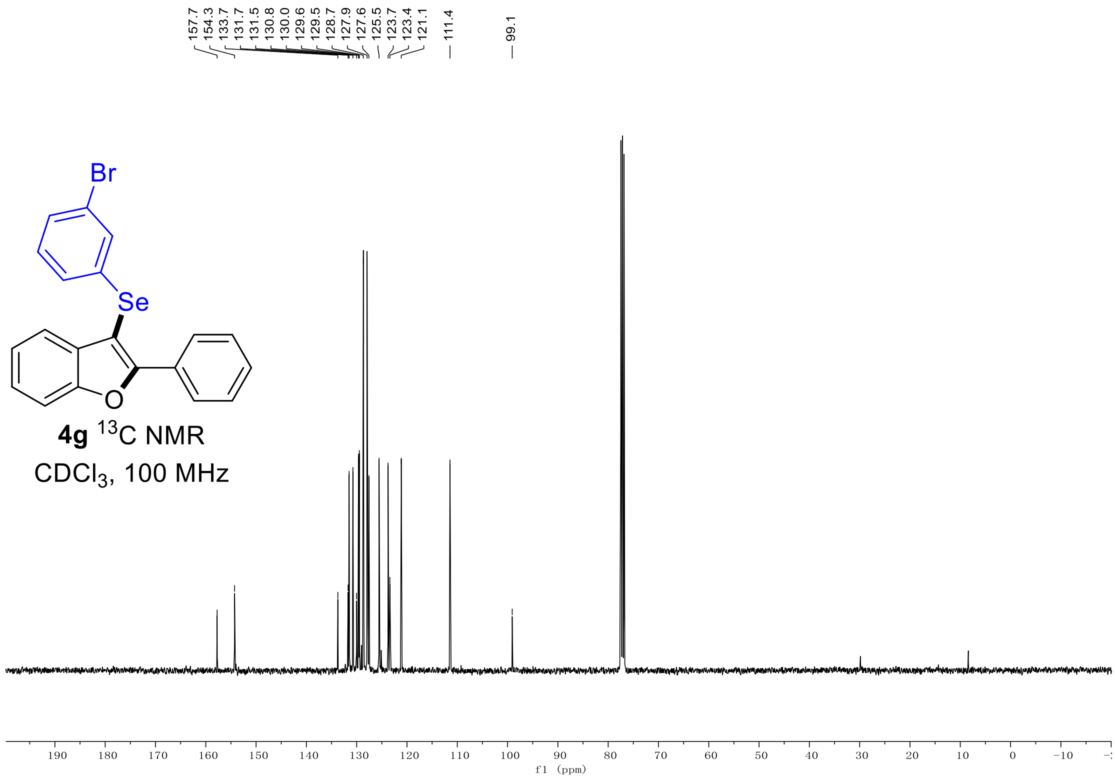
157.7
154.3
133.7
131.7
131.5
130.8
130.0
129.6
129.5
128.7
127.9
127.6
125.5
123.7
123.4
121.1
111.4
99.1
Br
Se
O
4g 13C NMR
CDCl3, 100 MHz
f1 (ppm)

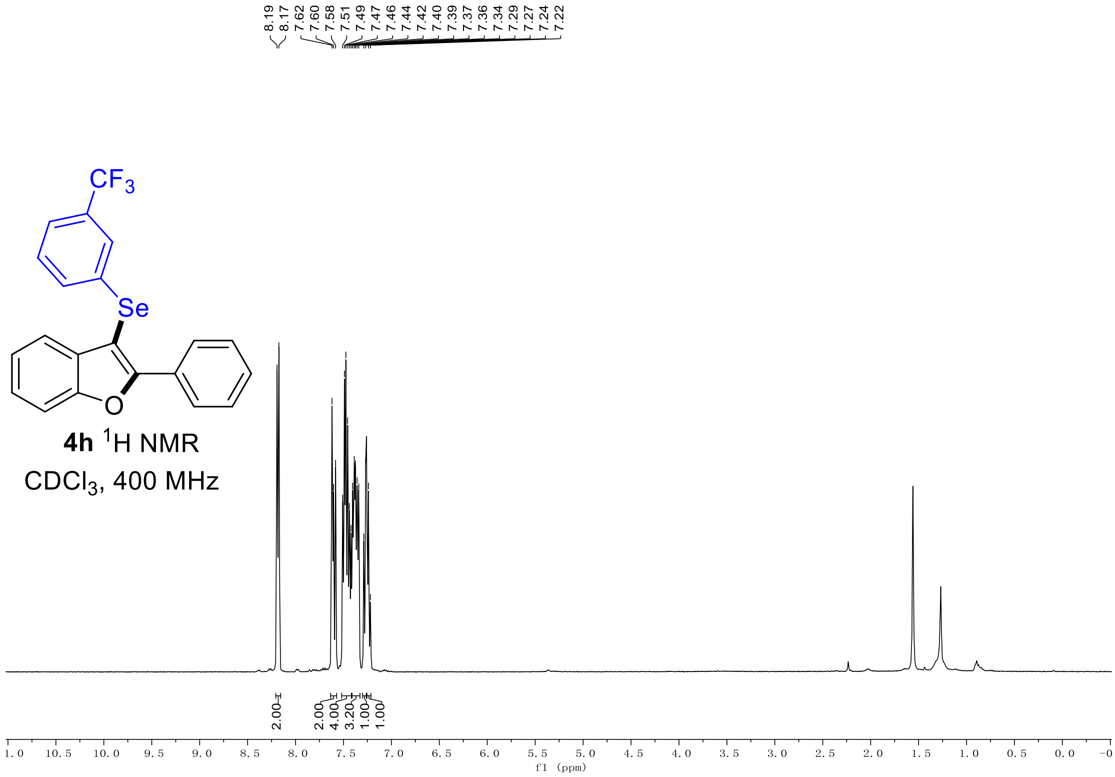
8.19
8.17
7.62
7.60
7.58
7.51
7.49
7.47
7.46
7.44
7.42
7.40
7.39
7.37
7.36
7.34
7.29
7.27
7.24
7.22
CF3
Se
O
4h 1H NMR
CDCl3, 400 MHz
2.00
2.00
4.00
3.20
1.00
1.00
f1 (ppm)

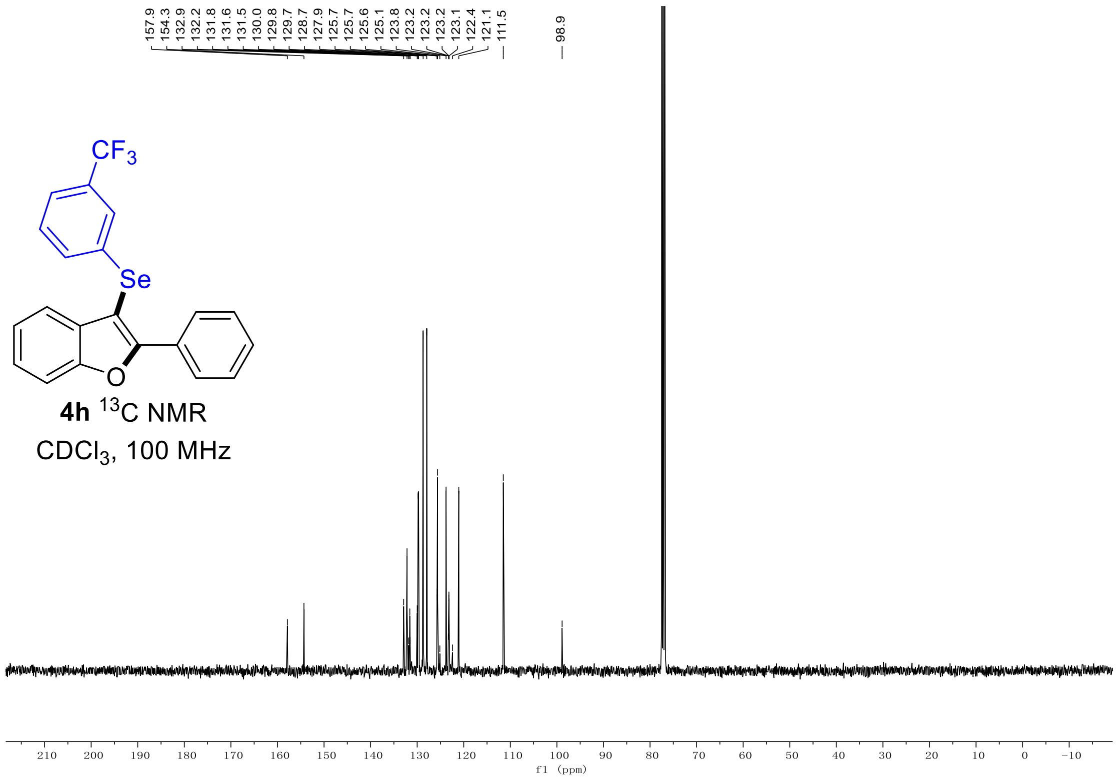
157.9
154.3
132.9
132.2
131.8
131.6
131.5
130.0
129.8
129.7
128.7
127.9
125.7
125.7
125.6
125.1
123.8
123.2
123.2
123.2
123.1
122.4
121.1
111.5
98.9
CF3
Se
O
4h 13C NMR
CDCl3, 100 MHz
210 200 190 180 170 160 150 140 130 120 110 100 90 80 70 60 50 40 30 20 10 0 -10
f1 (ppm)

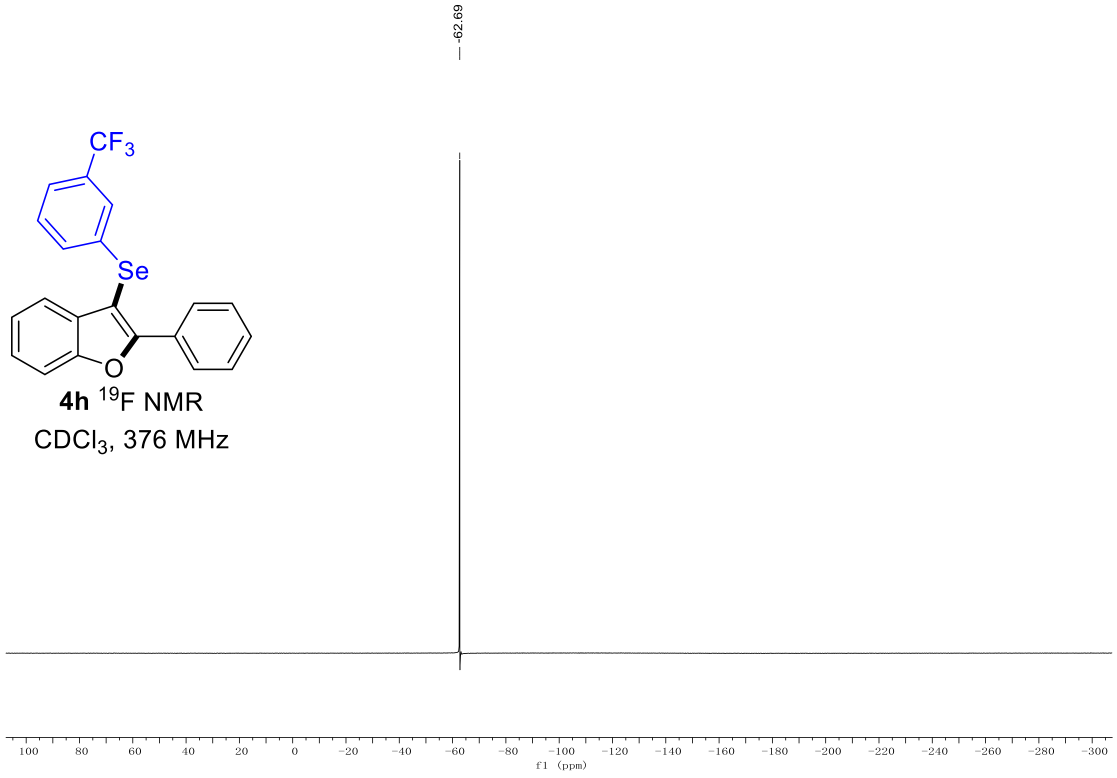
-62.69
CF3
Se
O
4h 19F NMR
CDCl3, 376 MHz
100 80 60 40 20 0 -20 -40 -60 -80 -100 -120 -140 -160 -180 -200 -220 -240 -260 -280 -300
f1 (ppm)

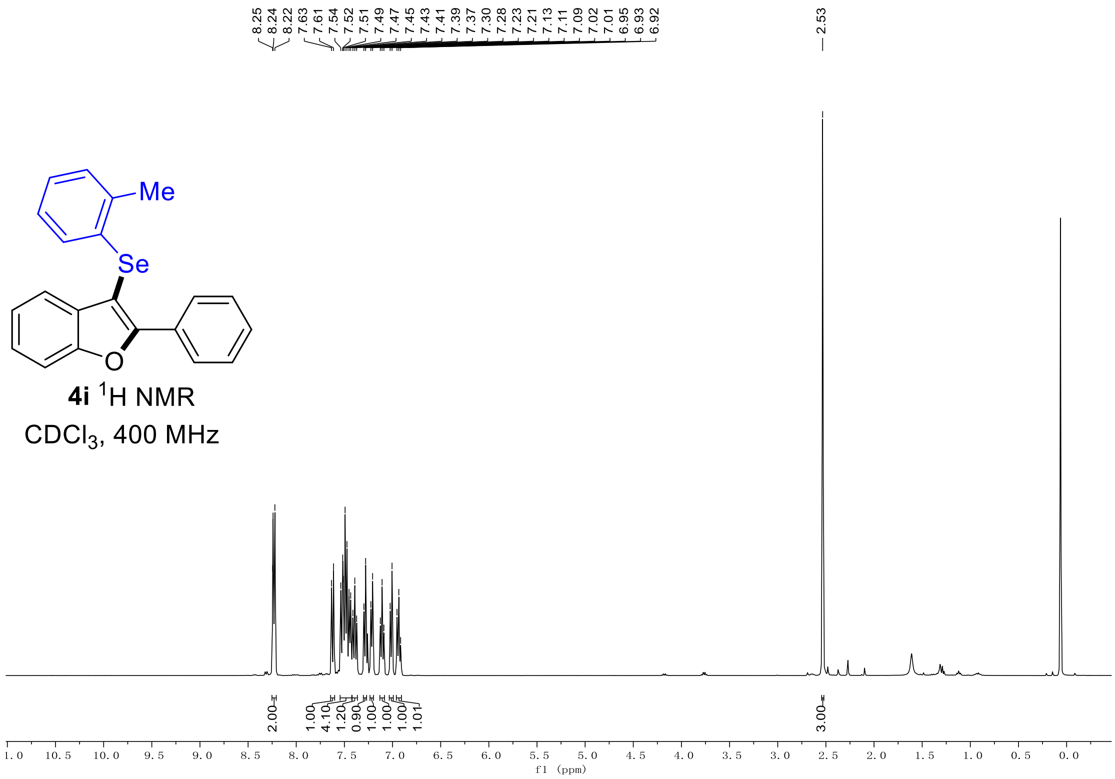
8.25
8.24
8.22
7.63
7.61
7.54
7.52
7.51
7.49
7.47
7.45
7.43
7.41
7.39
7.37
7.30
7.28
7.23
7.21
7.13
7.11
7.09
7.02
7.01
6.95
6.93
6.92
2.53
Me
Se
O
4i ¹H NMR
CDCl3, 400 MHz
2.00
1.00
4.10
1.20
0.90
1.00
1.00
1.00
1.01
3.00
f1 (ppm)

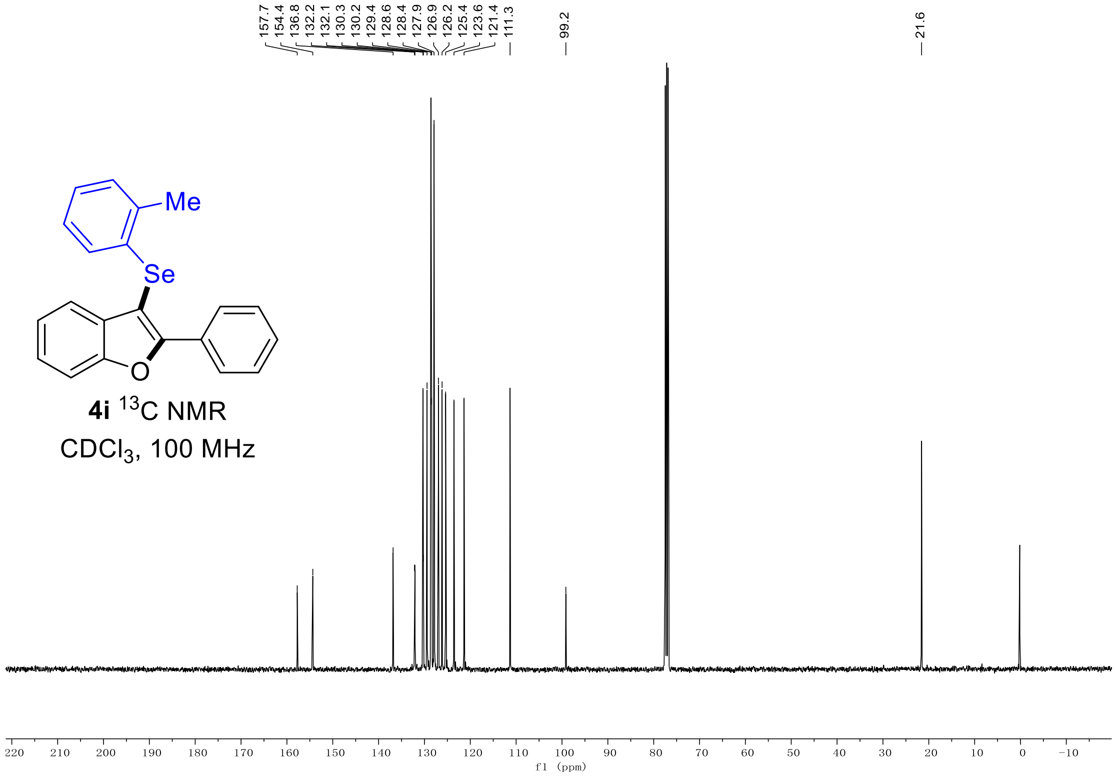
157.7
154.4
136.8
132.2
132.1
130.3
130.2
129.4
128.6
128.4
127.9
126.9
126.2
125.4
123.6
121.4
111.3
99.2
21.6
Me
Se
O
4i ¹³C NMR
CDCl3, 100 MHz
f1 (ppm)

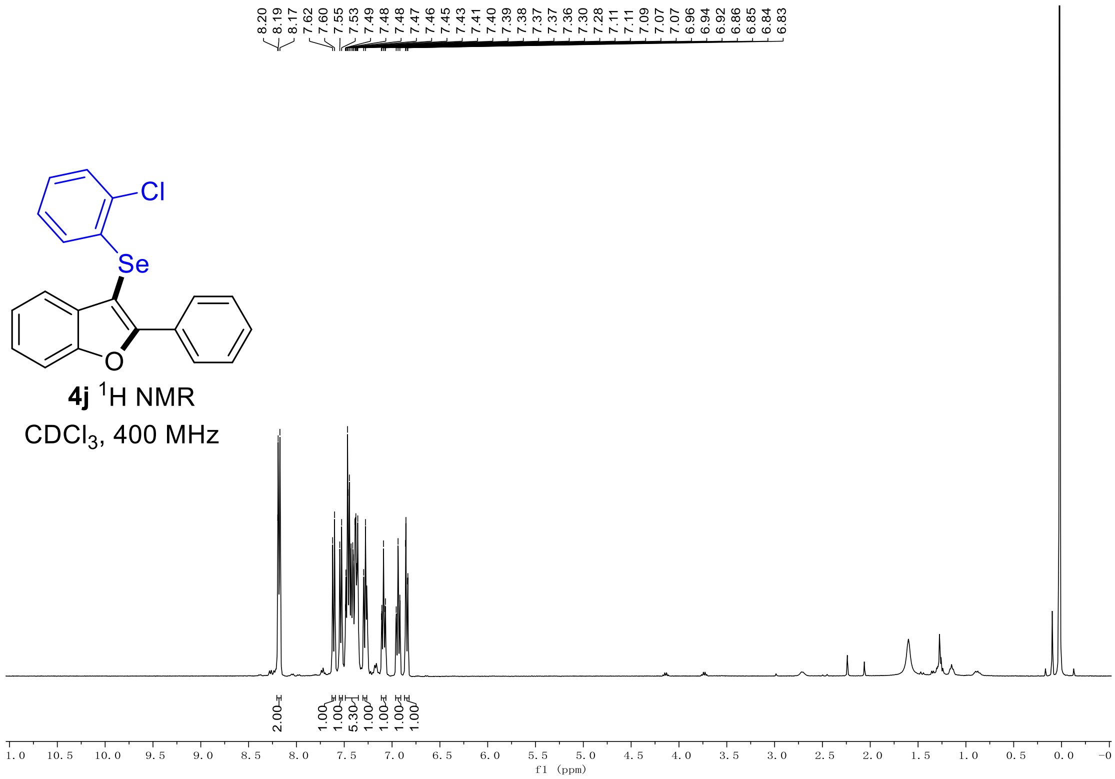
8.20
8.19
8.17
7.62
7.60
7.55
7.53
7.49
7.48
7.48
7.47
7.46
7.45
7.43
7.41
7.40
7.39
7.38
7.37
7.37
7.36
7.30
7.28
7.11
7.11
7.09
7.07
7.07
6.96
6.94
6.92
6.86
6.85
6.84
6.83
Cl
Se
O
4j ¹H NMR
CDCl₃, 400 MHz
2.00
1.00
1.00
1.00
5.30
1.00
1.00
1.00
1.00
1.00
f1 (ppm)

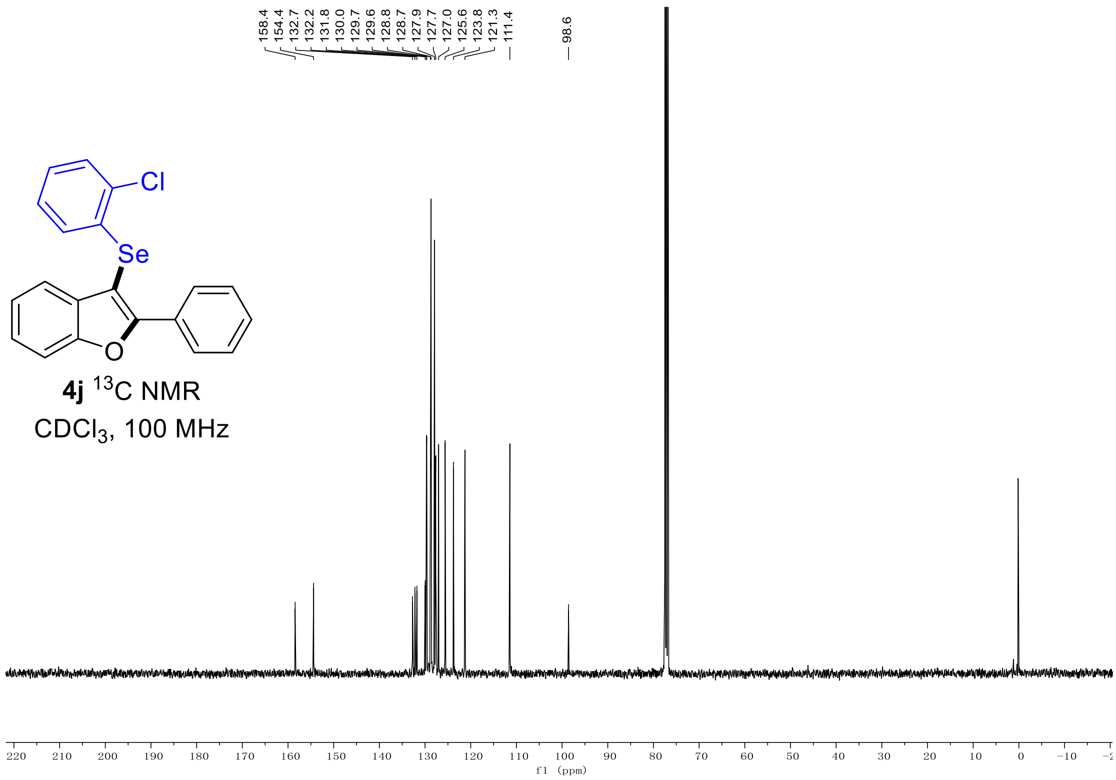
158.4
154.4
132.7
132.2
131.8
130.0
129.7
129.6
128.8
128.7
127.9
127.7
127.0
125.6
123.8
121.3
111.4
98.6
Cl
Se
O
4j ¹³C NMR
CDCl₃, 100 MHz
f1 (ppm)

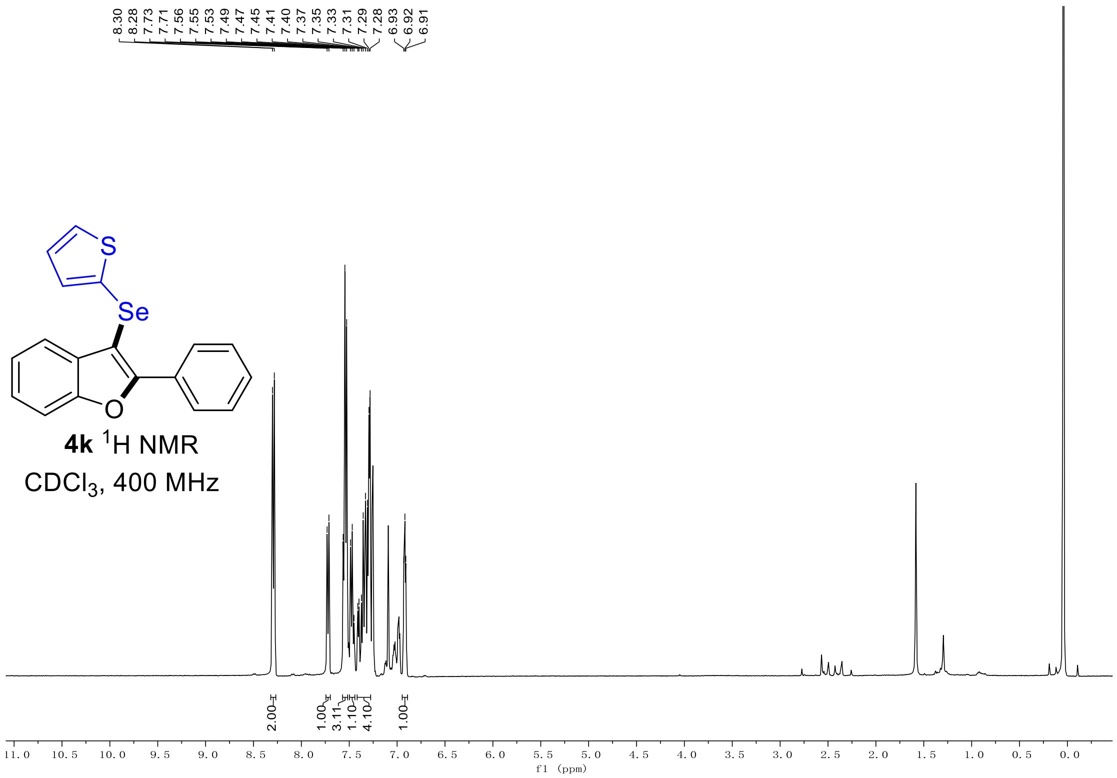

8.30 8.28 7.73 7.71 7.56 7.55 7.53 7.49 7.47 7.45 7.41 7.40 7.37 7.35 7.33 7.31 7.29 7.28 6.93 6.92 6.91
S
Se
O
4k 1H NMR
CDCl3, 400 MHz
2.00 1.00 3.11 1.10 4.10 1.00
f1 (ppm)

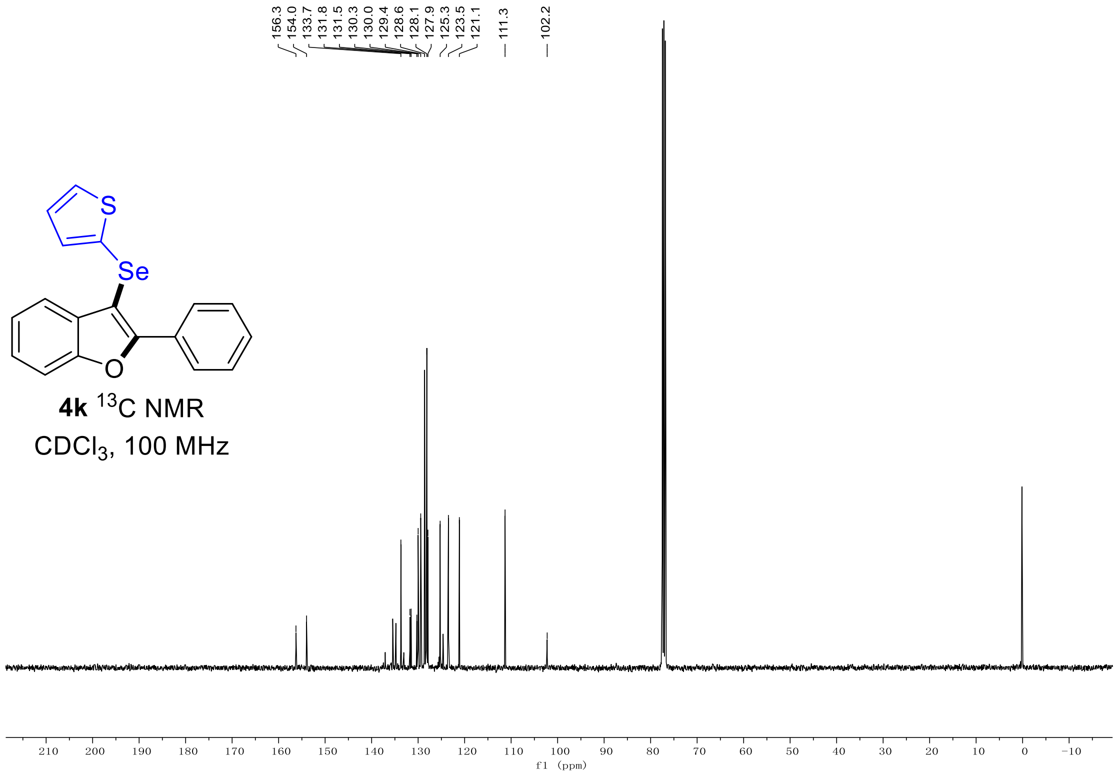

156.3 154.0 133.7 131.8 131.5 130.3 130.0 129.4 128.6 128.1 127.9 125.3 123.5 121.1 111.3 102.2
S
Se
O
4k 13C NMR
CDCl3, 100 MHz
f1 (ppm)

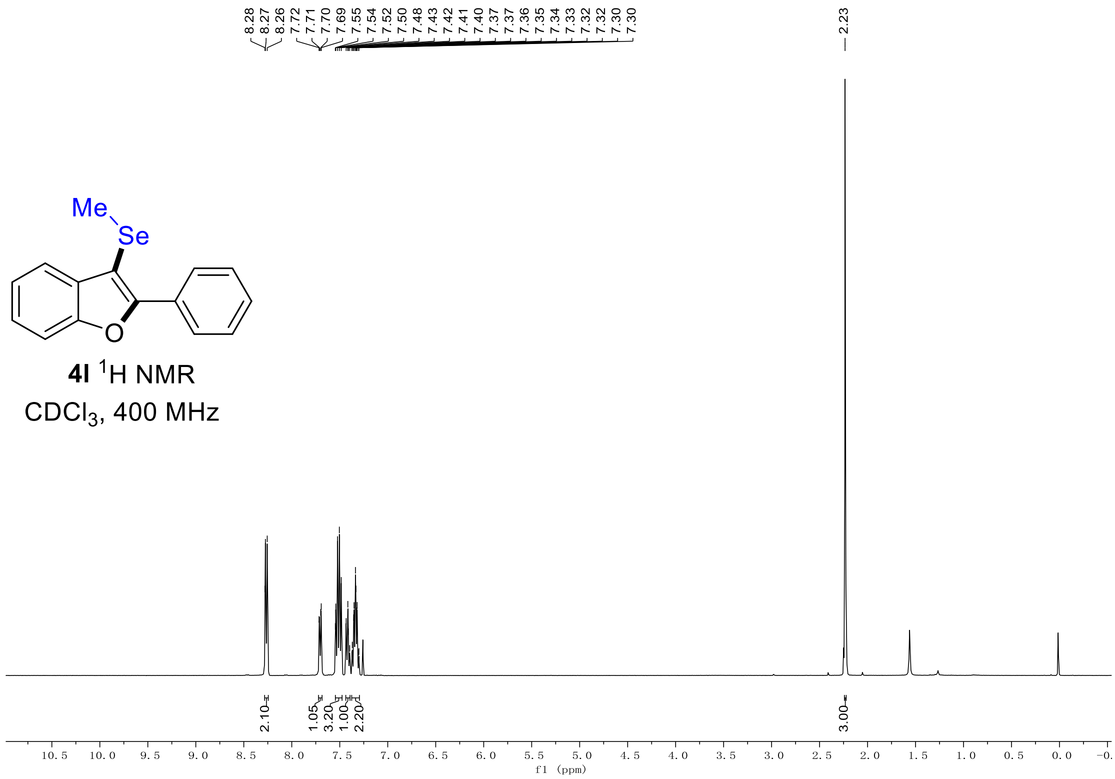

8.28
8.27
8.26
7.72
7.71
7.70
7.69
7.55
7.54
7.52
7.50
7.48
7.43
7.42
7.41
7.40
7.37
7.37
7.36
7.35
7.34
7.33
7.32
7.32
7.30
7.30
2.23
Me
Se
O
4l ^{1}H NMR
$CDCl_3$, 400 MHz
2.10
1.05
3.20
1.00
2.20
3.00
f1 (ppm)

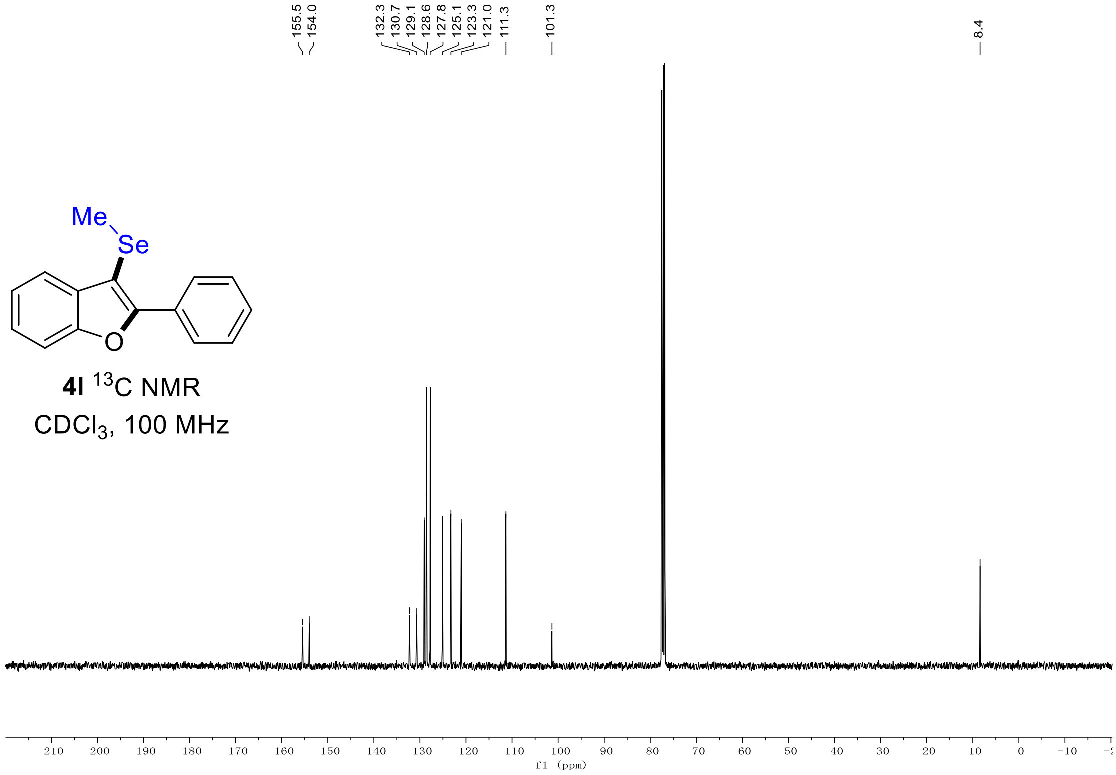

155.5
154.0
132.3
130.7
129.1
128.6
127.8
125.1
123.3
121.0
111.3
101.3
8.4
Me
Se
O
4l ^{13}C NMR
$CDCl_3$, 100 MHz
f1 (ppm)

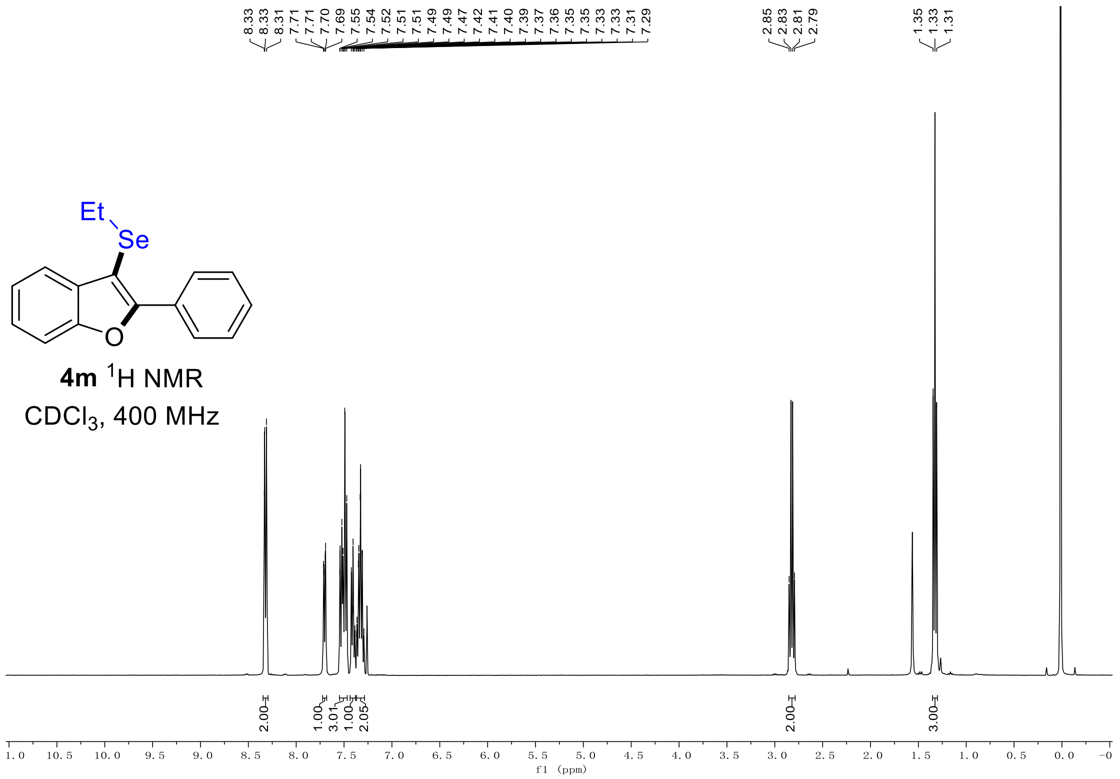
8.33 8.33 8.31 7.71 7.71 7.70 7.69 7.55 7.54 7.52 7.51 7.49 7.49 7.47 7.42 7.41 7.40 7.39 7.37 7.36 7.35 7.35 7.33 7.33 7.31 7.29
2.85 2.83 2.81 2.79
1.35 1.33 1.31
Et
Se
O
4m ¹H NMR
CDCl₃, 400 MHz
f1 (ppm)

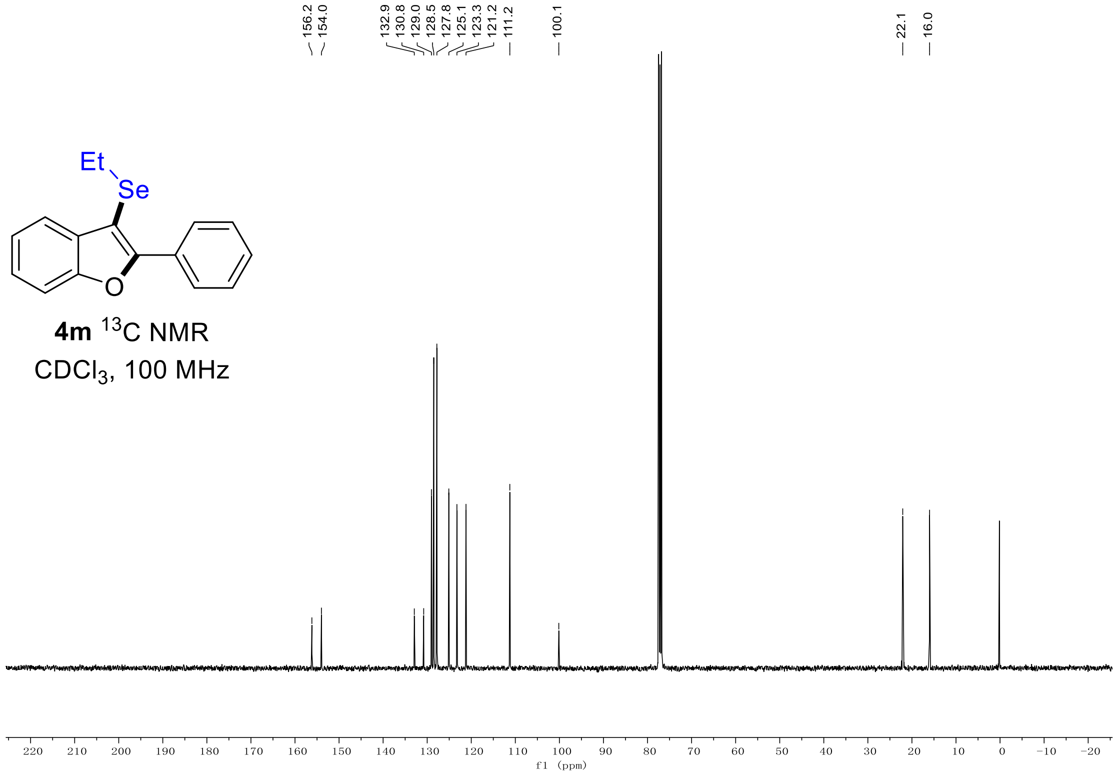
156.2 154.0
132.9 130.8 129.0 128.5 127.8 125.1 123.3 121.2 111.2
100.1
22.1 16.0
Et
Se
O
4m ¹³C NMR
CDCl₃, 100 MHz
f1 (ppm)
